# Taxonomic description curves of major lineages are influenced by biological and societal factors

**DOI:** 10.1038/s41598-025-29845-y

**Published:** 2025-11-24

**Authors:** David Schellenberger Costa, Martin Freiberg, Christian Wirth

**Affiliations:** 1 Systematic Botany and Functional Diversity Lab, Institute of Biology, Faculty of Life Sciences, Leipzig University, Johannisallee 21-23, 04103 Leipzig, Germany; 2 German Centre for Integrative Biodiversity Research (iDiv) Halle-Jena-Leipzig, Puschstr. 4, 04103 Leipzig, Germany; 3 Max-Planck-Institute for Biogeochemistry, Hans-Knöll-Str. 10, 07745 Jena, Germany

**Keywords:** LifeGate, Description curves, Phylogeny, Estimation, Diversity, Biodiversity in literature, Occurrence data, Computational biology and bioinformatics, Ecology, Ecology, Evolution, Zoology

## Abstract

Taxonomic description curves illustrate how the rate of new species descriptions changes over time. Their variability and overall shape are influenced by both biological properties and societal factors. Nevertheless, these curves are often used to estimate the total diversity of taxonomic groups. We extracted description dates for all major lineages of eukaryotic life from the LifeGate project, a comprehensive repository of phylogenetic information, to construct taxonomic description curves. Half-normal distributions were fitted to the empirical data to estimate the time required to reach 10% of current diversity, the total estimated diversity, and the residuals between empirical and fitted data. To investigate the determinants of variability in curve shapes, we compiled data on body size, soil-dwelling or endoparasitic life habits, aquatic versus terrestrial habitat, geographical distribution, author numbers, and public interest for the different taxonomic groups. Using a structural equation model, we linked variability in taxonomic description curves to major factors affecting discovery rates. We show that body size, author numbers, and, to a lesser extent, geographical range and preferred habitat, influence the shape of description curves, in addition to unique historical events. Our results caution against common approaches to diversity estimation, suggesting that this goal might be better achieved through simulations or by incorporating species distributions, rather than relying on description curves alone.

**Supplementary Information:**

The online version contains supplementary material available at 10.1038/s41598-025-29845-y.

## Introduction

The classification and description of living beings dates back at least to ancient Greece, when Aristotle collected shared and distinct characters of animal groups in his *Historia Animalum*^1^. Modern taxonomic nomenclature of animals and plants builds on Carl von Linné’s *Systema Naturae*^2^ and *Species plantarum*^3^, respectively. A system to classify and order the living world is a prerequisite for understanding it, but actual discoveries and descriptions depend on time and effort invested. Description rates are influenced by both biological properties of the organisms under study, such as their size or distribution, and societal factors, e.g., the scientific and public interest, the overall conditions for carrying out taxonomic research, and technological advances.

Description rates have been used to estimate true diversity^4–6^. Early methods extrapolated fitted lines from available data, but more recent approaches seek to explain these curves mechanistically using predictors^7^. Other attempts included inferences drawn from the relative number of taxa across taxonomic levels^8^ or species-area curves^9^. All these methods face challenges due to unknown or variable key drivers, such as the probability of discovering species, numbers of taxonomists working on specific groups in the future, distributions of species in area-based approaches, and differences in the relatedness of members of the same taxonomic levels across the taxonomy in phylogenetically informed approaches.

The probability of discovering species varies significantly across taxonomic groups. Size plays a key role, as large species are hard to overlook, while small organisms may remain undiscovered even in accessible habitats^10^. Some environments are harder to sample than others; e.g., soil-dwelling organisms or endoparasites cannot be found by simple observation, but require elaborate methods to detect them. Aquatic habitats generally pose greater challenges than terrestrial ones. Geographical range, with the Northern hemisphere, i.e. Europe, the USA, Canada, and Russia, harbouring long-standing centres of taxonomic research, certainly also influenced discoveries^11,12^.

Societal factors also influence description rates. The number of taxonomists is often linked to a society’s ability and willingness to fund their work^13,14^. While taxonomy profits from essential contributions of amateur and citizen scientists, and is not as dependent on expensive laboratory equipment or computing power as other disciplines, securing resources for sampling expeditions and the maintenance of collections was and is essential in this field. Funding may be driven by economic benefits with new discoveries being a by-product of commercial explorations^15^. On the contrary, taxonomic work is often impeded during crises when funding and resources are scarce.

Like biological properties, societal factors vary between countries and taxonomic groups; for example, aquatic vertebrates are often collected through fishing, while foraminiferans in the same habitats are not and thus need targeted sampling efforts.

To elucidate the drivers of description curves, we used data from the LifeGate project, which aims to phylogenetically present all eukaryotic life in a consistent framework (https://lifegate.idiv.de). We extracted description dates of all taxa, and identified major lineages for analysis. To our knowledge, this is the first presentation of description rates of this taxonomic scope to date. We also collected data for important predictors of description rates, such as public interest, author numbers, geographic distribution, soil-dwelling/endoparasitic species, aquatic habitat use, and body size for all groups. We hypothesized that cumulative description curves are shaped by biological and societal factors. More specifically, we expected predictors to affect curve shape in three ways: determining the initial descriptions time, i.e. the time until 10% of the currently known species were described, the future descriptions ratio, i.e. the ratio between estimated and current descriptions of a group, and description curve residuals, i.e. the sum of squares calculated between the cumulative description curves and the actual descriptions. We tested the latter using a Bayesian structural equation model. In addition, we investigated whether singular historic events (e.g., wars, or landmark publications) could explain synchronous changes in description rates across taxonomic groups.

## Methods

Data collection, analysis, and figure creation were primarily conducted in R^16^.

### Data collection

#### Species description dates and current description numbers

Dates of species descriptions were extracted from the LifeGate dataset (https://lifegate.idiv.de), along with author names. In LifeGate, only taxa that were non-extinct at the time of their description are considered, and special care is taken to sort out dubious cases (especially foraminiferans and molluscs). LifeGate scans the primary literature to identify synonymy, with an emphasis on animal taxa, where this is often complicated due to missing new combination authors. It explicitly accounts for changes in the valid names of taxa and provides the publication date of the *protologue* in all cases. Major lineages with current description numbers of > 400 extant species were selected for our analysis. Classification was performed at the phylum level, with the exception of the large group of arthropods, for which we investigated classes, and in the case of insects, orders. We made this decision because insects include the most species-rich groups of living beings and have very different description histories. To make sure this choice does not affect our results and interpretation, we also ran our analyses aggregating insects into the class Insecta, but as the results were qualitatively similar, we only report them in the Supplementary Information. As current nomenclature is based on Linné^3^, descriptions start in 1753, and end in 2017, the last year for which complete taxonomic information for all groups was included into LifeGate at the time of writing. For two groups where internal revisions of species descriptions in LifeGate were not yet complete, we compared LifeGate data with data from the Catalogue of Life^17^ and GBIF^18^. As LifeGate had considerably more species descriptions listed for Lepidoptera and Bryophyta than present in the other databases, we reduced species descriptions from LifeGate to those found either in Catalogue of Life or GBIF.

#### Author numbers

Author numbers were also extracted from the LifeGate dataset by counting unique author names for each year from 1753 to 2017. Mean author numbers for each taxonomic group were obtained by dividing the number of unique author names by the number of years investigated.

#### Body size, fractions of soil-dwelling/endoparasitic, aquatic species

Data on average body size, the fractions of soil-dwelling/endoparasitic species, and of aquatic species within a group were extracted from a number of online sources, mainly Wikipedia (Suppl. Table 1, sheet “sources”). The information provided on these variables for large taxonomic groups is quite coarse (e.g., “body length of 10–60 cm”, “mostly aquatic”). Individual measures per species are mostly unavailable, or only available for a subset of the species from a group. Therefore, for body size, we estimated the average body size based on the available data, and classified it into size classes, each spanning one order of magnitude. The range of body size in our data was from − 3 (Microsporidia, Haptophyta) to 2 (Tracheophyta, Rhodophyta, Chordata), corresponding to average body sizes of (1–10)*10^− 3^mm = 1–10 μm and (1–10)*10^2^mm = 1–10 dm, respectively. For the fractions of soil-dwelling/endoparasitic and aquatic species, we used ordinal scales from 1 = no soil-dwelling/endoparasitic species to 5 = all species soil-dwelling/endoparasitic and 1 = no aquatic species to 5 = all species aquatic. Intermediate numbers were used when finding keywords like “occasionally”, “some species”, “few” (2); “half of the species”, “occur both in”, “common in both” (3); or “mostly”, “the majority”, “a large number of species” (4) in group descriptions.

#### Occurrences in Europe, the USA, Canada, and Russia

We used the GBIF occurrence API^18^ to obtain information on the fraction of species of a taxonomic group found in Europe, the USA, Canada, and Russia. We are aware that the occurrence information in GBIF has limitations: it does not necessarily show the complete occurrence range of species, and individual records may be misidentifications or being taken under non-natural conditions. However, for an analysis of this scale, we expect the error introduced to be minor. To obtain the data, we ran two queries: one for the mentioned countries (splitting Europe into individual countries for comparability), and one using all countries used in GBIF, retrieved from the GBIF API documentation. To extract species numbers easily, we used ‘speciesKey’ representing individual species as a facet parameter. A facet parameter groups query results by their parameter value, i.e. the speciesKey in our case. As a result, we got occurrences of each species within our subset of Europe, the USA, Canada, and Russia, and within any country worldwide. We then divided the number of distinct speciesKeys from our subset by the worldwide total for each group. As there is data without sampling coordinates and with sampling coordinates falling outside country boundaries, i.e. into the open sea, we tested their influence on the ratios obtained before. We first compared the effect of limiting occurrences to those with coordinates. There was little change in the overall pattern and therefore, we used the full dataset. Second, we compared the sum of per-country records to occurrences retrieved for the whole world, including open sea-records. In any group, they accounted for less than 10% of the data (see Suppl. Table 2 for exact numbers), which is why we are confident our decision to ignore records outside country borders did not affect our analysis.

#### Public interest data

To obtain a proxy of public interest, we extracted data from the Biodiversity in Literature (BiL) project^19^ using the R package RSelenium^20^. The BiL project collected occurrences of vernacular names of living beings in a corpus of literature from the Gutenberg project, spanning a time range from 1705 to 1969 and including about 16,000 books^21^. These names refer to different taxonomic levels, from infraspecies to phyla. We chose to treat all names in the same manner and only counted those that could be classified into our taxonomic groups. We first downloaded a table including the vernacular and scientific names identified by the BiL project (https://github.com/NoHara42/BachelorThesis/), and then used this data to source the number of occurrences from the BiL explorer (http://ch01.informatik.uni-leipzig.de:5100/bil-explorer/, currently only working from within Leipzig University). We used the GBIF species API (https://techdocs.gbif.org/en/openapi/v1/species), to classify both scientific and vernacular names from the BiL dataset into our taxonomic groups, adding “qField = vernacular” to the API call in case of vernacular names. We compared results and manually checked name with differences in results for vernacular and scientific names, as well as names that were ambiguous.

### Data analysis

#### Phylogenetic tree

**Fig. 1:**
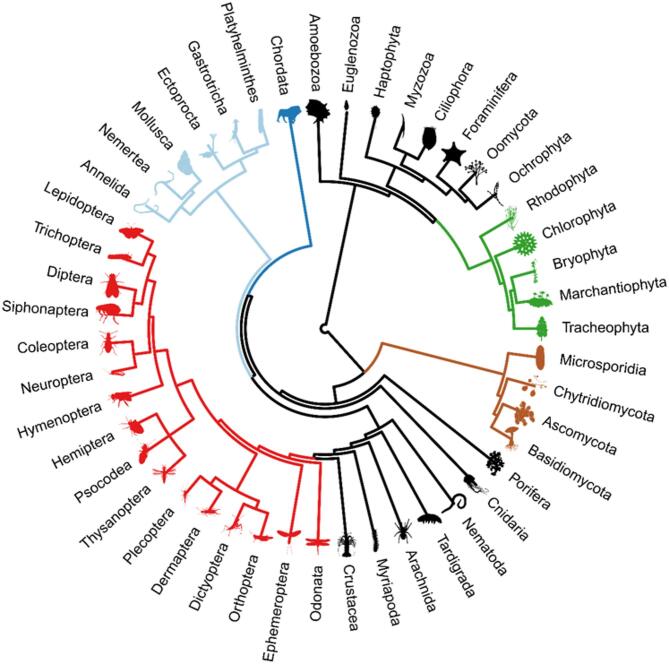
Phylogeny of the taxonomic groups covered in this study. Colours indicate higher taxonomic groups: green: plants *sensu lato*, brown: fungi, red: insects, light blue: Spiralia, dark blue: Chordata.

We placed the taxonomic groups into a phylogenetic tree to show their kin relationships (Fig. 1). The tree was created using data from the Open Tree Taxonomy (https://tree.opentreeoflife.org) and the rotl package in R for data retrieval^22^. Tree display depends on specific names being found as nodes or tips of the tree. In the case of taxonomic groups whose names were ambiguous or not found in the tree, we searched for individual species names from the respective groups present in the Open Tree Taxonomy backbone and used the respective nodes or tips for placing the respective taxonomic group. Images of specimens from the different taxonomic groups are in the public domain and were retrieved from PhyloPic (https://www.phylopic.org) using the R package rphylotopic^23^.

#### Cumulative description curves

We tested several approximation functions for the cumulative descriptions, i.e. integrated description rates: a half-normal (left-sided) distribution function, a customised Bertalanffy function and the Gompertz function (see code). The three functions were selected as they have a fixed maximum, representing the finite number of species in each taxonomic group. As the normal distribution declines after the maximum, we modified the formula so that for x values larger than the mean, y values remained constant. We also added a constant to the scale parameter so that the curve maximum could not be less than the actual data. The formula for the normal distribution was:

$$D=\left(\frac{k}{a^*\:\sqrt{2^*\pi}}+1\right)*\left(e^{{-\frac{1}{2}\left(\frac{Y-b}{a}\right)}^{2}}\right)*\left(Y\:\le\:\:b\right)\:+\:(Y\:>\:b))$$

where *D* is the number of descriptions, *Y* is the year, *k* is the scale parameter, *a* is the standard deviation and *b* is the mean of the normal distribution. Note that the terms *Y* ≤ *b* and *Y* > *b* are evaluated logically and then converted to 0 or 1, respectively. As a result, for years *Y* smaller or equal than the mean of the distribution, *D* is calculated as expected in the standard normal distribution. For years larger than the mean, the scale term $\:\frac{k}{a\:*\:\sqrt{2*\pi\:}}+1$ is simply multiplied by one, resulting in the maximum value for *D*, as e^− x^ ≤ 1. The plus one added to the scale term makes sure *D* has a maximum ≥ 1, avoiding fits with a maximum smaller than the empirical maximum, i.e. the maximum of the data.

All three functions fitted the data well. We chose the half-normal distribution as it provided the closest estimates of total species numbers compared to former studies. Fitting was performed using a Bayesian approach with the R package rethinking^24^. The mean and standard deviation of the fitted functions were used to extract the time until 10% of current descriptions were reached (initial description time) and the ratio of the estimated and current number of descriptions (future descriptions ratio). Description curve residuals were calculated as the sum of squares between the observed and predicted number of descriptions (Suppl. Fig. 1).

#### Description rate anomalies

To assess idiosyncratic changes of description rates, we used a moving-window approach, comparing five-year intervals with each other. We classified a yearly description rate as particularly low or high if the mean of the preceding five years was at least double or less than half the mean of the particular year and the following four years and if the preceding five years were not already classified the opposite way. We identified unusual peaks by visual inspection and searched for coinciding historical events in the respective years, showing them in the corresponding figure.

#### Structural equation model

We created a Bayesian structural equation model using the bsem() function from the R package blavaan^25^ based on a set of hypotheses linking the predictors and curve parameters (Table 1). Predictors and curve parameters were scaled to [0,1] to increase model stability and improve model convergence with standard priors. We ran the model and assessed model fit by investigating trace plots of all parameters and the posterior predictive p value^26^.

**Table 1:** SEM hypotheses.

Response	Predictor	Expectation	Explanation
Public interest	Current descriptions	+	Current descriptions are the number of species known by humankind, and high totals equal high visibility and relevance
Number of authors	Public interest	+	Public interest increases funding
Number of authors	Current descriptions	+	Current descriptions are the number of species known by humankind, and high totals offer work for many experts
Description curve residuals	Body size	–	Large species need more time for description, and might result in more irregular description frequencies
Description curve residuals	Number of authors	–	Many authors lead to less variation, as individual authors have less impact on overall description rates
Description curve residuals	Europe, USA, Canada, Russia	–	Easy access to sampling regions
Description curve residuals	Soil-dwelling/endoparasitic	+	Difficult access makes campaigns at specified times more likely than constant discovery
Description curve residuals	Aquatic	–	Constant access to samples through commercial fishing
Initial description time	Body size	–	Large species are discovered easier in general
Initial description time	Number of authors	–	Many authors lead to higher yearly description numbers
Initial description time	Europe, USA, Canada, Russia	–	Easy access to sampling regions
Initial description time	Soil-dwelling/endoparasitic	+	Soil-dwelling/endoparasitic species are hard to detect
Initial description time	Aquatic	–	Species known through fishing pre-date Linné’s taxonomic classification and may make up to 10%
Initial description time	Description curve residuals	+	High variability will rather affect beginning of time period, where species description was slower in general
Future descriptions ratio	Body size	–	Large species will mostly have been described already
Future descriptions ratio	Number of authors	–	Many authors might already have described most of the species
Future descriptions ratio	Europe, USA, Canada, Russia	–	Species outside Europe, the USA, Canada, and Russia are more likely to not be described yet
Future descriptions ratio	Soil-dwelling/endoparasitic	+	Soil-dwelling/endoparasitic species are likely still under-researched
Future descriptions ratio	Aquatic	+	Several habitats as deep sea and poles are difficult to sample
Future descriptions ratio	Description curve residuals	–	High variability may have rather slowed down earlier descriptions

Hypotheses and explanations of relationships used to construct the structural equation model linking predictors and parameters of estimated cumulative description curves.

## Results

### Data collection

We included 47 taxonomic groups in our study (Fig. 1). Of these, eight groups were related, but paraphyletic assemblages of mostly aquatic small plants and animals, among them Amoebozoa, Foraminifera, and Ciliophora. Five groups comprised the plants *sensu lato*, i.e., red algae (Rhodophyta), green algae (Chlorophyta), hornworts and liverworts (Marchantiophyta), true mosses (Bryophyta), and vascular plants (Tracheophyta). Four groups were fungi, including the well-known Ascomycota and Basidiomycota. The following were jellyfish *sensu lato* (Cnidaria), sponges (Porifera), nematodes (Nematoda), tardigrades (Tardigrada) and several groups of arthropods, with insects distinguished at the order level. A sister group to the aforementioned, Spiralia, i.e. animals with a spiral cell division pattern in embryonic stage, included flatworms (Platyhelminthes) and molluscs (Mollusca). Finally, there were the chordates (Chordata), including all vertebrates.

The number of current descriptions and mean author numbers per year differed markedly across groups (Table 2, Suppl. Fig. 2). Most descriptions belonged to Coleoptera (421k), Tracheophyta (354k), and Lepidoptera (148k). Fewest descriptions were found in Haptophyta (432), a group of unicellular algae, Gastrotricha (831), the hairybellies, and Chytridiomycota (977), the chytrids. Mean author numbers per year also varied considerably, with most (285) in Tracheophyta, followed by Coleoptera (135), and fewer than one author per year on average in Haptophyta and Gastrotricha.

**Table 2:** Description curve predictors.

Group	Current descriptions	Number of authors	Public interest	Body size	Soil-dwelling/endoparasitic	Aquatic	Europe, the USA, Canada, and Russia
Tracheophyta	354,344	285.3	341,495	2	1	1	0.37
Marchantiophyta	7208	5.6	32	0	1	1	0.33
Bryophyta	11,049	11.6	4507	1	1	1	0.40
Chlorophyta	5512	7.6	92	−1	1	5	0.83
Rhodophyta	7197	8.3	122	2	1	5	0.71
Ochrophyta	31,162	24.5	25	−2	1	5	0.70
Oomycota	1670	3.1	0	0	3	2	0.78
Foraminifera	30,026	15.5	0	−1	3	5	0.69
Ciliophora	8582	7.8	4	−1	3	3	0.75
Myzozoa	9014	15.1	1	−2	4	2	0.81
Haptophyta	432	0.8	2	−3	1	5	0.85
Euglenozoa	2521	3.9	0	−2	1	5	0.77
Amoebozoa	2936	5.1	0	−1	3	3	0.89
Chordata	74,981	84.3	434,058	2	1	2	0.39
Platyhelminthes	20,258	22.8	639	0	4	2	0.50
Gastrotricha	831	0.9	0	0	1	5	0.76
Ectoprocta	5870	3.2	1	−1	1	5	0.61
Mollusca	98,947	64.4	8855	1	1	4	0.53
Nemertea	1349	1.3	0	1	4	5	0.58
Annelida	22,326	21.1	1597	1	2	4	0.48
Lepidoptera	148,522	90.3	28,026	1	1	1	0.30
Trichoptera	16,976	7.7	0	1	1	1	0.42
Diptera	219,245	81.2	7816	0	1	1	0.46
Siphonaptera	2621	3.2	1473	0	1	1	0.46
Coleoptera	421,378	135.6	3121	0	1	1	0.42
Neuroptera	6847	4.2	1	1	1	1	0.31
Hymenoptera	172,007	64.1	11,181	0	1	1	0.48
Hemiptera	116,521	57.1	432	1	1	1	0.40
Psocodea	11,612	5.8	201	0	1	1	0.40
Thysanoptera	6826	4.2	15	−1	1	1	0.42
Plecoptera	4140	3.9	1	1	1	5	0.48
Dermaptera	2226	1.4	163	1	1	1	0.25
Dictyoptera	11,168	7.0	504	1	1	1	0.50
Orthoptera	32,041	16.4	14,742	1	1	1	0.26
Ephemeroptera	5056	5.1	37	1	1	5	0.43
Odonata	6869	5.8	1302	1	1	1	0.26
Crustacea	72,451	60.3	3261	1	1	5	0.44
Myriapoda	18,678	7.4	433	1	1	1	0.49
Arachnida	138,514	82.3	7328	1	1	1	0.39
Tardigrada	1215	1.9	5	−1	2	3	0.55
Nematoda	29,527	32.1	28	0	4	3	0.55
Cnidaria	12,785	12.0	620	1	1	5	0.51
Porifera	8724	5.0	2487	1	1	5	0.44
Basidiomycota	48,428	41.3	2203	1	5	1	0.62
Ascomycota	87,760	76.2	3251	1	5	1	0.63
Chytridiomycota	977	1.5	0	−2	4	3	0.78
Microsporidia	1155	2.3	0	−3	5	3	0.77

Current descriptions and the number of authors were obtained from LifeGate. Public interest data was collected from BiL Explorer. Data on body size data, soil-dwelling/endoparasitic species, and aquatic habitat use from different internet sources (see Suppl. Table 1). The fraction of species occurring in Europe, the USA, Canada, and Russia from GBIF. Public interest gives the total number of occurrences of names from the respective group in the literature corpus, body size gives the average body size of the respective group as an exponent of mm to base 10. Soil-dwelling/endoparasitic and aquatic give the fraction of such species in the respective group on a scale from 1–5 (see Methods for details).

**Fig. 2:**
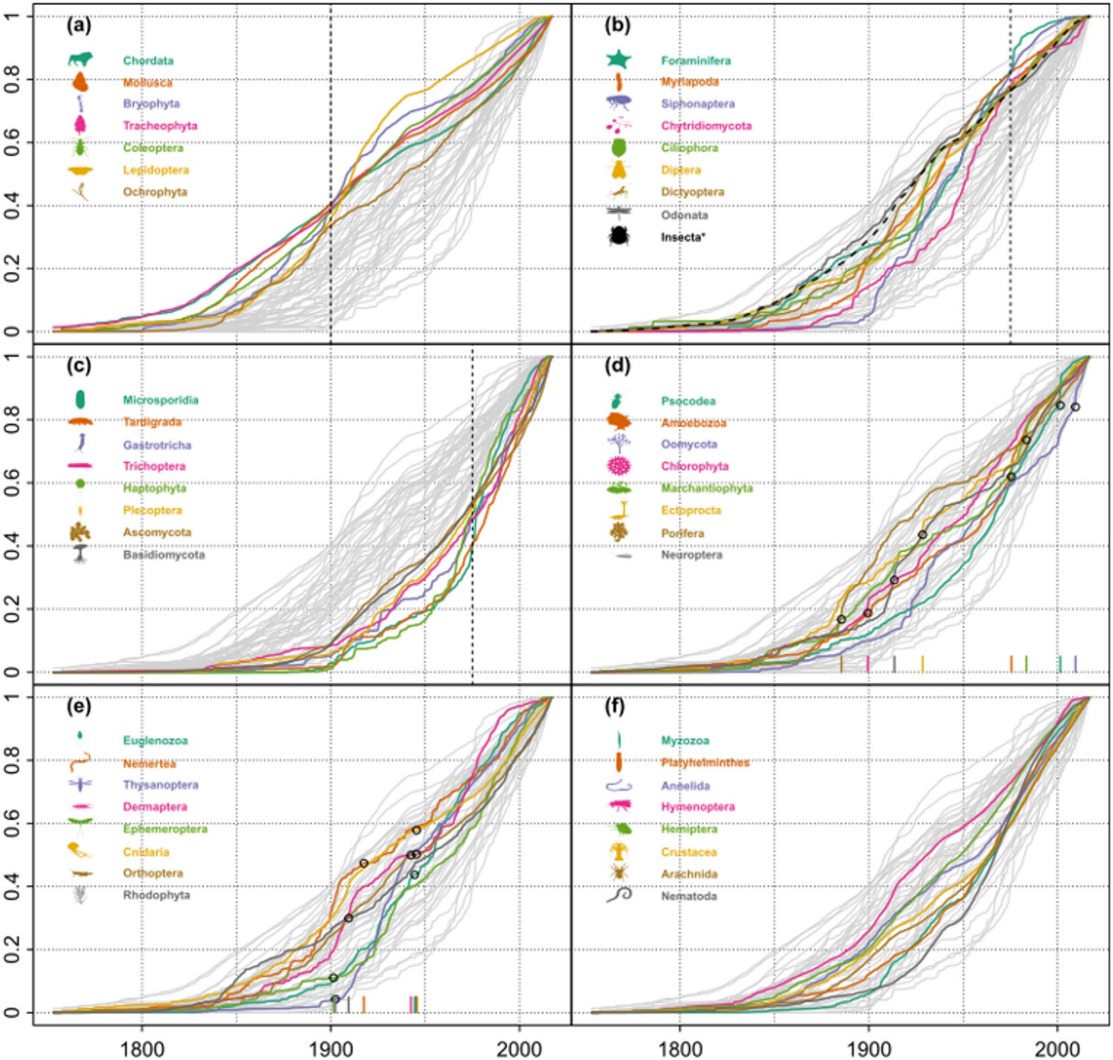
Cumulative description curves. All curves were scaled to [0,1] to allow for a comparison of highly different species numbers. Curves start in 1753, the year of the publication of Linné’s *Species Plantarum*, and end in 2017. Groups are distributed across panels according to some basic characteristics: (**a**) the best-described groups in 1900 compared to their current total; (**b**) the best-described groups in 1975 compared to their current total; (**c**) the least-described groups in 1975 compared to their current total; (**d**) groups with a period of strongly increased yearly description rate; (**e**) groups with a period of strongly decreased yearly description rate; (**f**) remaining groups. Vertical dashed lines in panels a to c show the years 1900, 1975, and 1975, respectively. Circles and small vertical bars in panels d and e show periods of increased and decreased description activity, respectively. Groups fulfilling several criteria are only highlighted in the first respective panel. Note that insects are shown as separate orders and aggregated into the class Insecta (dashed line in panel **b**).

Cumulative description curves expressed marked variation in their overall shape (Fig. 2, see Suppl. Fig. 3 for descriptions per year), and are ordered according to relative over- or under-sampling in 1900 and 1975, respectively, as well as intervals of exceptionally high and low description rates. Examples for relative over-sampling around 1900 include Tracheophyta and Chordata (Fig. 2a), for relative over-sampling around 1975 Foraminifera and Myriapoda (Fig. 2b), and Microsporidia, Tardigrada, and Gastrotricha for relative under-sampling before 1975 (Fig. 2c). Marchantiophyta and Porifera exemplified periods of strongly increased yearly description rates (Fig. 2d), Cnidaria and Orthoptera, on the contrary, had exceptional low-description periods (Fig. 2e). The remaining groups showed average cumulative description curves (Fig. 2f).

Public interest, measured as occurrence in literature, was highest for Chordata (434k), followed by Tracheophyta (341k) (Table 2). Other groups were considerably less mentioned, with only Lepidoptera, Hymenoptera, and Orthoptera surpassing 10k, and several groups were never mentioned in the literature evaluated.

The largest average body size in the range of multiples of 10 cm (10^2^mm) was found in Tracheophyta, Rhodophyta, and Chordata. Smallest average body size was observed in Haptophyta and Microsporidia, in the range of multiples of 1 μm (10^− 3^mm). Fractions of soil-dwelling/endoparasitic species were high in fungi, Myzozoa, Platyhelminthes, Nemertea, and Nematoda. Classifying the number of aquatic species within groups, we found 25 and 16 groups to be mostly terrestrial or aquatic, respectively. Only six groups had comparable numbers of species in and outside water: Tardigrada, Ciliophora, Amoebozoa, Chytridiomycota, Nematoda, and Microsporidia. The fraction of worldwide occurrences found in Europe, the USA, Canada, and Russia from Dermaptera, Dictyoptera, Orthoptera, and Odonata, of which less than 1/4 of the species was found in these regions, to Chlorophyta, Euglenozoa, Oomycota, Chytridiomycota, and Microsporidia, with about 80% of species with occurrences registered in GBIF having at least one occurrence from Europe, the USA, Canada, and Russia.

### Description rate anomalies

**Fig. 3:**
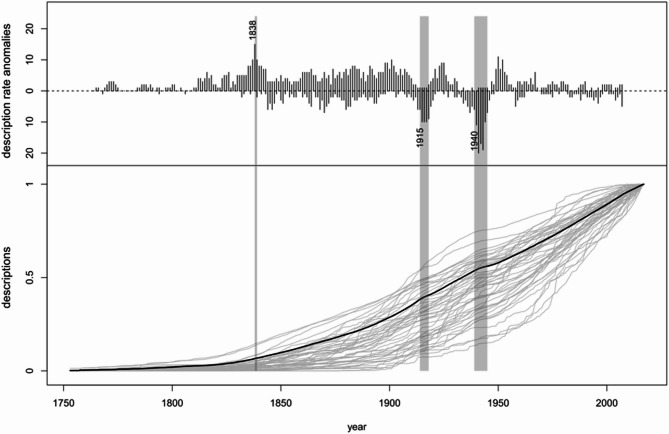
Description rate anomalies. The upper panel shows the number of taxonomic groups (out of the total of 47) with deviations of > 50% from yearly description rate within five-year-intervals based on the averages of the preceding five-year-intervals, accounting for re-bounces from former deviations. The lower panel shows cumulative description curves for all taxonomic groups in grey and the averaged description curve for all groups in black. Numbers indicate years with exceptionally high yearly description rate anomalies. The blueish shaded area marks the publication of the “Illustrations of the Zoology of South Africa”^27^ and the start of monthly publications of the “Annals of Natural History”^28^. Reddish shaded areas mark the First World War (1914–1918), and the Second World War (1939–1945).

We discovered several abrupt changes in yearly description rates affecting many taxonomic groups simultaneously revealing correlations with historical events (Fig. 3). A weak sinusoidal fluctuation in description rate anomalies persisted throughout the entire study period, reflecting the moving window methodology: rate changes by external events lead to positive or negative fluctuations, and their opposite, when rates go back to normal. A notable positive peak in 1838 coincided with the publication of Illustrations of the Zoology of South Africa^27^, as well as the monthly publication of the Annals of Natural History^28^, the predecessor of the Journal of Natural History still published today. Negative throughs occurred during major conflicts: World War I (1914–1918), and World War II (1939–1945).

### Prediction of cumulative description curves

After fitting half normal distributions to the cumulative description data, we extracted the ratio between estimated future descriptions and current descriptions (future descriptions ratio), the time until 10% of the current descriptions were reached (initial description time), and description curve residuals (Table 3, see Suppl. Figure 4 for curves). Future descriptions ratios ranged from 1.00 (indicating < 1% expected increase in total descriptions, i.e. species numbers) for twelve groups to > 2.00 (projecting > 100% increase) for Psocodea, Gastrotricha, Tardigrada, and Trichoptera. A short initial description time was observed for Chordata (60 years), Tracheophyta (64), Mollusca (70), Coleoptera (81), and Bryophyta (84). In contrast, groups with a large initial description time were Nematoda (161), Gastrotricha (161), Tardigrada (173), Microsporidia (181), and Haptophyta (183). For Haptophyta, 10% of current descriptions were achieved only by the mid-20th century, compared to pre-1850 completion for other groups.

**Table 3:** Description curve parameters.

Group	Future descriptions ratio	Initial description time	Description curve residuals
Tracheophyta	1.00	64	0.00039
Marchantiophyta	1.29	109	0.00095
Bryophyta	1.00	84	0.00210
Chlorophyta	1.07	117	0.00014
Rhodophyta	1.36	98	0.00059
Ochrophyta	1.01	96	0.00097
Oomycota	1.01	139	0.00086
Foraminifera	1.09	113	0.00136
Ciliophora	1.00	113	0.00067
Myzozoa	1.08	151	0.00022
Haptophyta	1.01	183	0.00043
Euglenozoa	1.00	143	0.00010
Amoebozoa	1.50	122	0.00052
Chordata	1.01	60	0.00102
Platyhelminthes	1.23	142	0.00015
Gastrotricha	2.24	161	0.00024
Ectoprocta	1.07	100	0.00085
Mollusca	1.00	70	0.00127
Nemertea	1.01	98	0.00126
Annelida	1.19	114	0.00054
Lepidoptera	1.02	87	0.00221
Trichoptera	5.86	150	0.00016
Diptera	1.05	109	0.00027
Siphonaptera	1.05	138	0.00083
Coleoptera	1.00	81	0.00056
Neuroptera	1.05	114	0.00107
Hymenoptera	1.03	101	0.00050
Hemiptera	1.12	115	0.00011
Psocodea	2.02	141	0.00023
Thysanoptera	1.00	144	0.00155
Plecoptera	1.63	153	0.00013
Dermaptera	1.06	117	0.00109
Dictyoptera	1.00	108	0.00065
Orthoptera	1.01	110	0.00071
Ephemeroptera	1.36	138	0.00039
Odonata	1.00	92	0.00054
Crustacea	1.64	121	0.00026
Myriapoda	1.00	121	0.00051
Arachnida	1.43	130	0.00023
Tardigrada	3.37	173	0.00023
Nematoda	1.07	161	0.00029
Cnidaria	1.01	91	0.00114
Porifera	1.00	98	0.00284
Basidiomycota	1.29	145	0.00021
Ascomycota	1.26	141	0.00045
Chytridiomycota	1.00	143	0.00052
Microsporidia	1.27	181	0.00102

Curve parameters were analytically derived from the calculated approximations of the cumulative descriptions. Future descriptions ratios are the ration of future expected to current descriptions. Initial description time shows the number of years since 1753 it took for the cumulative descriptions to reach 10% of the current values. Description curve residuals are the sum of squares calculated between the observed number of descriptions and the approximation curve values.

**Fig. 4:**
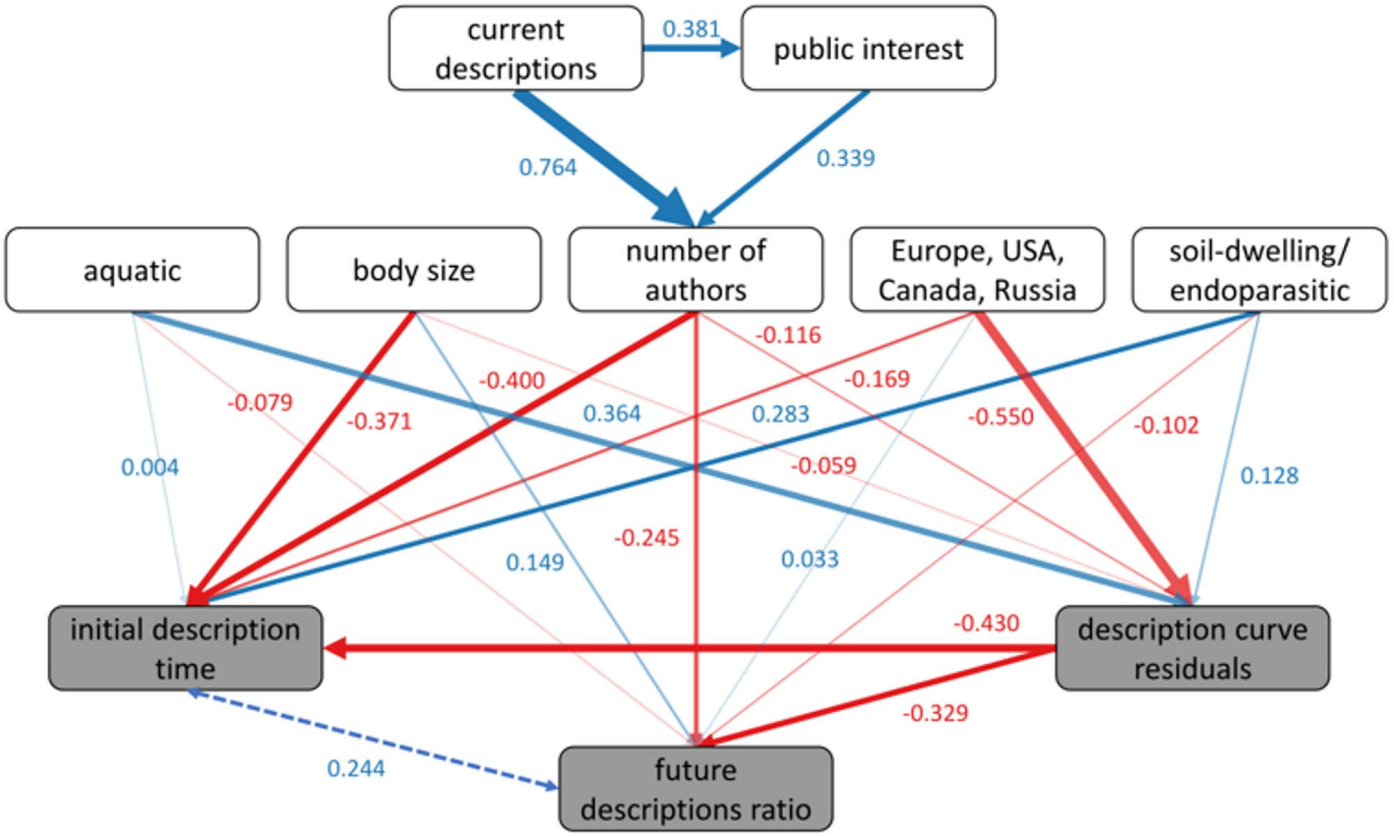
Structural equation model of description curve parameters and predictors. Predictors and curve parameters have white and grey backgrounds, respectively. Arrow colours indicate positive (blue) and negative (red) relationships (see also Suppl. Table 3). Coefficient values are given next to arrows, and arrow width is proportional to the latter. Transparency represents the absolute ratio between the standard deviation and the estimate of the coefficients between variables, i.e. |SD/Estimate|, with no transparency for values < 0.25, 25% transparency for values < 0.5, 50% transparency for values < 0.75, and 75% transparency for values ≥ 0.75. The overall posterior predictive p value (PPP) of the model was 0.489, indicating very good model fit. The dashed line shows the covariance between two curve parameters.

The Bayesian structural equation model demonstrated very good convergence (well-mixed traceplots) and fit (posterior predictive p value = 0.489). Most of our hypotheses were confirmed by the model (Table 1; Fig. 4, see Suppl. Table 3 for full diagnostics). Running the same analysis aggregating insects into the class Insecta to make the taxonomic levels used more consistent resulted in highly similar results (Suppl. Figure 5 and Suppl. Table 4). We found a strong link between public interest and current description numbers, i.e. the number of species known to date in a group. As expected, both current descriptions and public interest drove author numbers, although the influence of current descriptions was larger than that of public interest. Describing the cumulative description curve parameters, we found a negative, albeit weak, effect of author numbers on description curve residuals. The strongest determinant of description curve residuals, however, was occurrence in Europe, the USA, Canada, and Russia, with high occurrence values leading to small description curve residuals. We also found a strong positive relationship of the fraction of aquatic species with description curve residuals, contrary to our expectation. In line with our predictions, body size and soil-dwelling/endoparasitic species were negatively and positively related to description curve residuals, respectively, although their effects were weak.

Initial description time was mainly determined by body size, author numbers, and description curve residuals. All three affected initial description time negatively, which, in the cases of body size and author numbers, was in line with our expectation. Occurrence in Europe, the USA, Canada, and Russia, and the fractions of soil-dwelling/endoparasitic and of aquatic species had much less of an impact, with a positive one for soil-dwelling/endoparasitic species, and a negative of the other two.

The main predictors of future descriptions ratios were author numbers and description curve residuals, both having a negative impact. Other predictors had much less influence, and they were all negative, except body size. The fraction of aquatic species was negatively related to future descriptions ratios, in contrast to our expectation, but the relationship was very weak. We found a covariance of 0.244 between initial description time and future descriptions ratios.

## Discussion

This study provides the first overview of description rates and the resulting cumulative descriptions across all major groups of eukaryotic life on Earth. Curves fitted to the cumulative descriptions demonstrated the influence of biological and societal factors. Simultaneous changes in yearly description rates across groups likely reflect the impact of historical events.

In contrast to previous studies that developed partly mechanistic models^6,7^, using species-area relationships^9^ or taxonomic hierarchies^8^ to explain biodiversity, our approach focused on relating the shape of cumulative description curves to informative predictors in order to understand factors influencing biodiversity discovery. While individual researchers influence single or multiple descriptions, description curves for larger taxa are driven by more general factors operating at a level above individual contributions.

The LifeGate data used here aligned well with the most recent global databases on numbers of tracheophyte species^29–32^. A comparison with GBIF^18^ and Catalogue of Life^17^, two online sources with a broad taxonomic scope encompassing animals, fungi, and unicellular species, showed high concordance (Suppl. Figure 6). Larger discrepancies in certain groups (e.g., Tracheophyta, Coleoptera, Mollusca), are likely to be resolved in the future through improved handling of duplicate names by the databases. Specifically, the GBIF listing of > 500k accepted names for Tracheophyta is an artefact, given the consensus between LifeGate, Catalogue of Life, and global vascular plant checklists of around 350k species^33^.

Predictors of cumulative description curves were drawn from diverse sources, including Wikipedia, GBIF, and the BiL Explorer. Previous research demonstrated that the occurrence of biodiversity in literature as sourced by the BiL Explorer is a reliable measure of public awareness and valuation of nature^19,34,35^. We compared the BiL Explorer data with an alternative measure, the number of results obtained by entering the group names in Google Search, and found a good fit (Suppl. Figure 7).

The use of GBIF data to assess global taxonomic group distributions is subject to well-documented limitations^36^. Certain groups (e.g., birds) are strongly overrepresented, while others (e.g., insects) remain underrepresented, both when comparing occurrences and species numbers. Furthermore, a strong geographic bias towards developed nations persists in occurrence data^37^, likely inflating European, US, and Canadian occurrence fractions for groups like Chlorophyta (Table 2). Nevertheless, for analyses of broad taxonomic scope, GBIF remains the most comprehensive resource at the global scale, as evidenced by its widespread use in comparative biogeographic research^38,39^.

Body size, the fraction of soil-dwelling/endoparasitic species, and the fraction of aquatic species were recorded using coarse ordinal scales to reflect uncertainties of phylum-level data aggregation. Specialised databases provide precise trait data for specific groups (e.g., plants and birds^40,41^, but these cover only a fraction of the taxonomic breadth addressed here. Our approach represents the most feasible method for cross-taxon comparisons given current data limitations.

Considering the description curve anomalies, systematically linking historic events and publications to simultaneous surges of taxonomic descriptions in several groups is difficult, as information on the outlets of first descriptions is not standardized for many groups, and a range of historic events may have driven increased description pace. We just point out three notable extremes: The year 1838, one of the positive extremes in description rate anomalies (Fig. 3), coincides with the maximum of the use of biodiversity-related vocabulary in Western creative literature between the 18th and 20th century^19^, hinting to the role of public interest on professional biodiversity exploration, e.g., through funding or public recognition. For year-long negative troughs during great crises of humanity, as the world wars, there is little doubt that the diversion of resources from science and possibly the deployment and death of taxonomists at the frontlines in these periods were the factors ultimately driving decreased species discovery and description rates^42,43^.

Besides these unique events, that cannot be accounted for in predictions^44^, our results support the notion of an interplay of both biological and societal factors influencing the rate of descriptions, mirroring findings on the level of individual species^45^.

Public interest has been shown to determine the number of authors, i.e., taxonomists doing the actual description work^36^. Author numbers are positively related to description numbers, and therefore included in many models explaining past discovery rates. They have also been used in models estimating true diversity using species description curves^6^, but as future author numbers are unknown, their usefulness in mechanistic models extrapolating species discovery rates is limited.

Arguably the most prominent biological property, body size turned out to be a key predictor, with larger-bodied groups having a shorter initial description time, a pattern consistent with prior findings^7,45^. We observed a positive relationship of body size with future descriptions ratios. This is in contrast with theoretical expectations and empirical evidence that smaller species will represent the bulk of future discoveries due to ecosystem carrying capacity constraints^43,45^, an expectation that arises from the fact that larger species tend to need more space to maintain viable populations, and are therefore less likely to have been overlooked in the past. The contrary pattern in our data appears to be driven by Trichoptera with a relatively large body size (an outlier, predicted to increase nearly six-fold with a future descriptions ratio of 5.86, compared to current species numbers, Suppl. Figure 7).

Species’ distribution patterns strongly influenced early discovery dynamics, reflecting a historical taxonomic focus on Europe, the USA, Canada, and Russia. Empirical data suggests that even now, species knowledge in Europe, the USA, Canada, and Russia is more complete than elsewhere^46–48^. The relatively easy and constant access to sampling material in Europe, the USA, Canada, and Russia enabled short initial description times and a continuous description of new species, evidenced by small description curve residuals, unlike in groups occurring mainly in other parts of the world, where species description was most likely related to occasional expeditions followed by periods with low description rates, and limited research funding as well as colonial suppression in developing countries.

Groups with many species living in hidden habitats, i.e. soil-dwelling and endoparasitic species, were linked to large initial description times, as they are harder to find than others or may have required the invention of specific tools enabling their detection^49,50^. Their link to large description curve residuals is likely due to a more difficult and campaign-driven access to their habitats. The negative relationship of the fraction of soil-dwelling/endoparasitic species on future descriptions ratios is driven by several groups with large future descriptions ratios not being soil-dwelling or endoparasitic at all (Suppl. Figure 7). Even as we expect new species to be discovered in the soil or as endoparasites, more are expected to be discovered elsewhere.

Of all predictors, the fraction of aquatic species turned out to be the weakest. This is surprising, given that sampling effort and species distributions differ markedly between terrestrial and aquatic environments. It has been estimated that about 21% of marine fish species are still to be described^46^, while a complete inventory of all marine life could take some centuries at current pace^43^. One main reason could be a relative scarcity of available resources causing only slowly added descriptions. The positive effect of aquatic species on description curve residuals may relate to sampling campaigns into the deep sea or remote parts of the ocean that lead to several new discoveries, interspersed with times with few new descriptions.

The future descriptions ratios translate to few estimated new descriptions for about half of the groups considered (≤ 5% increase compared to current numbers), moderate increases for 19 groups (≤ 50%), and large increases for the remaining seven taxonomic groups (> 50%). It is not the focus of this study to provide new estimates of global biodiversity, and it has been shown that best estimates changed dramatically within the last decades as new data became available^51^. Nevertheless, we will discuss some groups for illustrative purposes:

For Tracheophyta, estimates of total species numbers have increased in the past, from about 250k in the 90 s to 400k in 2001, to about 450k in 2015^52^. The latter two predictions did not materialise. Our prediction of a future descriptions ratio of 1.00 assumes few new descriptions to be expected (< 3500, given a current total of 350k), which is below the roughly 5000 new species names registered annually by IPNI^53^, although the latter do not necessarily represent actual species.

For Mollusca, there is disagreement even on the currently known diversity, estimates ranging between 34k and 120k, with Rosenberg^54^ giving around 76k in 2014, and LifeGate listing about 100k in 2017. The WoRMS database, an authoritative source on marine taxonomic groups (but also including non-marine species of those groups), gives roughly 90k species as the current species number of Mollusca^55^, close to the 100k from LifeGate and our future estimate, which, as with Tracheophyta, is no further increment (future descriptions ratio = 1.00).

The largest future descriptions ratio value recorded in our study is 5.86 for Trichoptera, a nearly six-fold increase. Ríos-Tuma et al.^56^ report that probably only 30% of the Andean Trichoptera are currently known, and about 50% within Ecuador. A study in West Java in Indonesia found about 80 operational taxonomic units, i.e. species, belonging to Trichoptera, of which only five could be identified to species and another four to genus level^57^. This suggests the potential for the number of species within this insect order to increase to the value estimated in our study.

By design, future descriptions ratios depend on the shapes of the cumulative description curves until the current date, and do not consider ecological and taxonomic insights that might warrant deviations from their current best-fit form. The shapes of the curves are partly driven by idiosyncratic changes in yearly description rates or data deficiency, i.e., issues with duplicate or unprecise species descriptions. The effort of cleaning up old unresolved descriptions and moving them to their correct place in the taxonomy is a daunting task. Botanists working on vascular plants (the group with the highest author/descriptions ratio) are in due course, but far from finished^33^. This work is less advanced in animals (M. Freiberg, pers. com.). This explains the large differences in terms of species numbers for the mollusc example above, and animal groups in general (see also differences between LifeGate, GBIF, and CoL in Suppl. Figure 6).

While our results appear to be robust, as demonstrated by the analysis using the aggregated insect orders producing qualitatively identical results, we want to point out that several subjective choices had to be taken during data compilation that might have impacted the results. For example, the assignment of biological properties was based partly on anecdotal descriptions of taxonomic groups and there is a range of other functions that could potentially be used to approximate cumulative descriptions than the one we chose. This adds to the uncertainty introduced through the unresolved and duplicated species descriptions mentioned above, correcting for which is a challenging task.

A solution for biodiversity estimation as well as targeted sampling guidance are dark diversity approaches identifying distributional gaps, i.e. regions with lower known diversity than other comparable ones^58,59^. Another way forward may be through simulations based on species properties and distributions. In conjunction with the framework presented here, they could be used to test how the drivers of description curves and the true unknown biodiversity interact, providing more likely estimates of Earth’s species richness in the future^60^.

## Supplementary Information

Below is the link to the electronic supplementary material.

Supplementary Material 1

Supplementary Material 2

Supplementary Material 3

## Data Availability

All data and code are archived and permanently accessible at Zenodo with DOI doi.org/10.5281/zenodo.17639993, which corresponds to the GitHub repository https://github.com/johnroxton/taxon-description-dates.
