# Supplementary figures and images for: Taxonomic description curves of major lineages are influenced by biological and societal factors
Source: Sci Rep. 2025 Nov 24;15:41837. doi: 10.1038/s41598-025-29845-y (PMC12647670; doi:10.1038/s41598-025-29845-y)

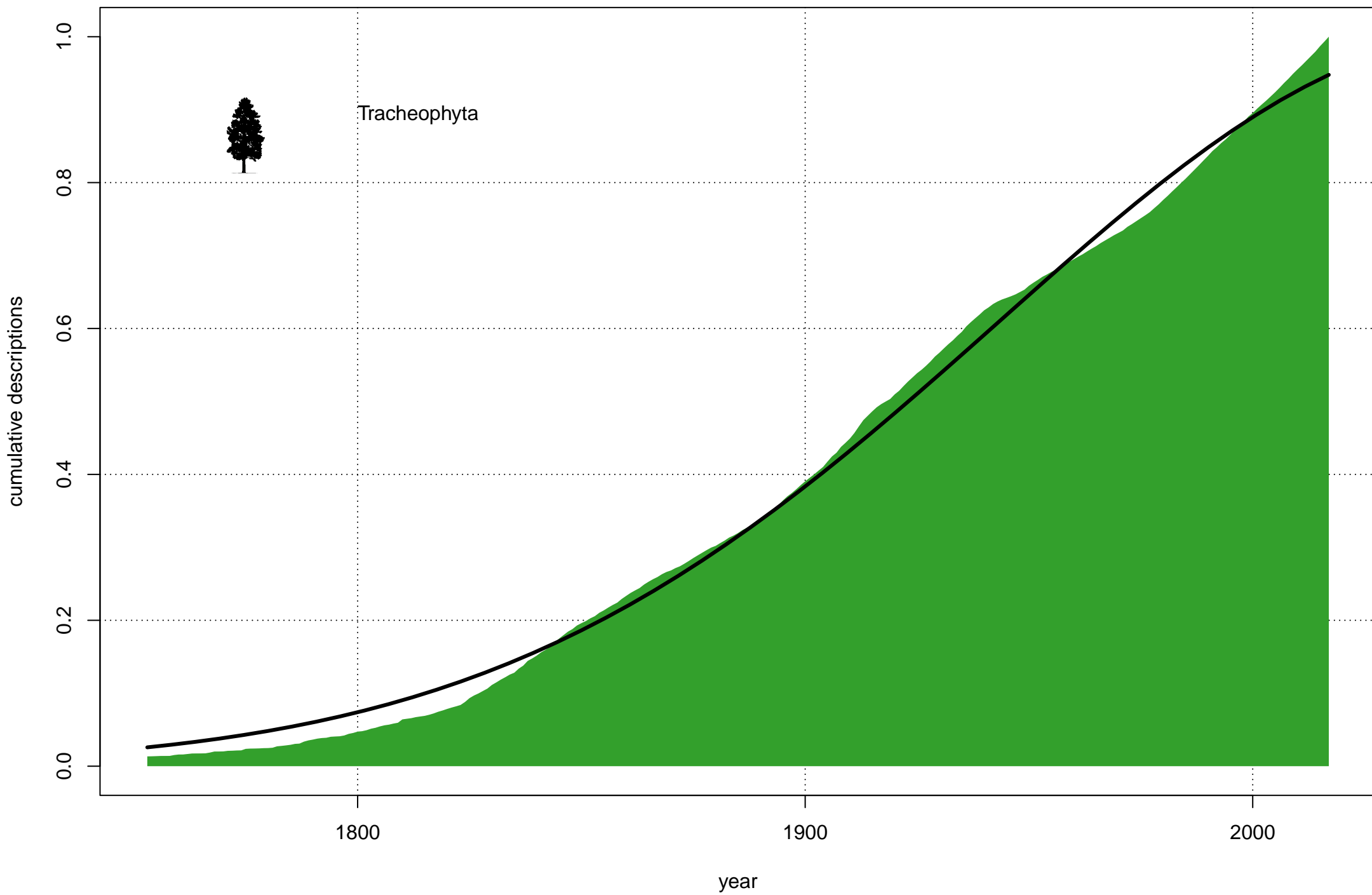

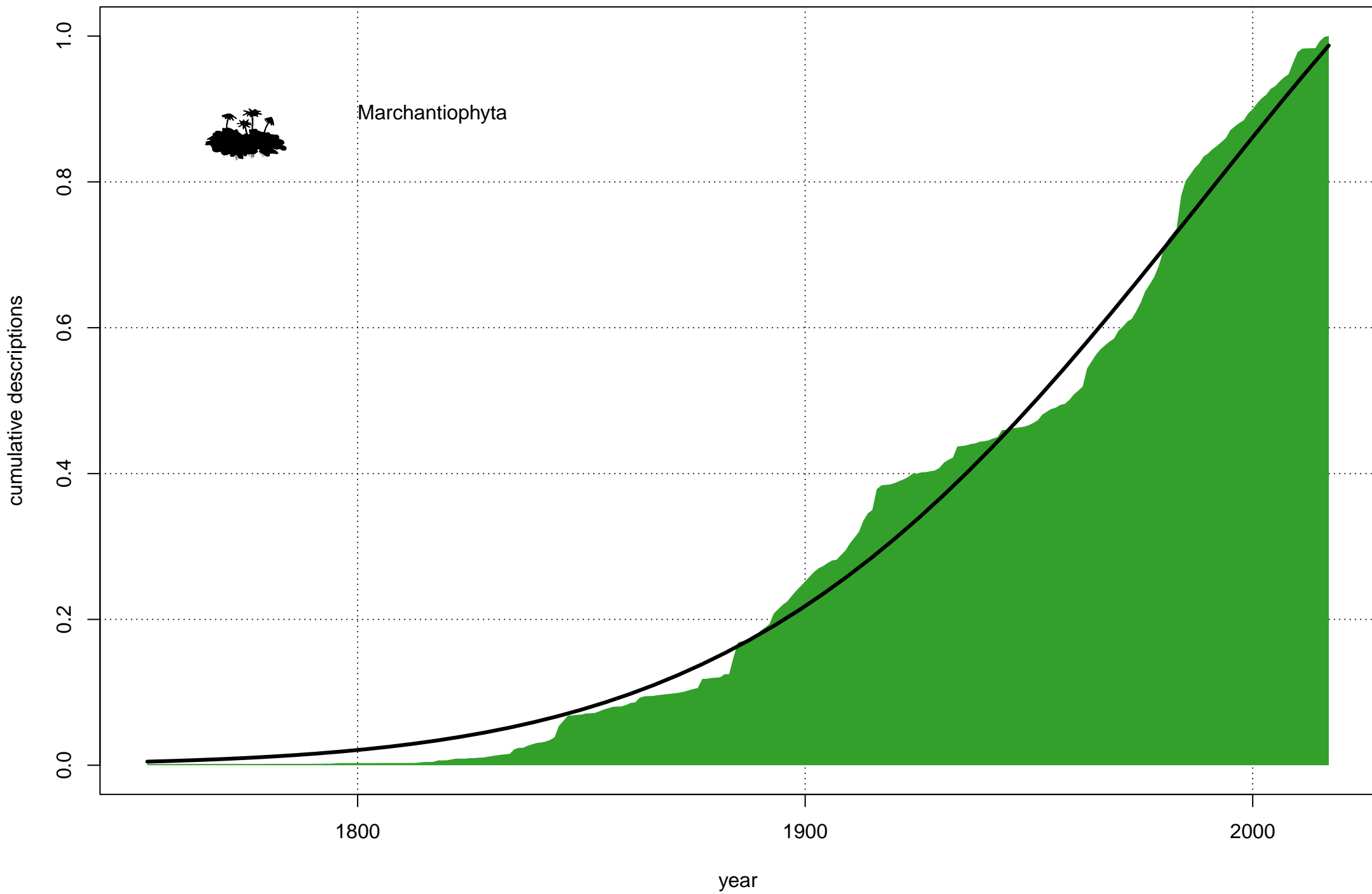

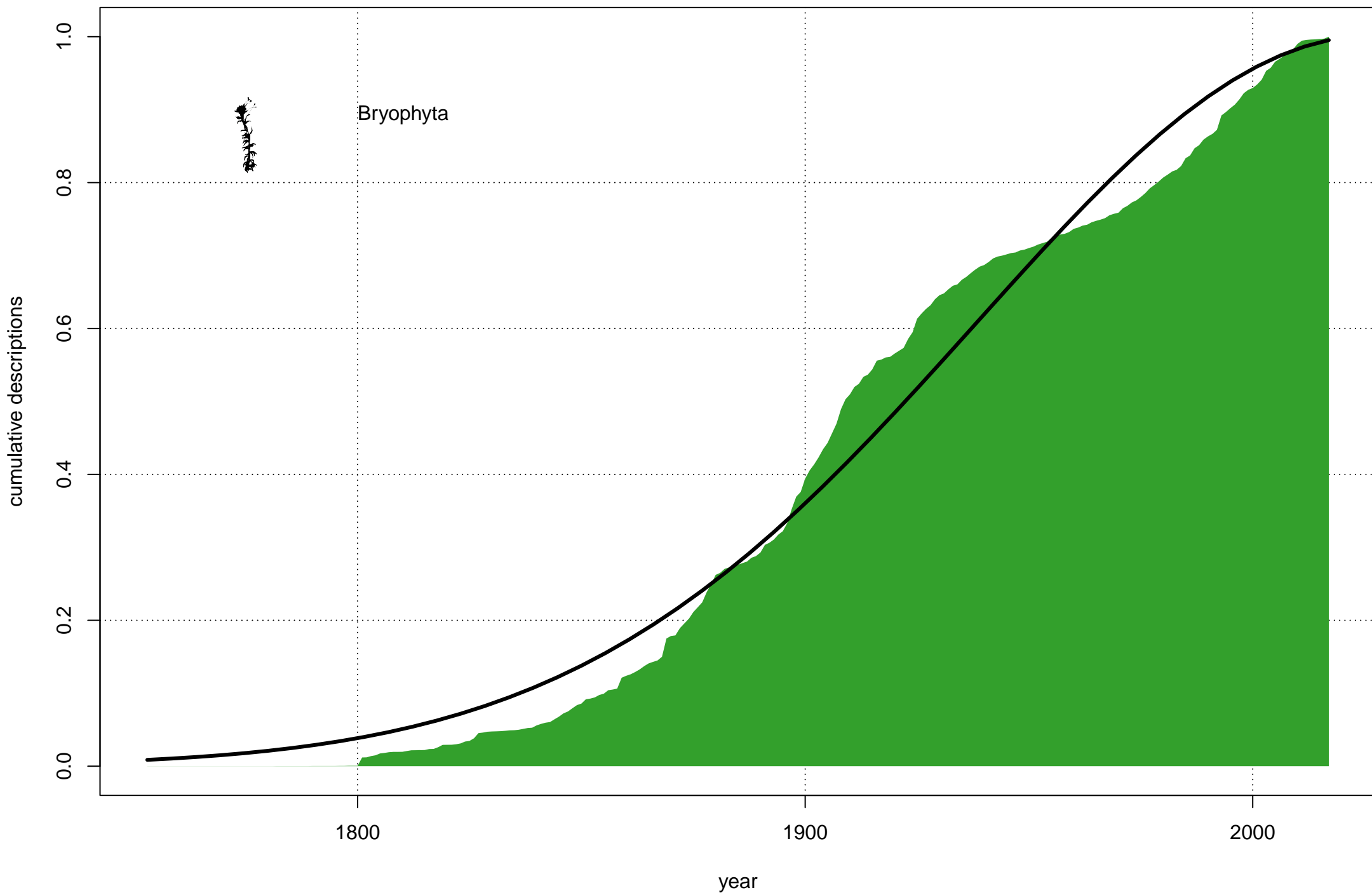

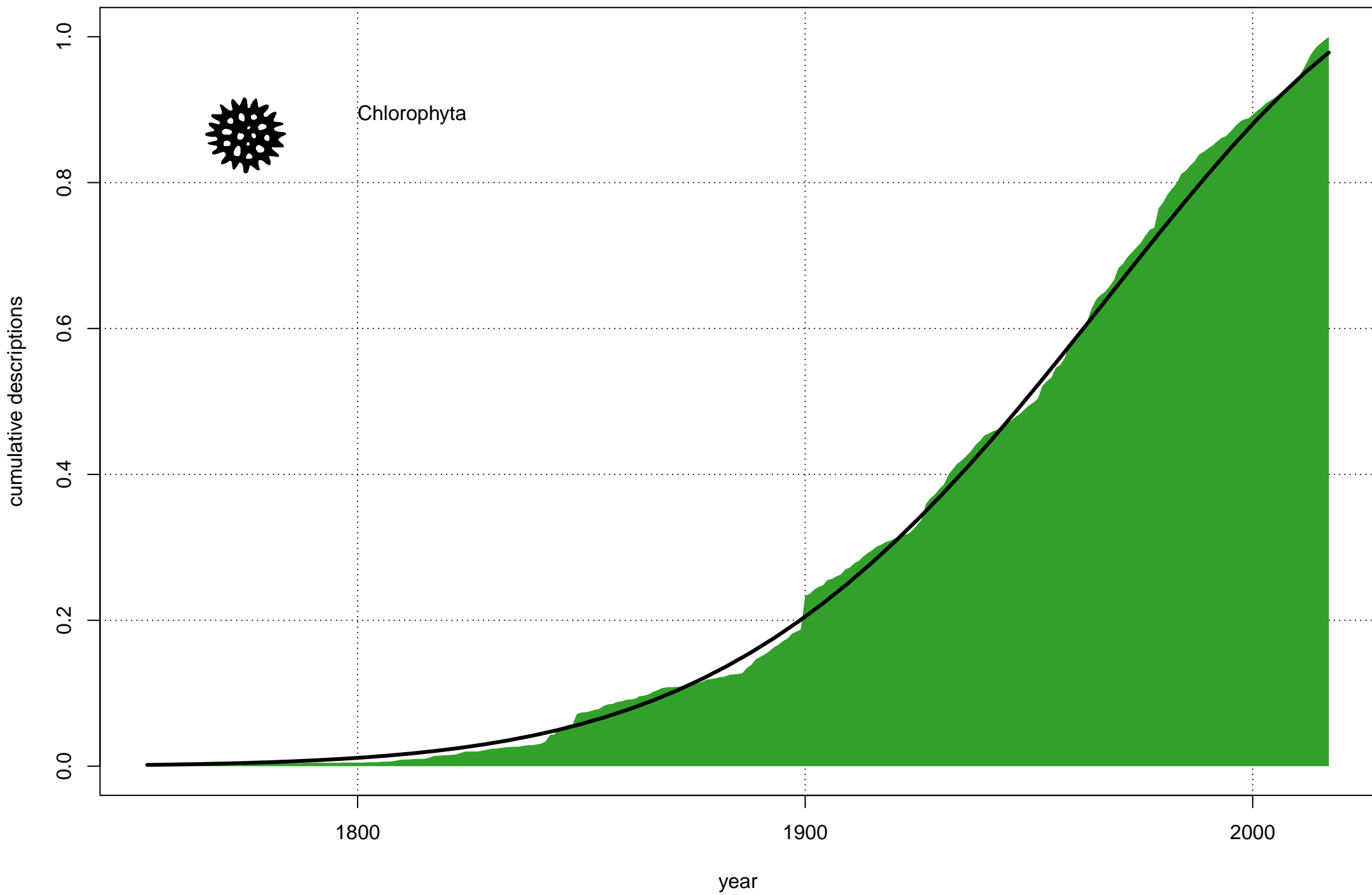

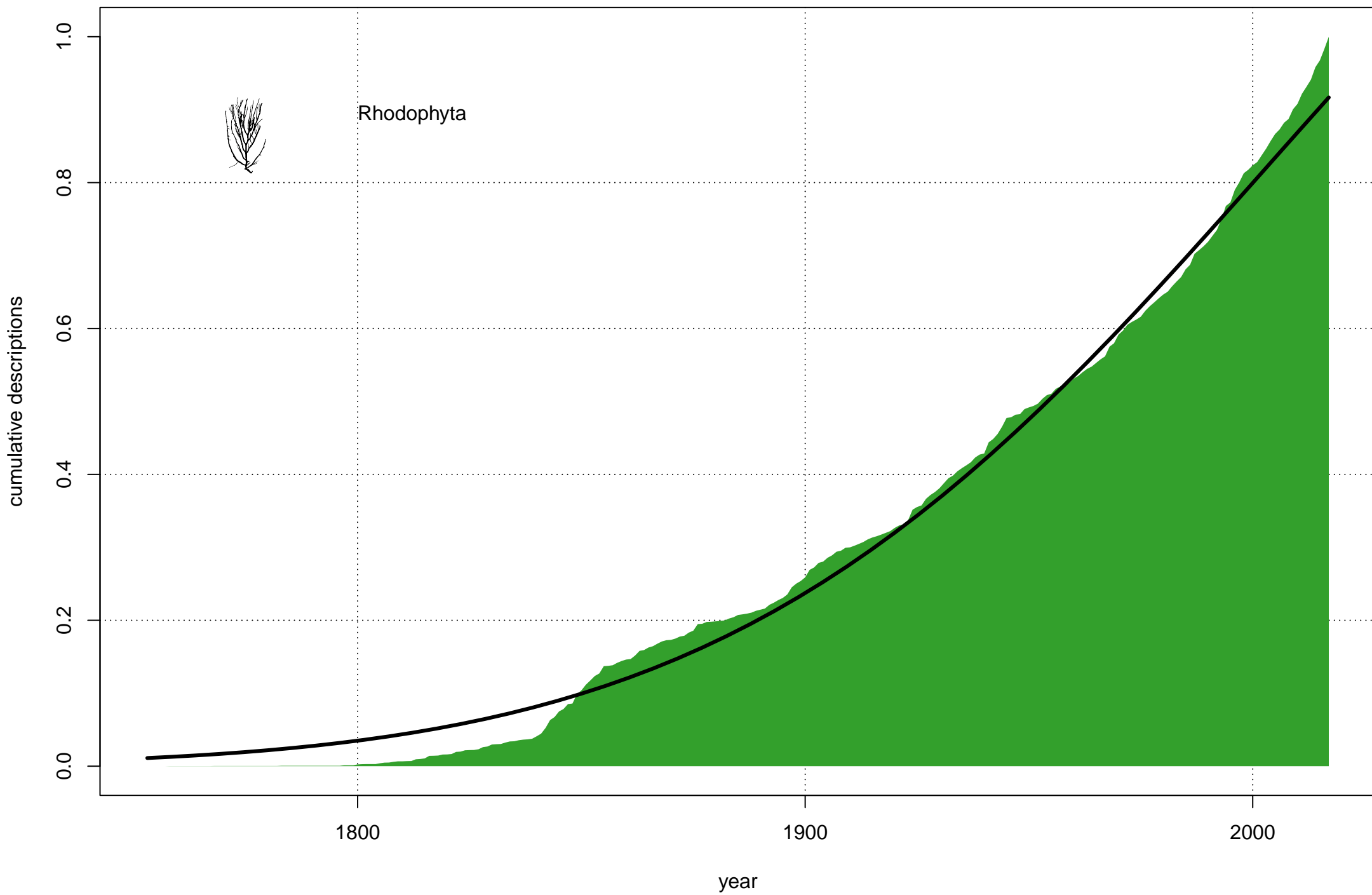

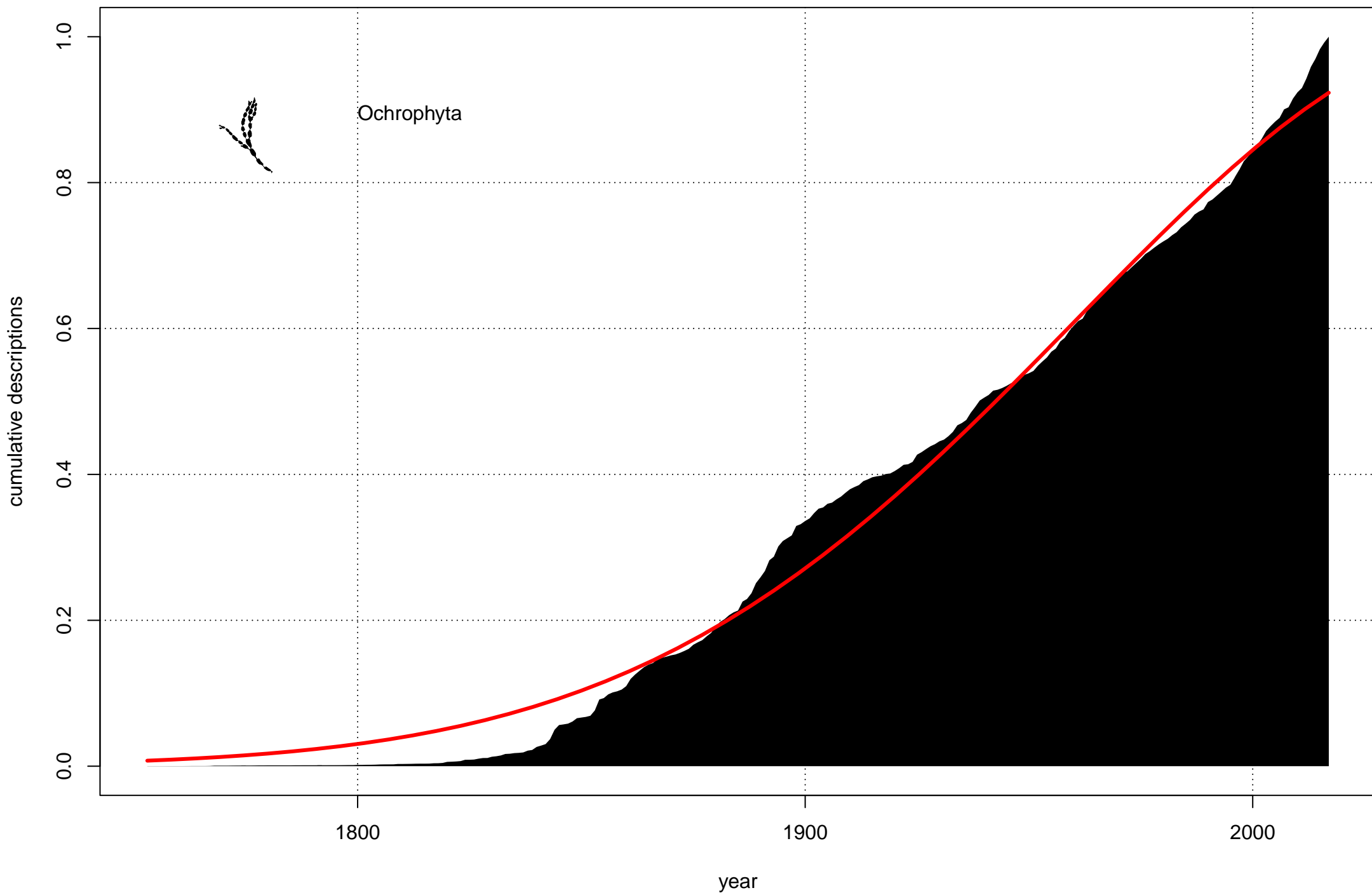

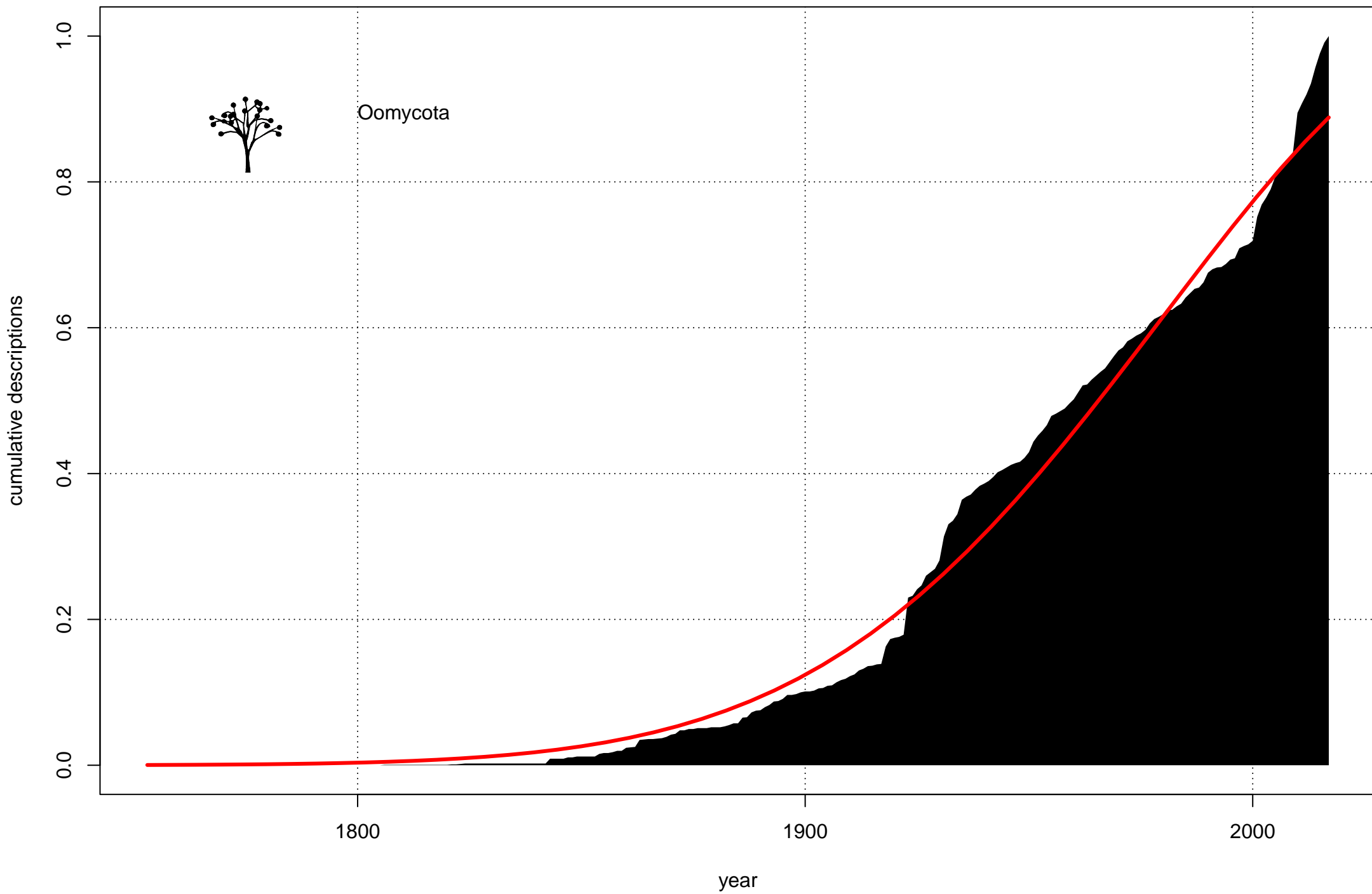

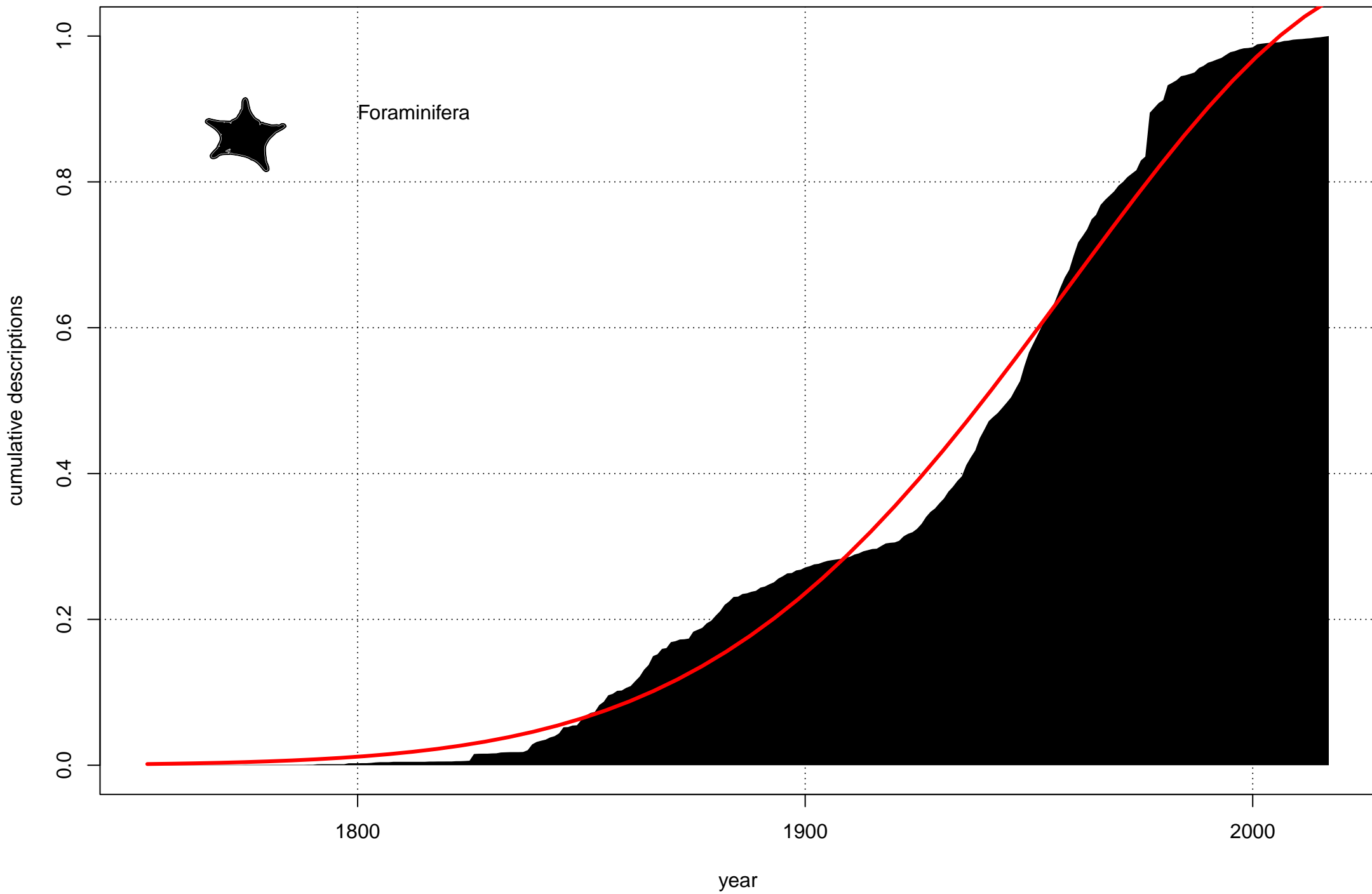

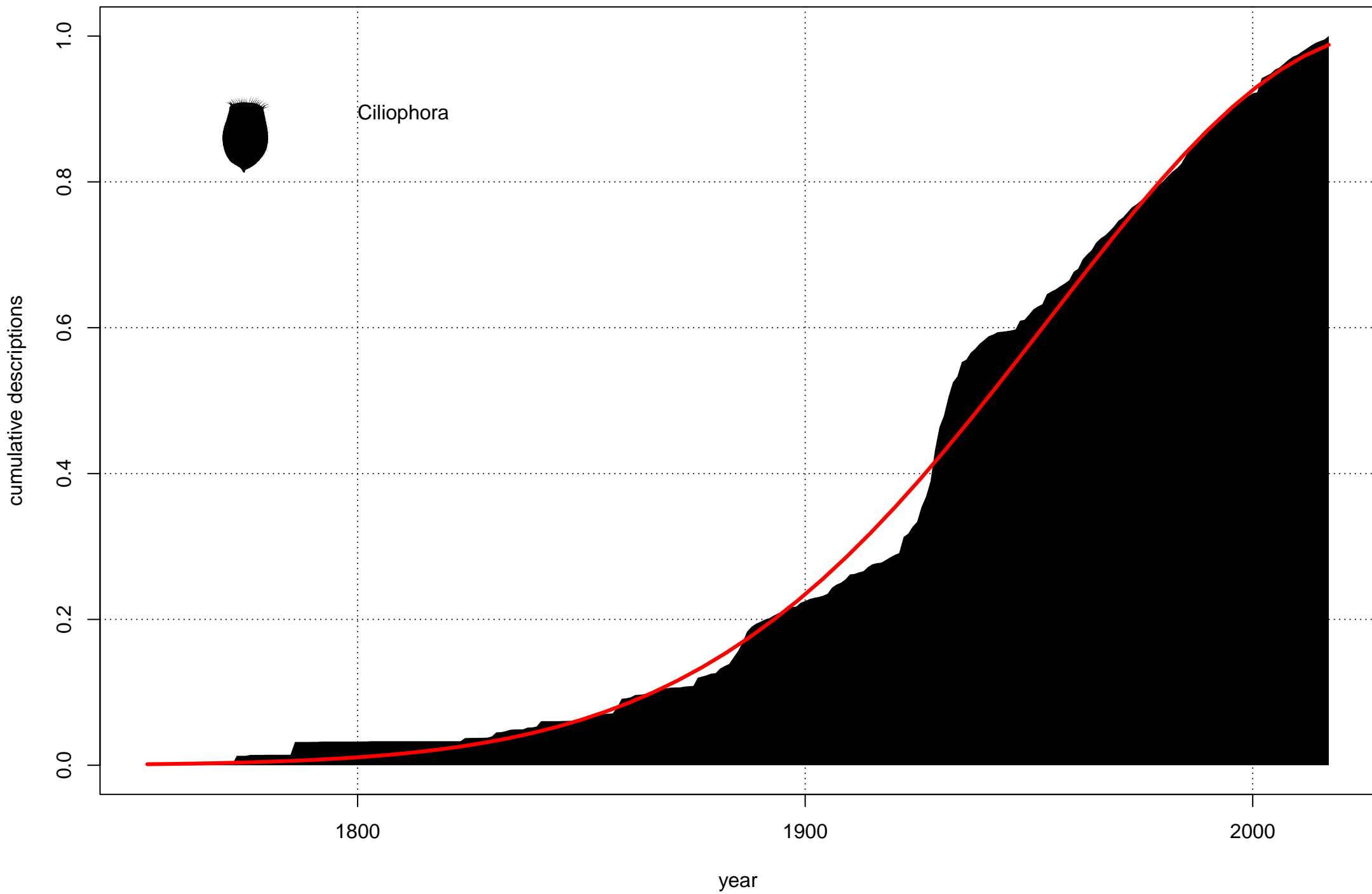

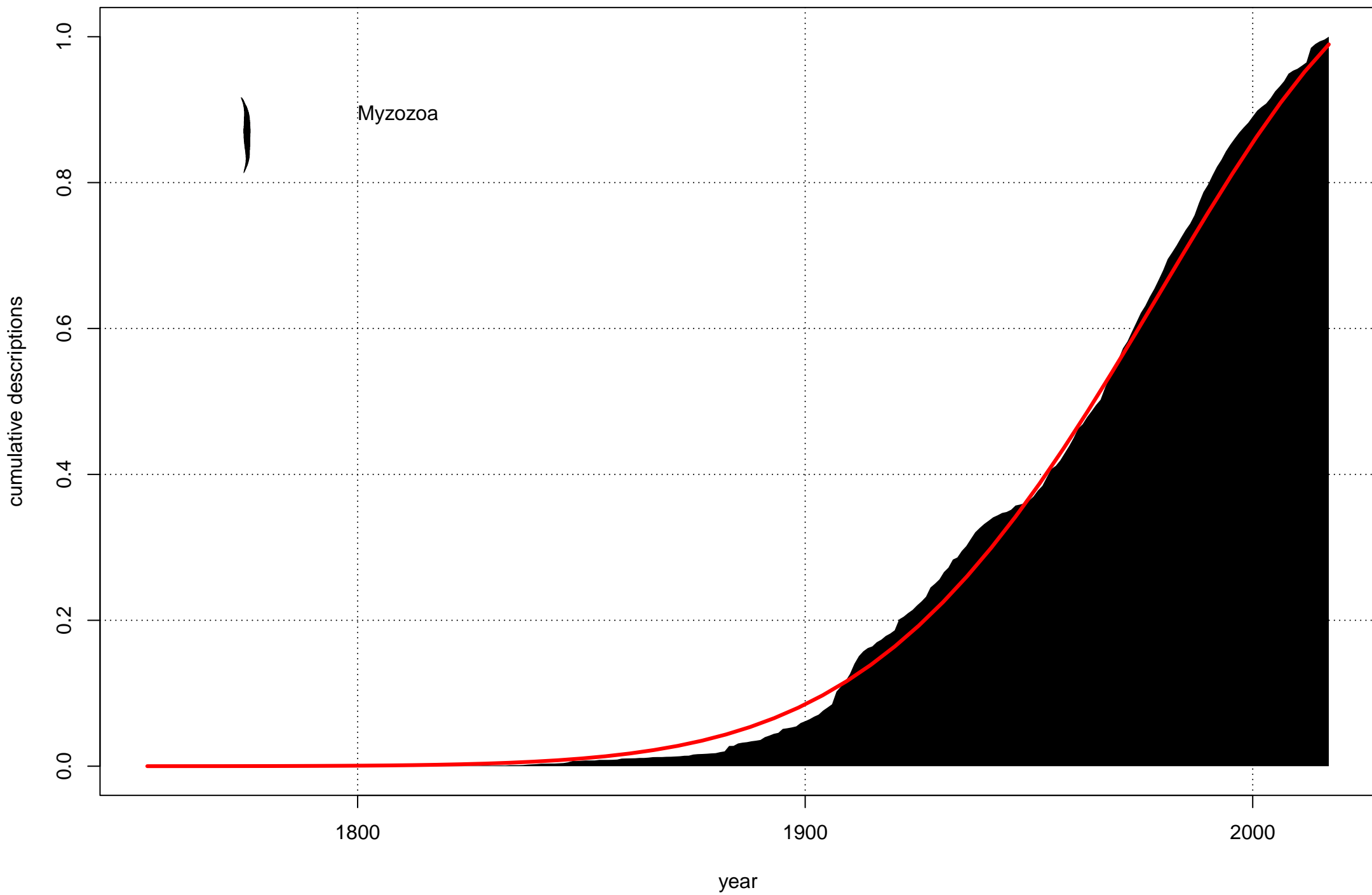

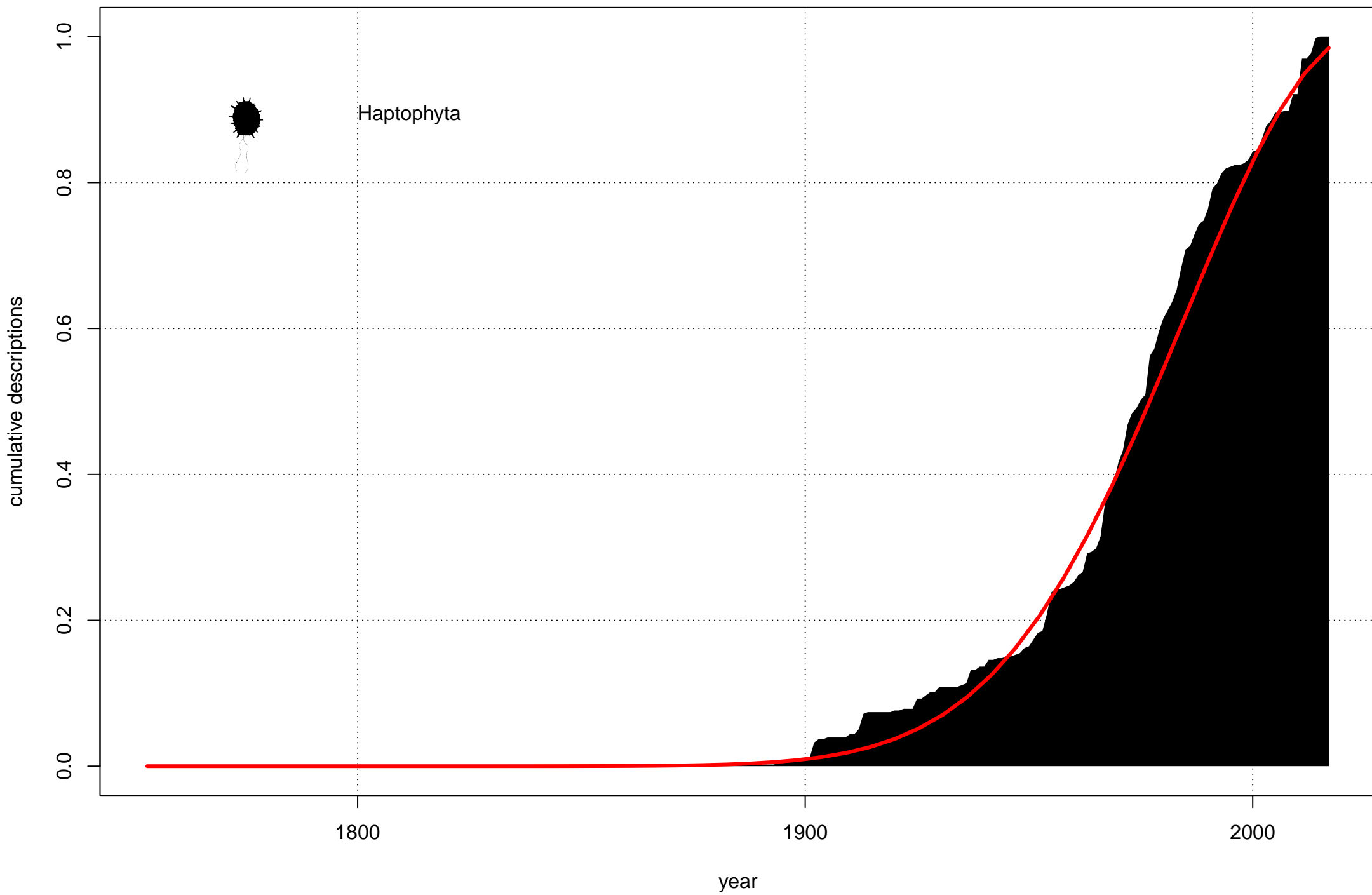

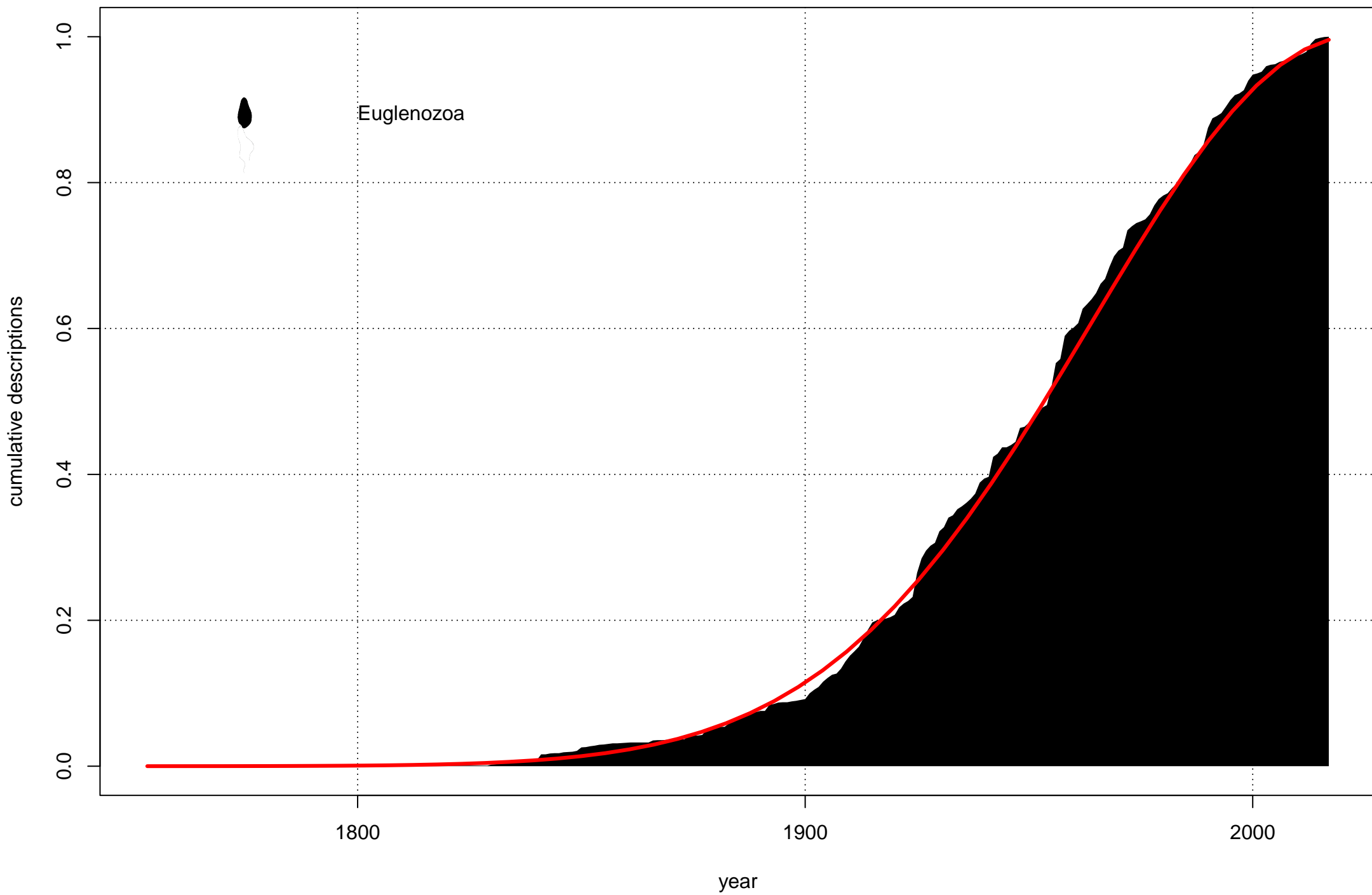

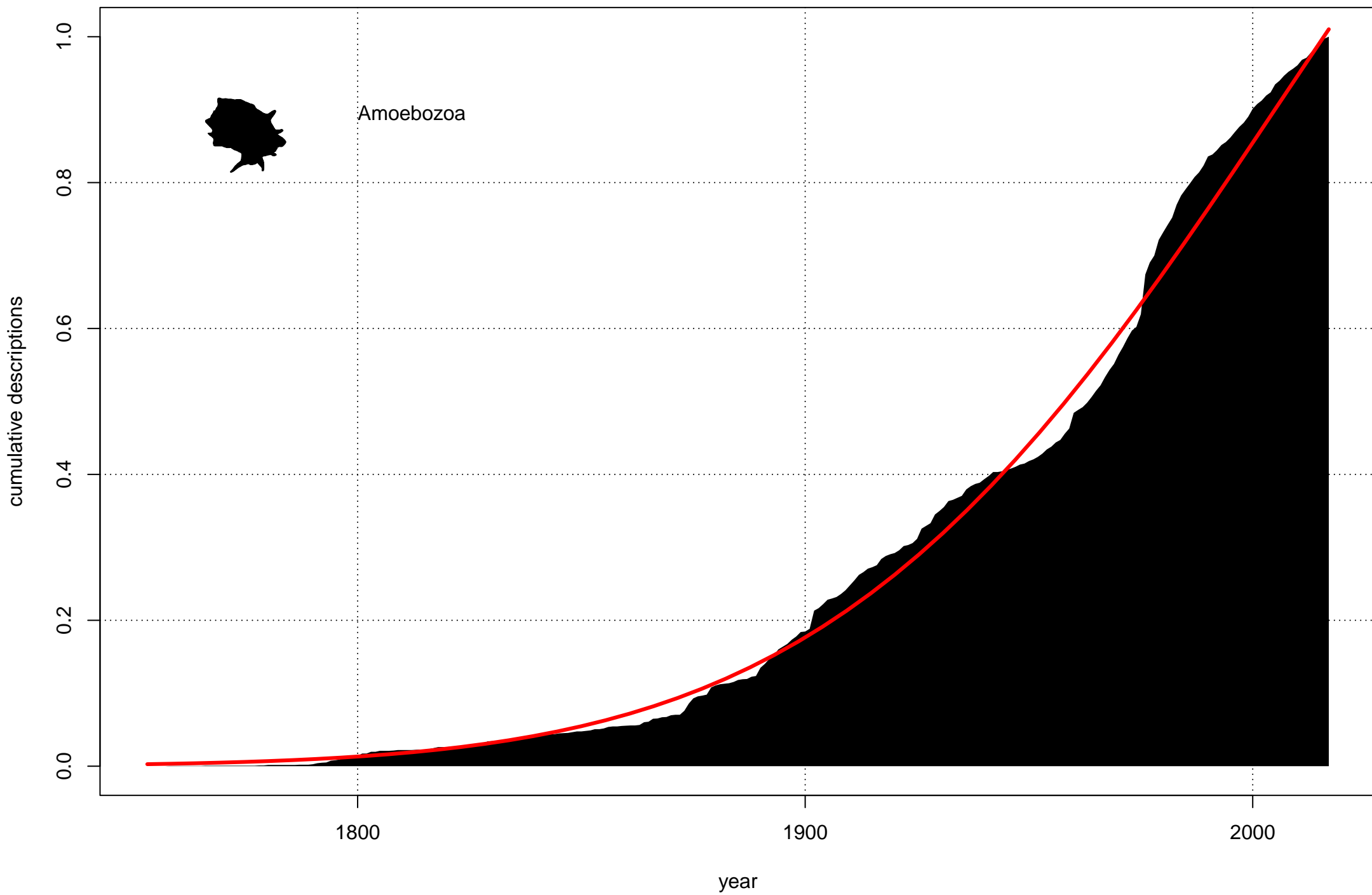

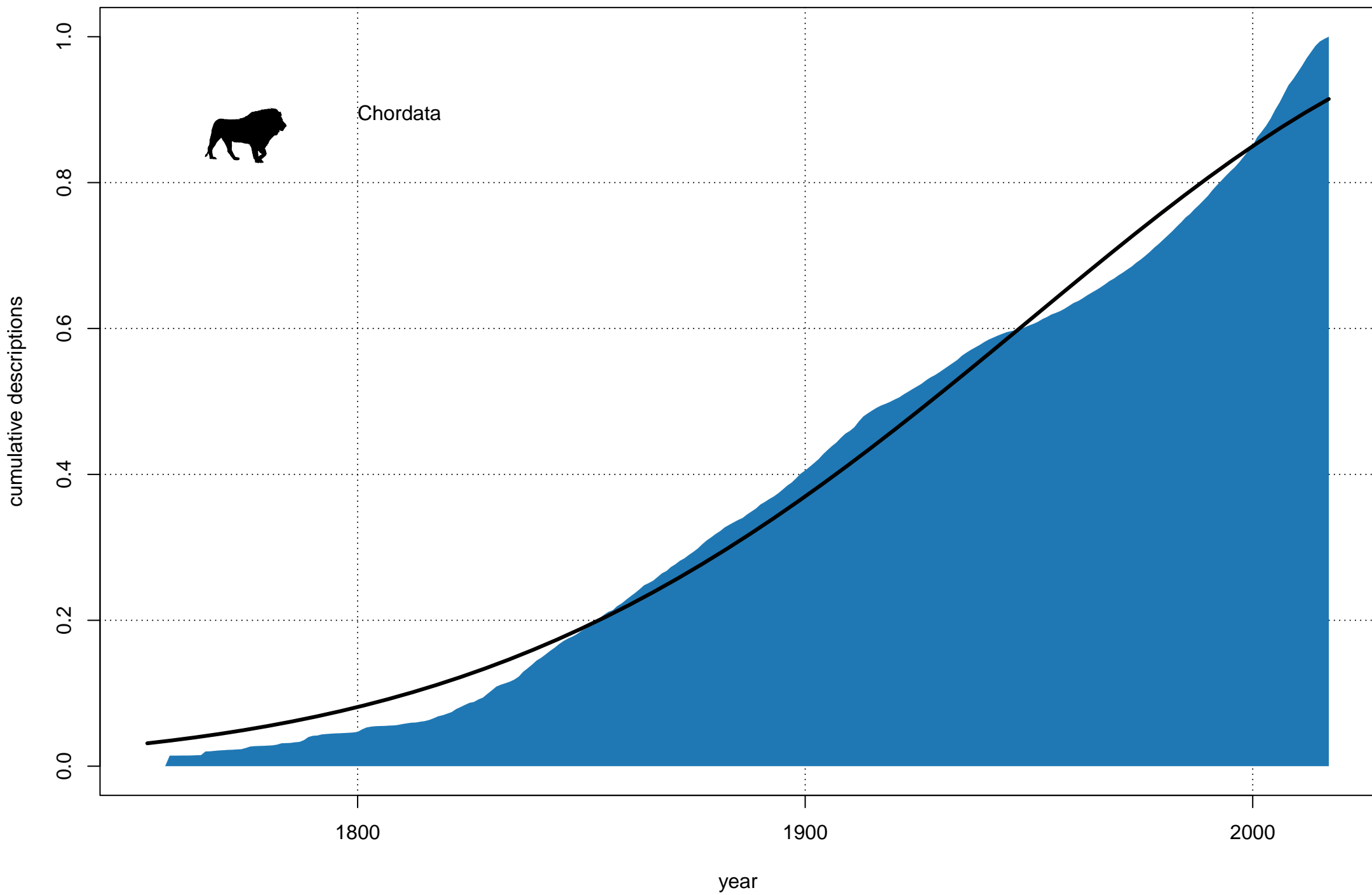

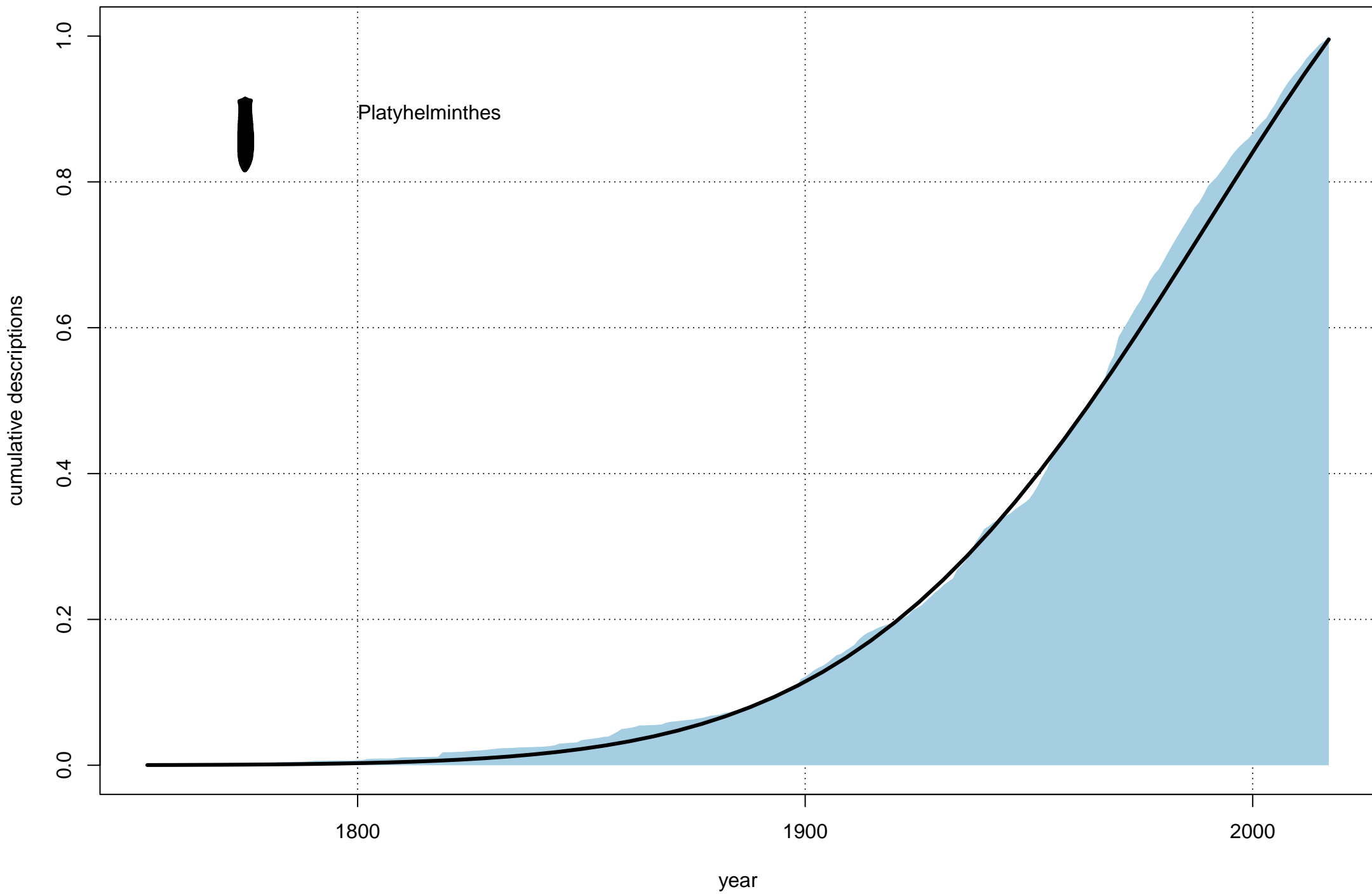

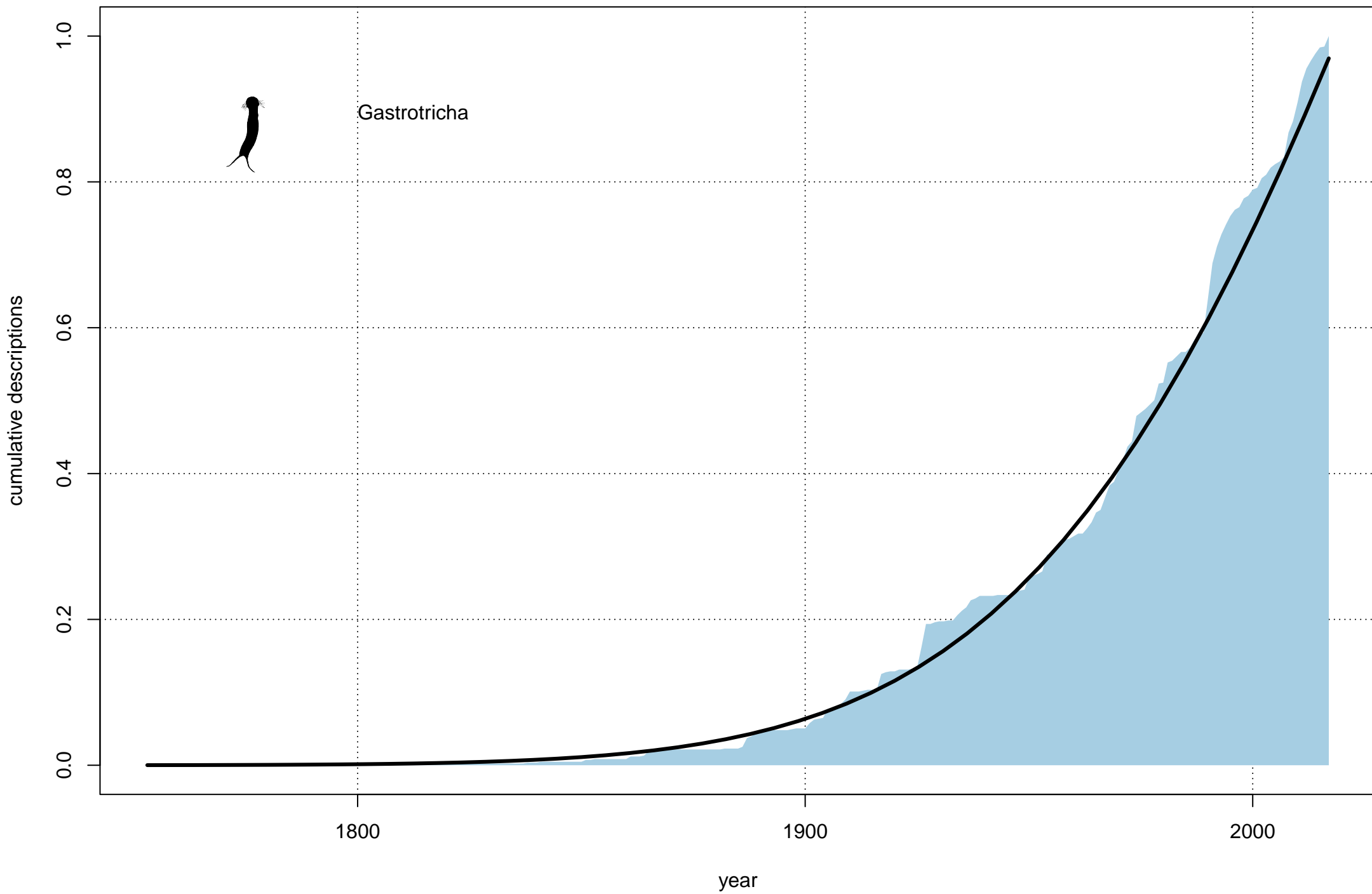

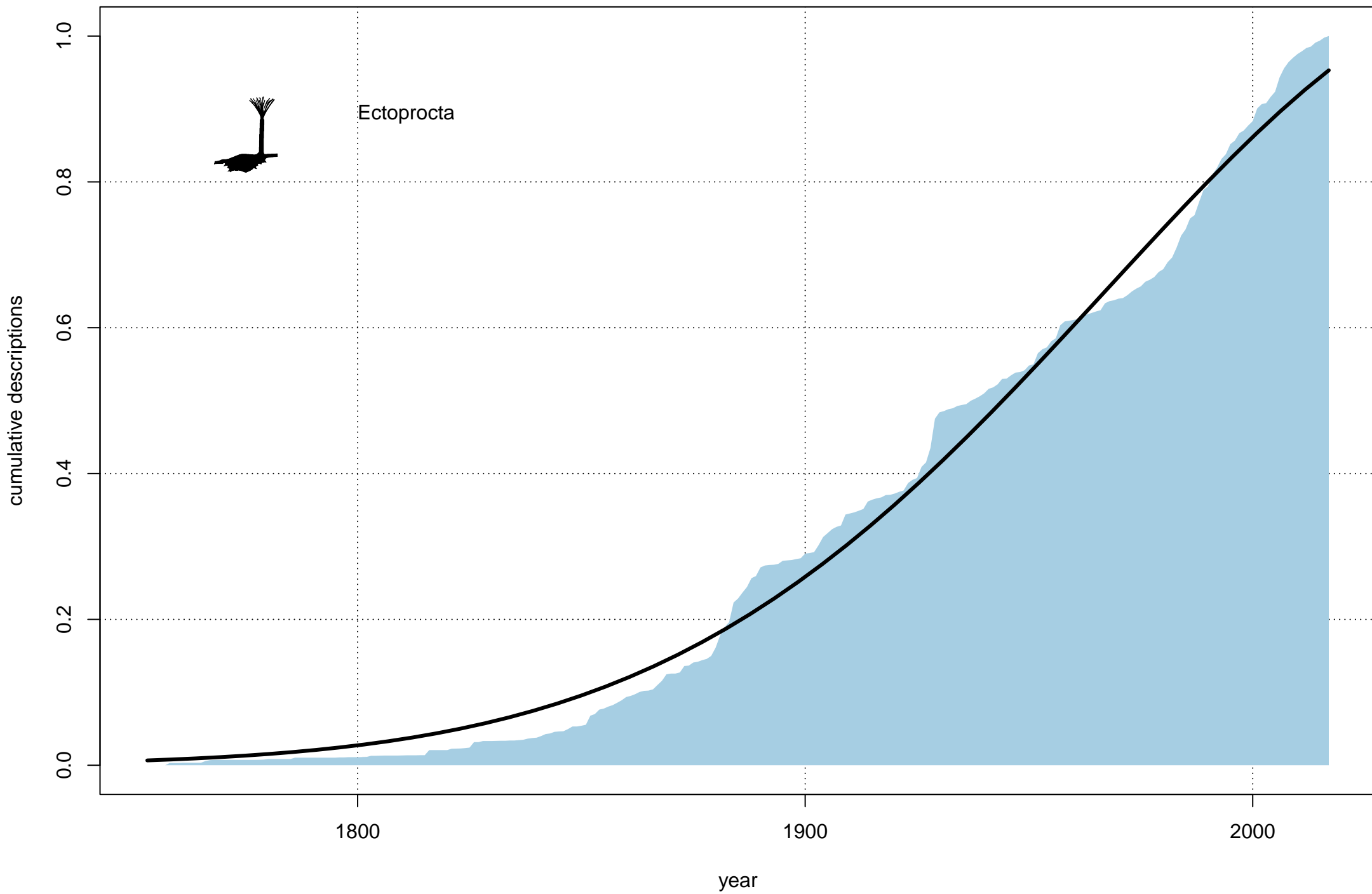

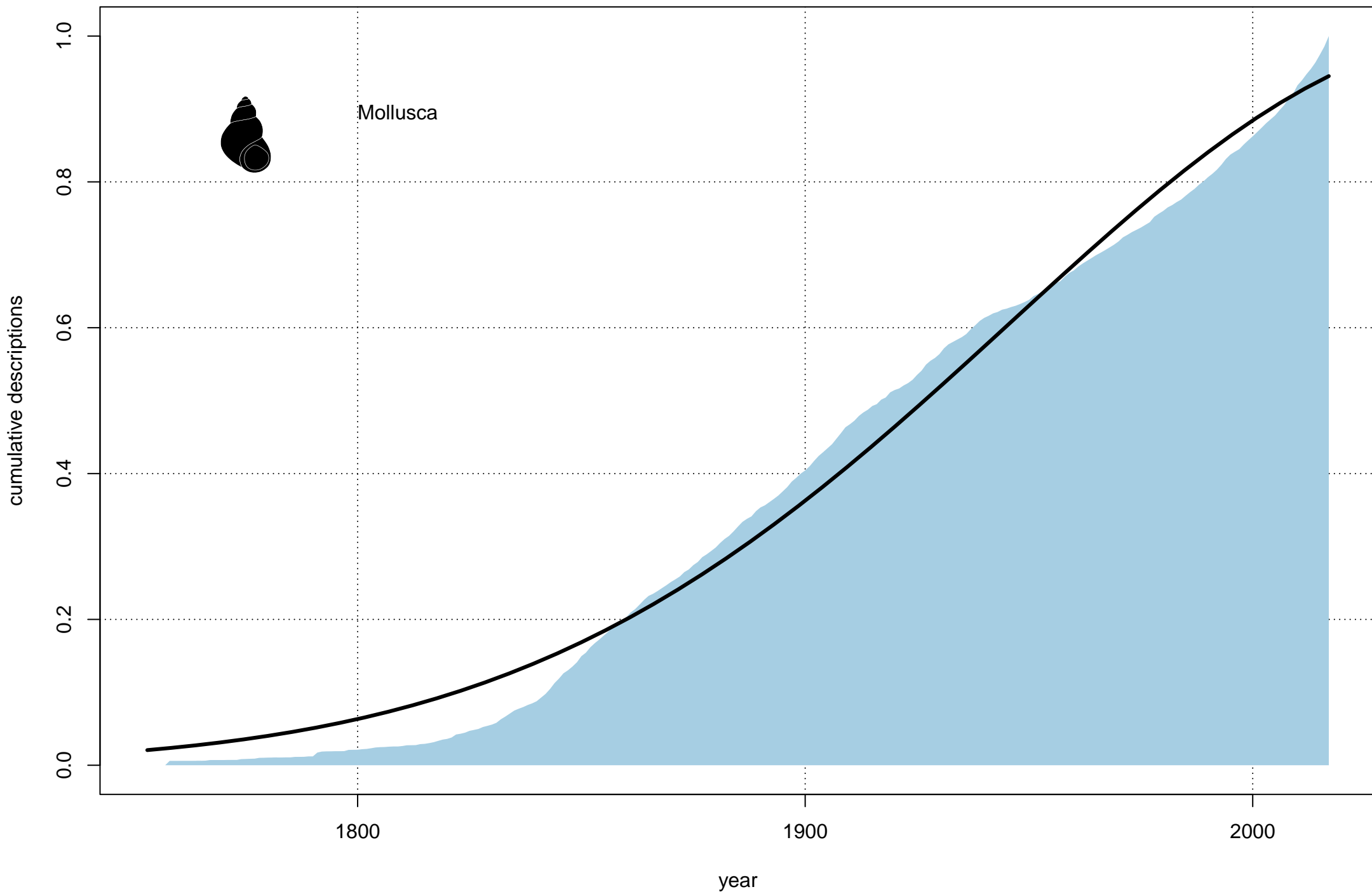

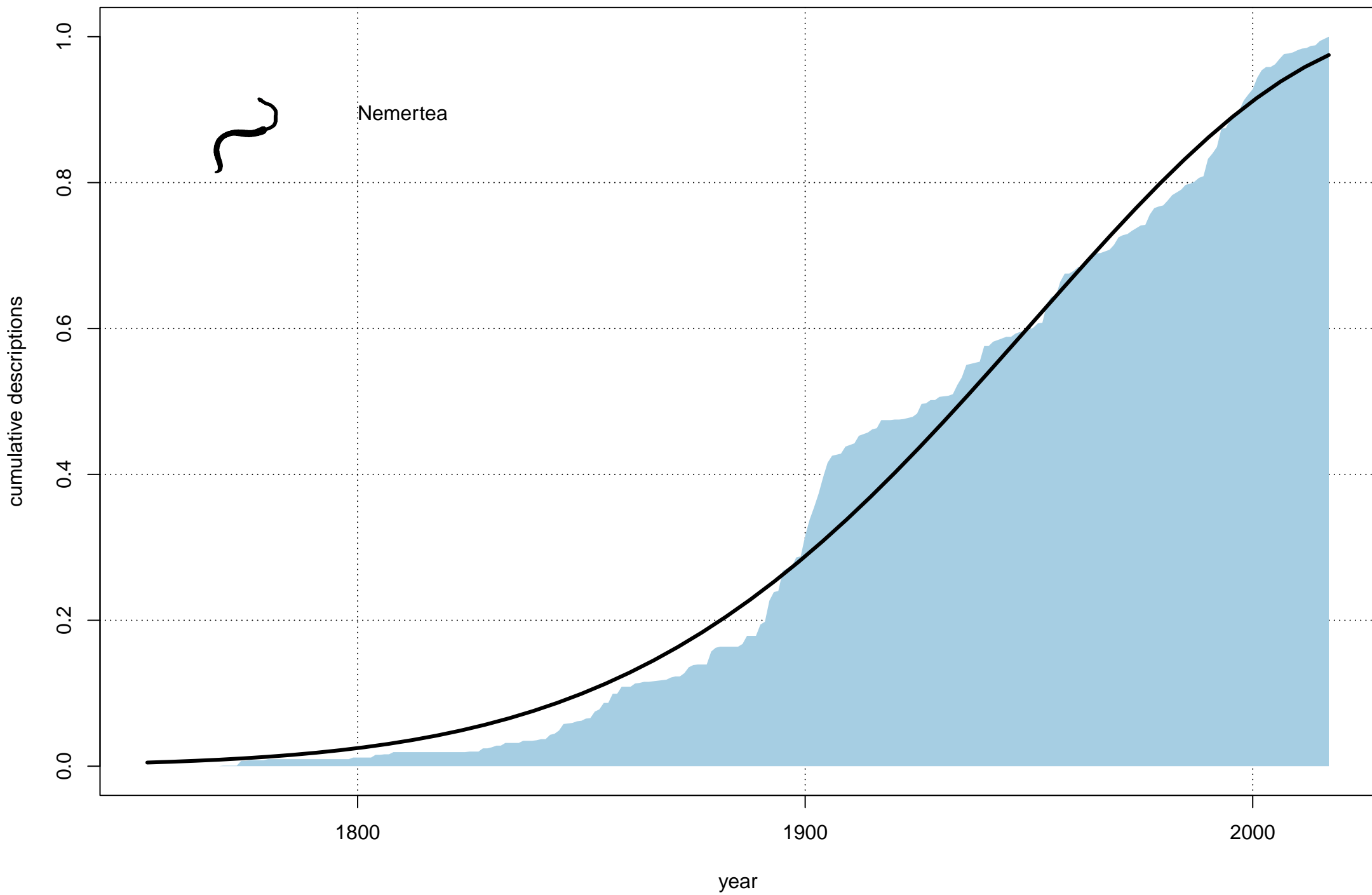

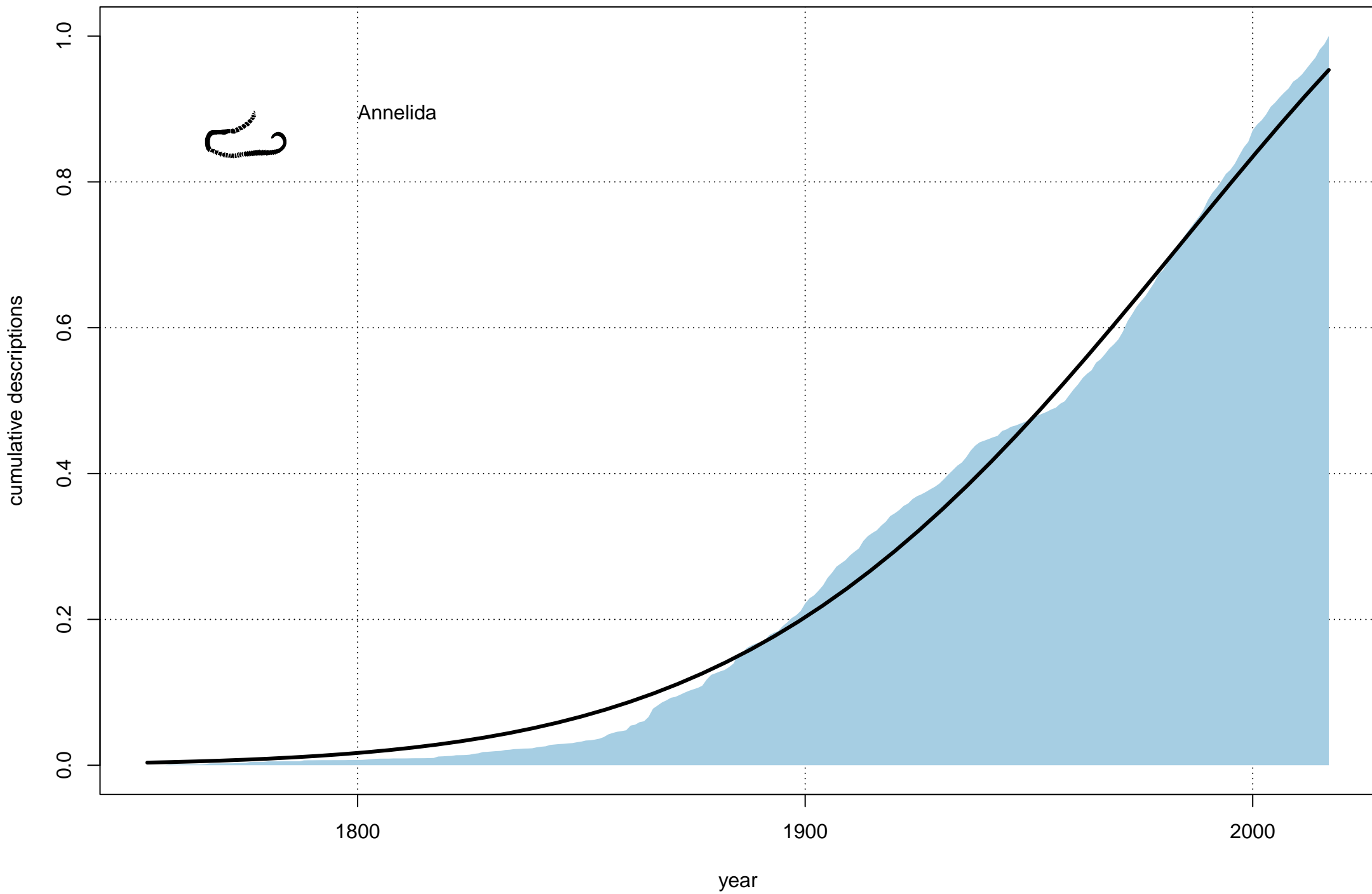

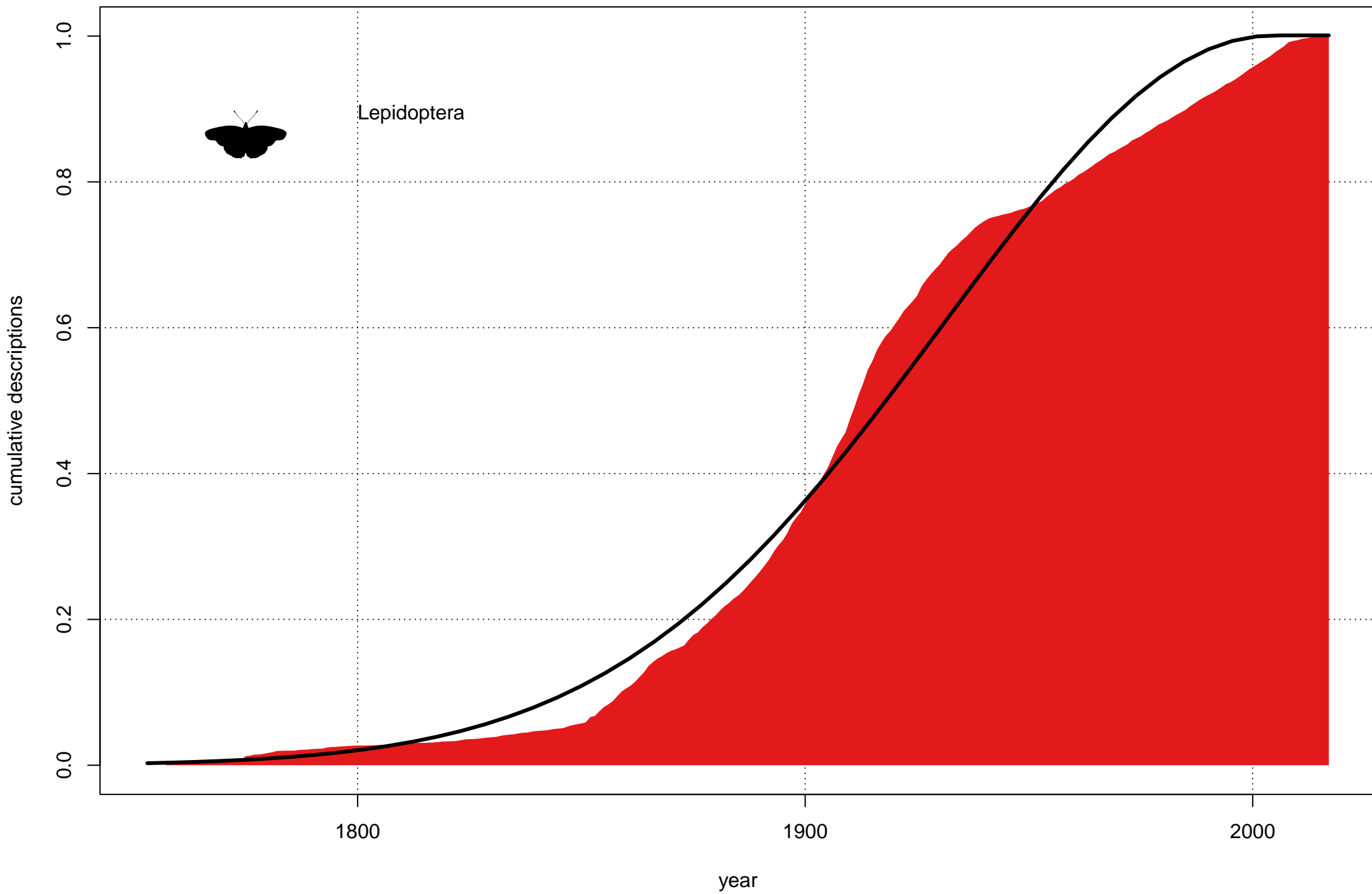

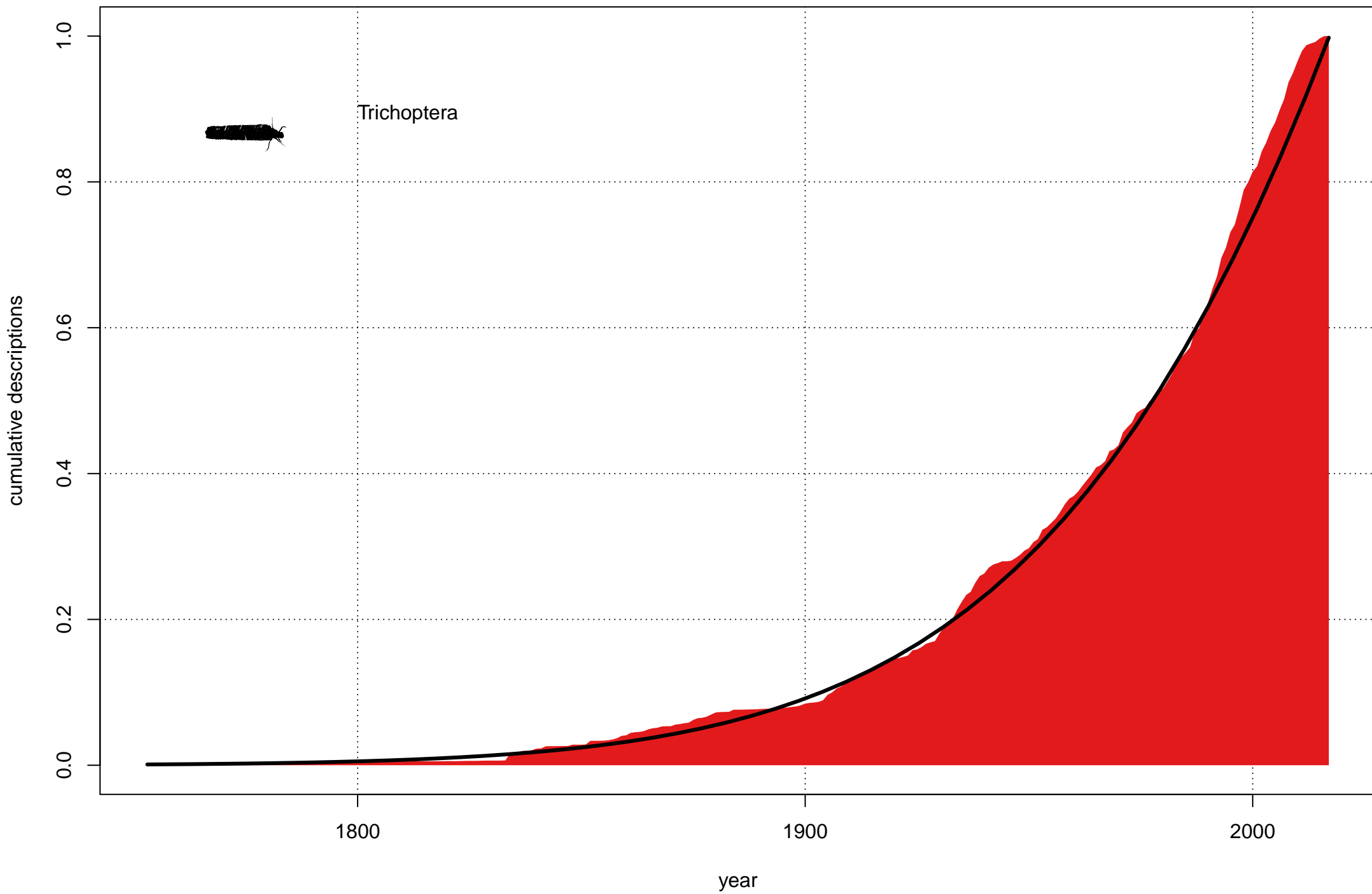

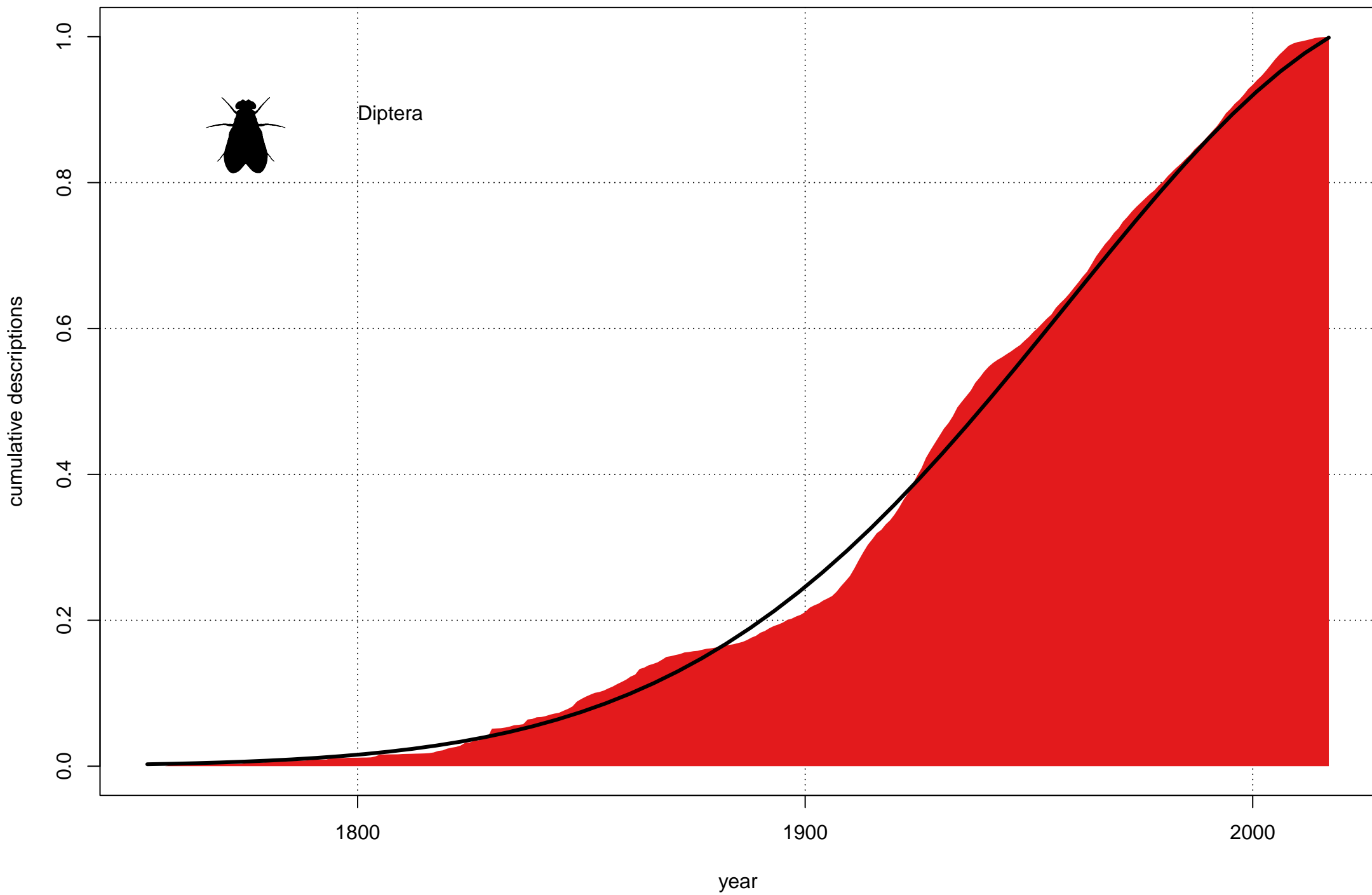

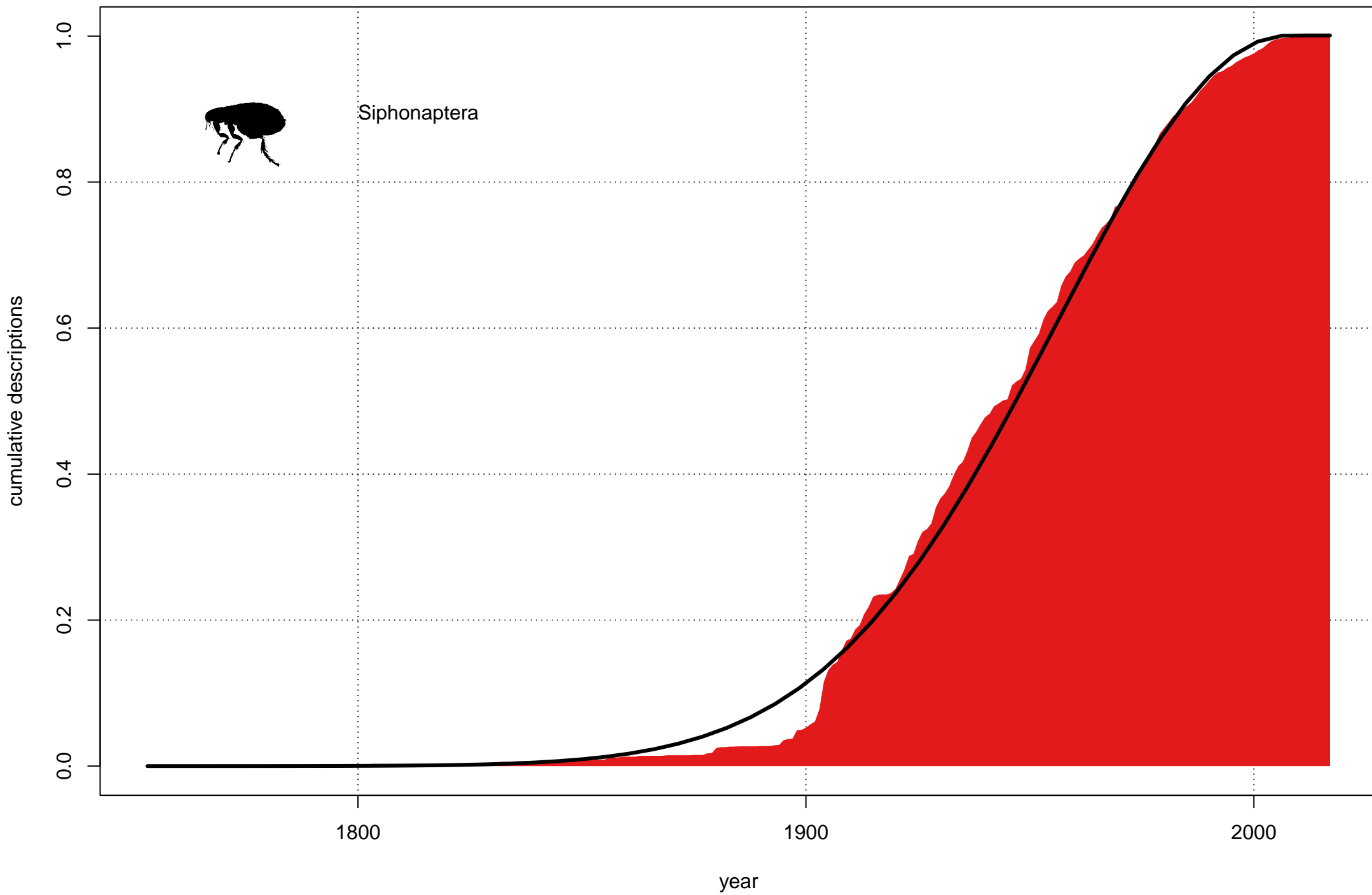

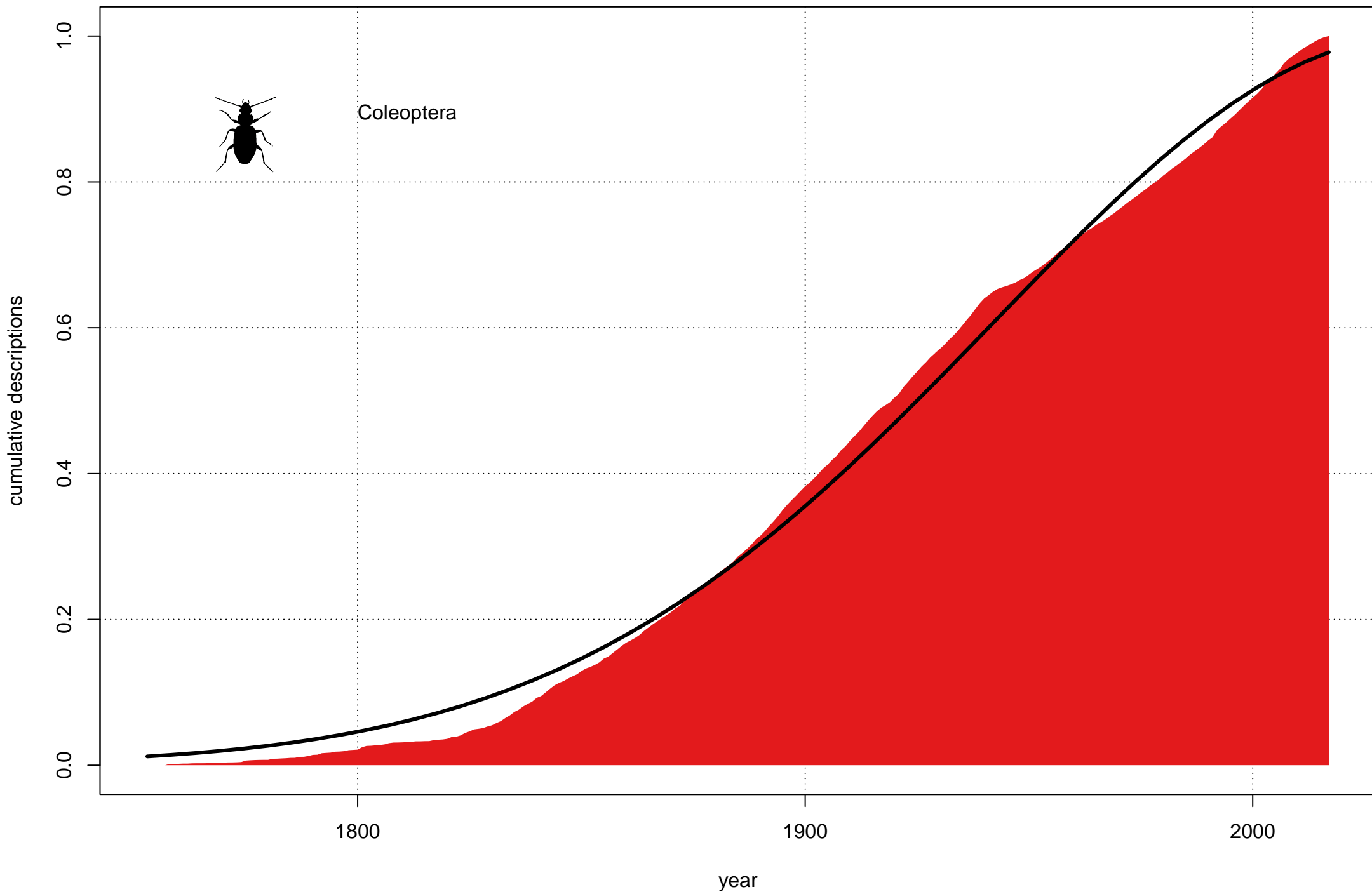

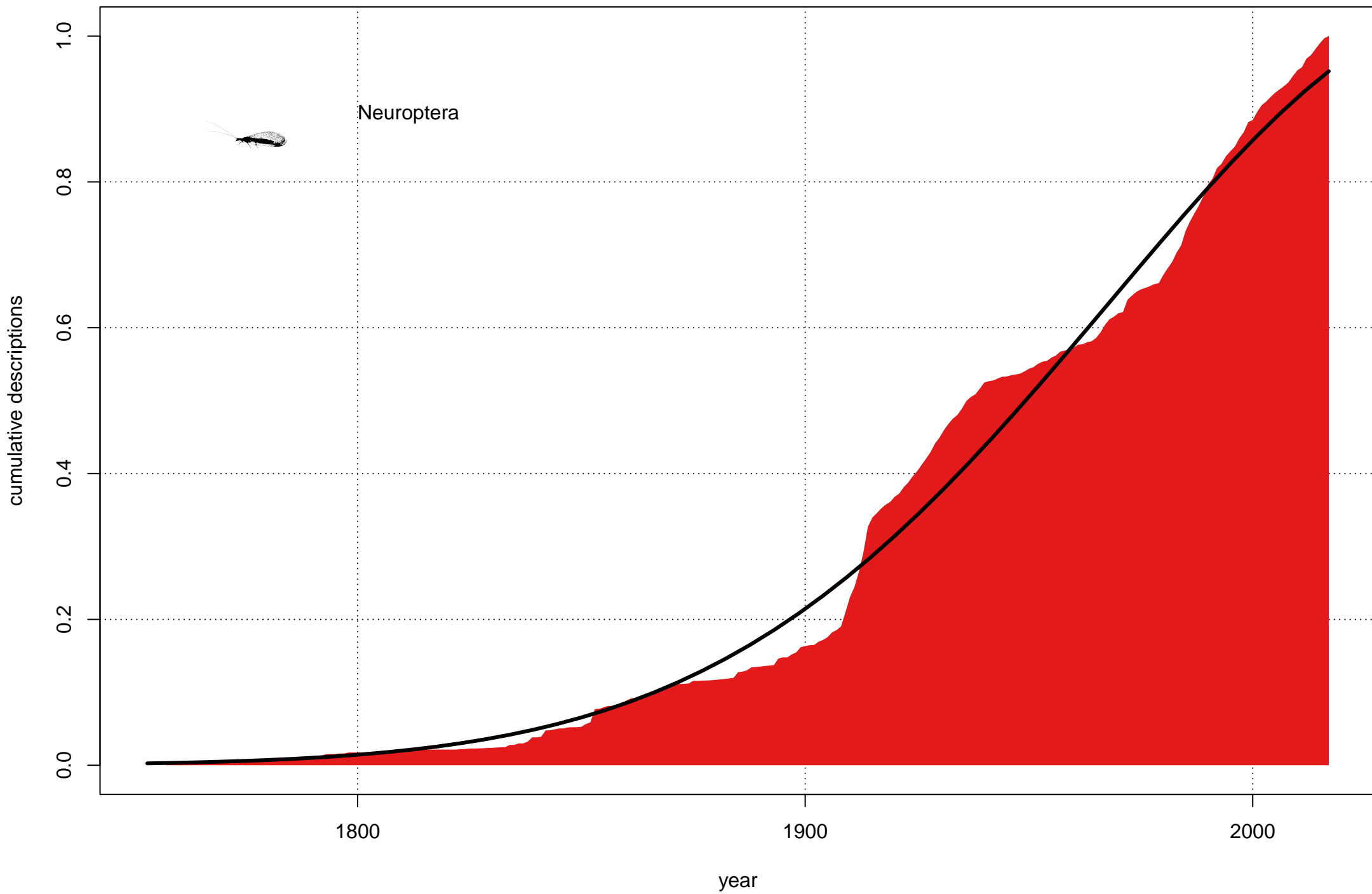

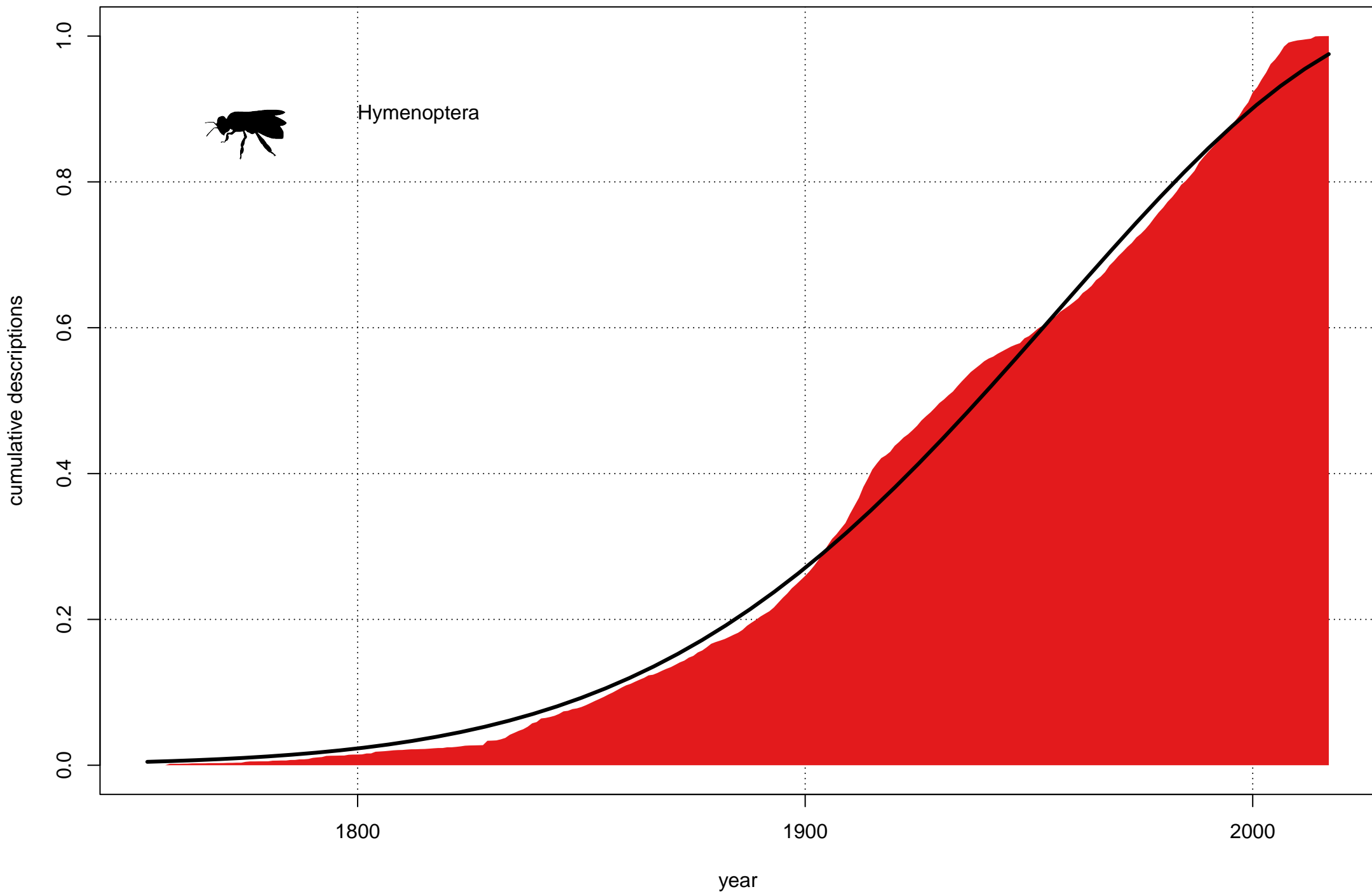

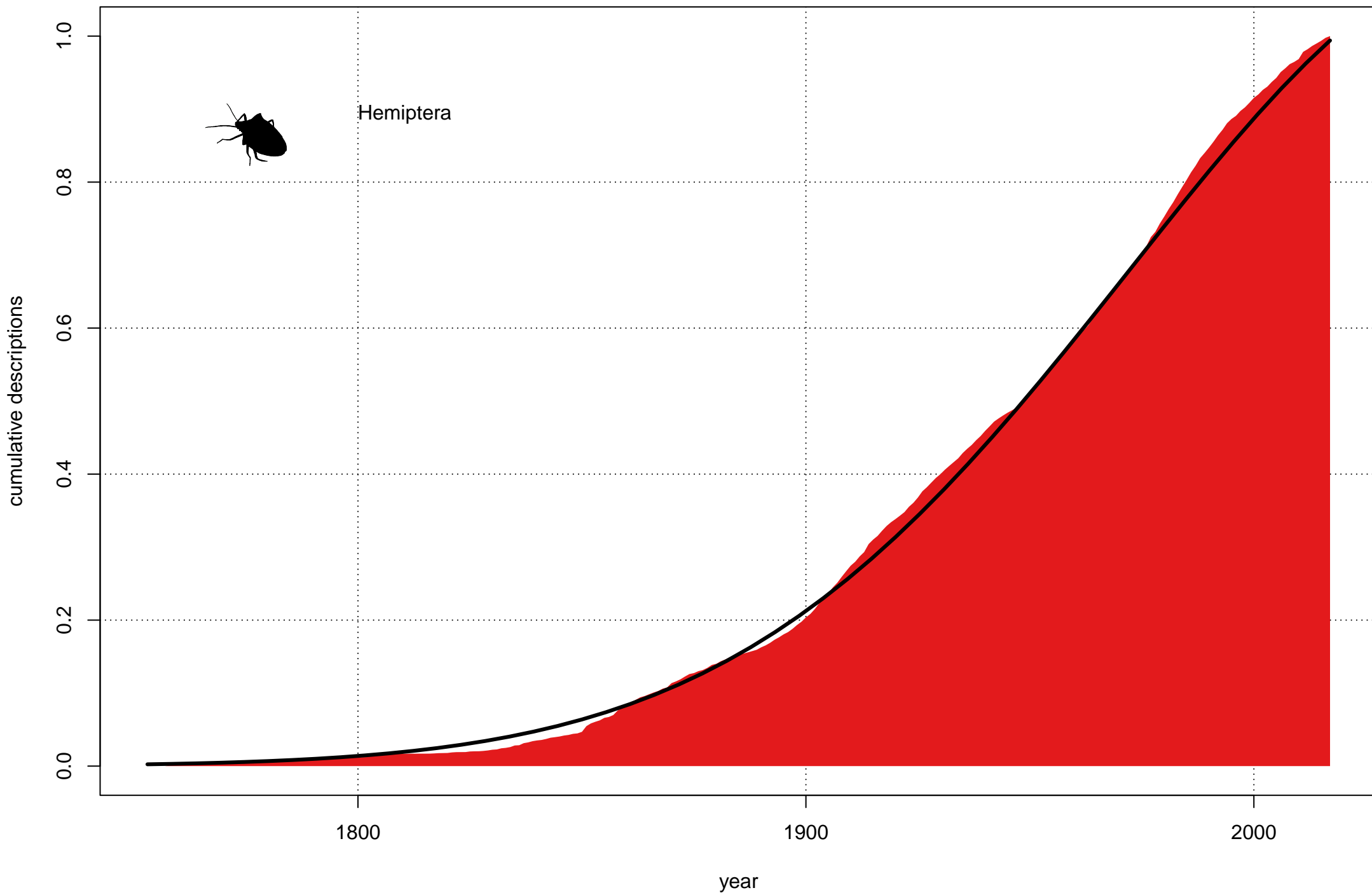

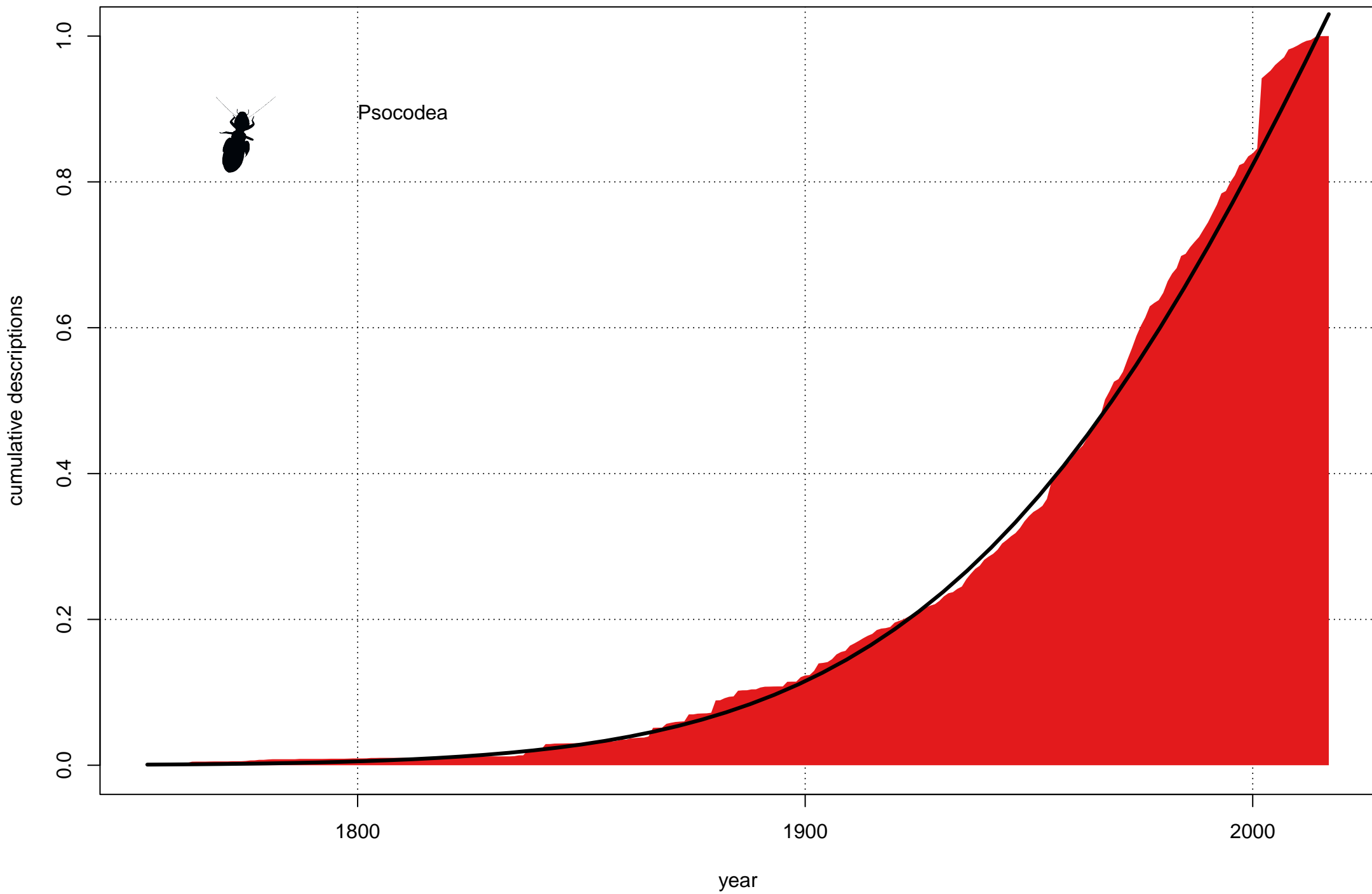

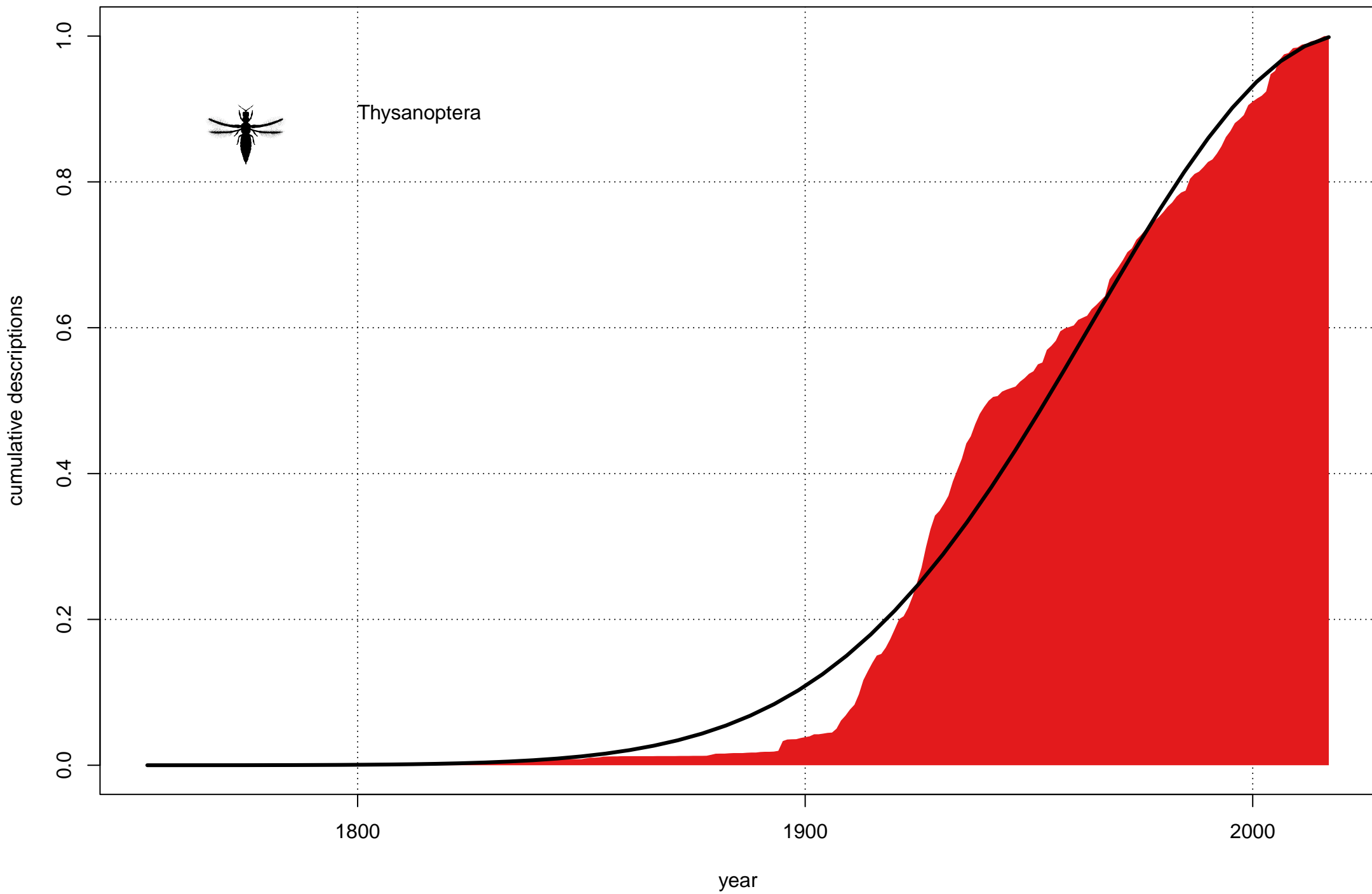

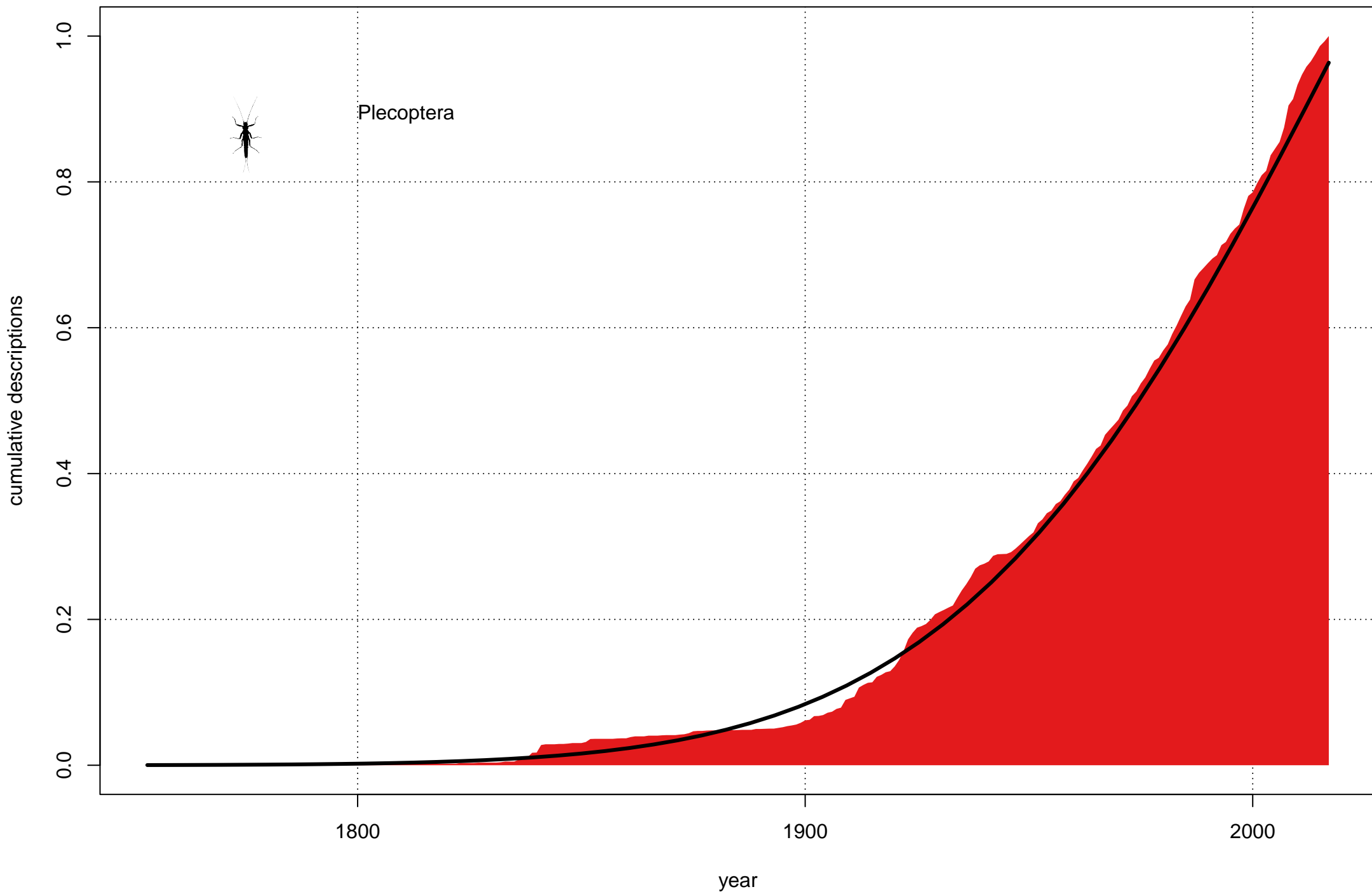

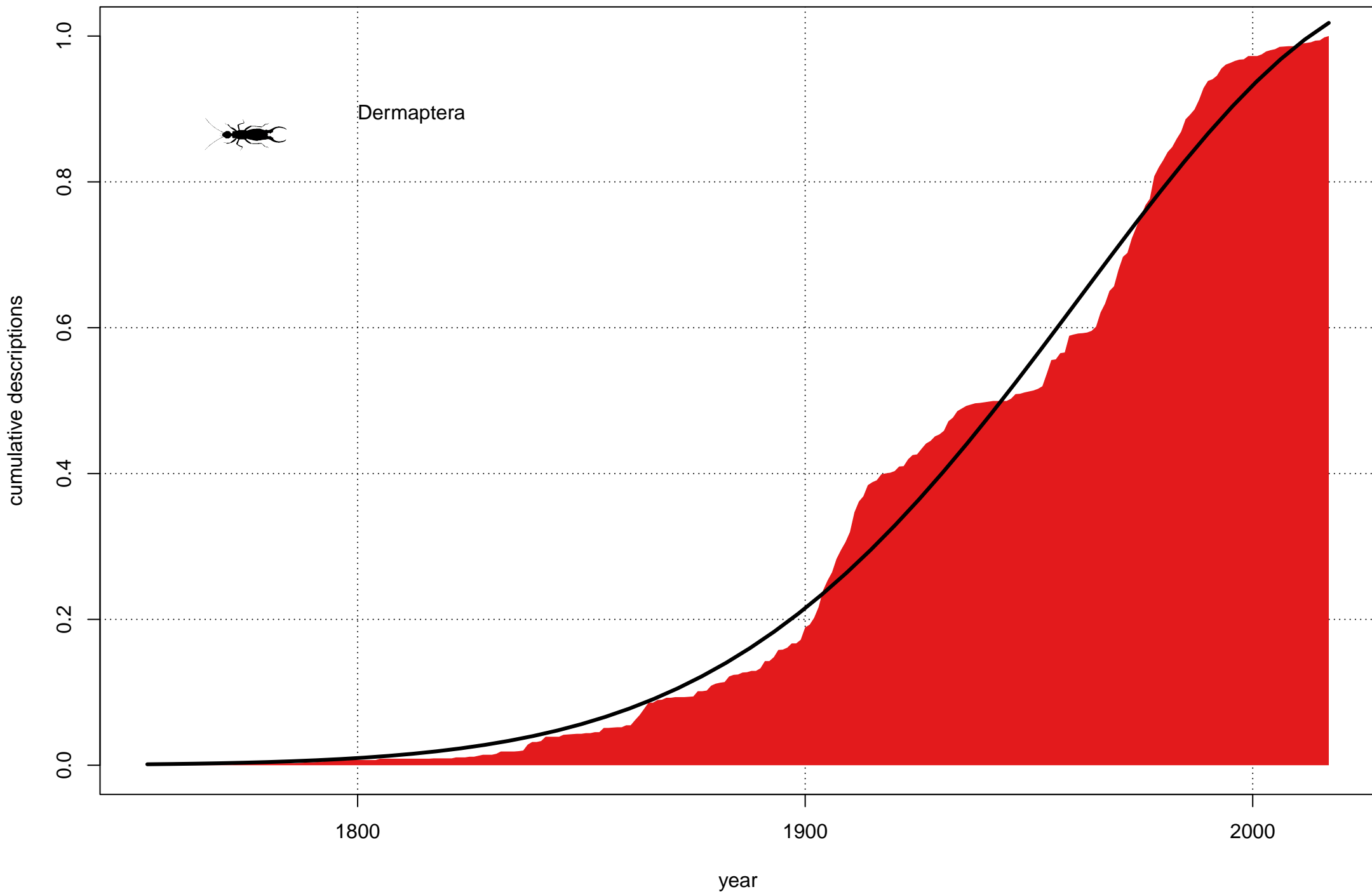

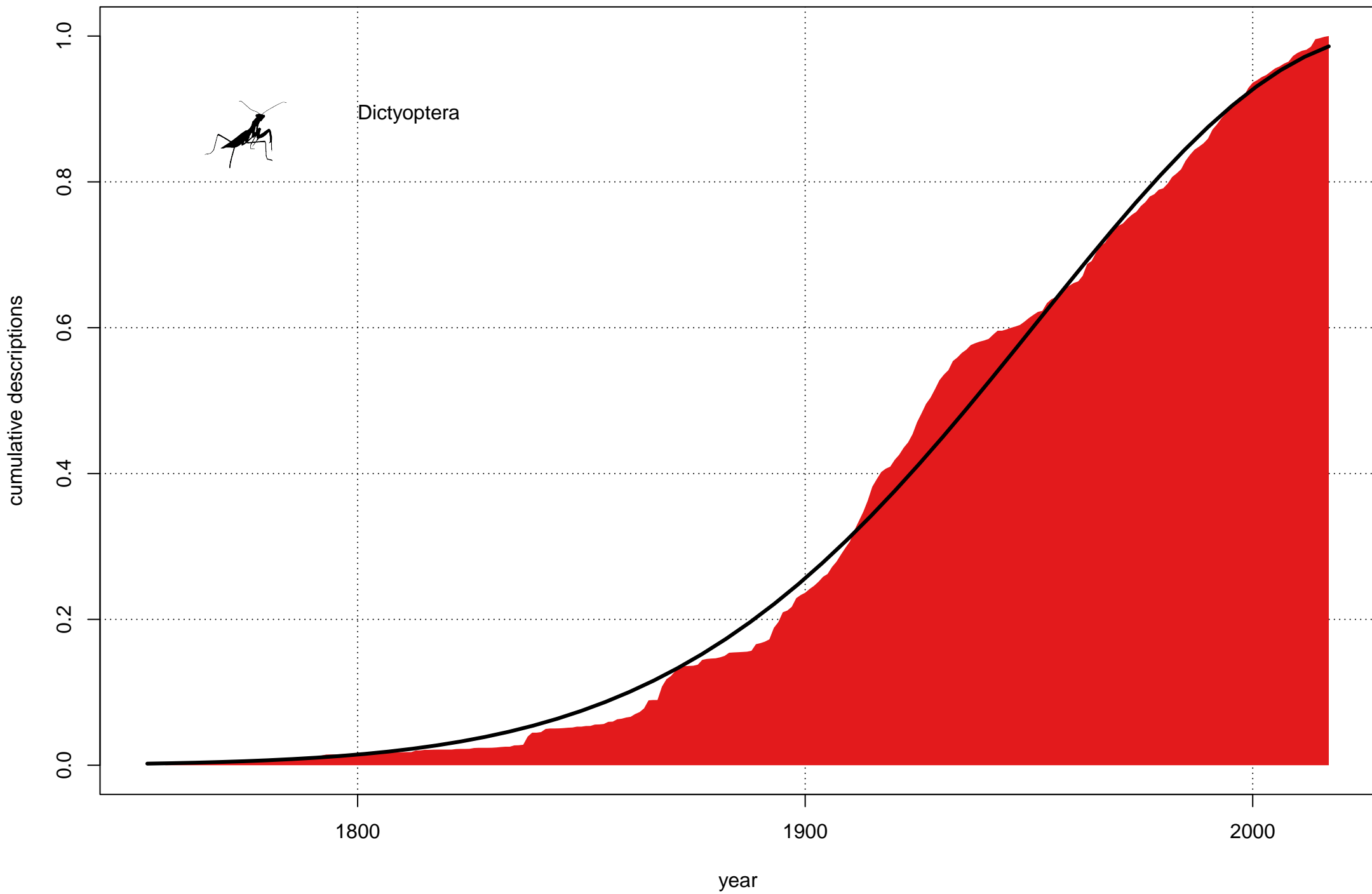

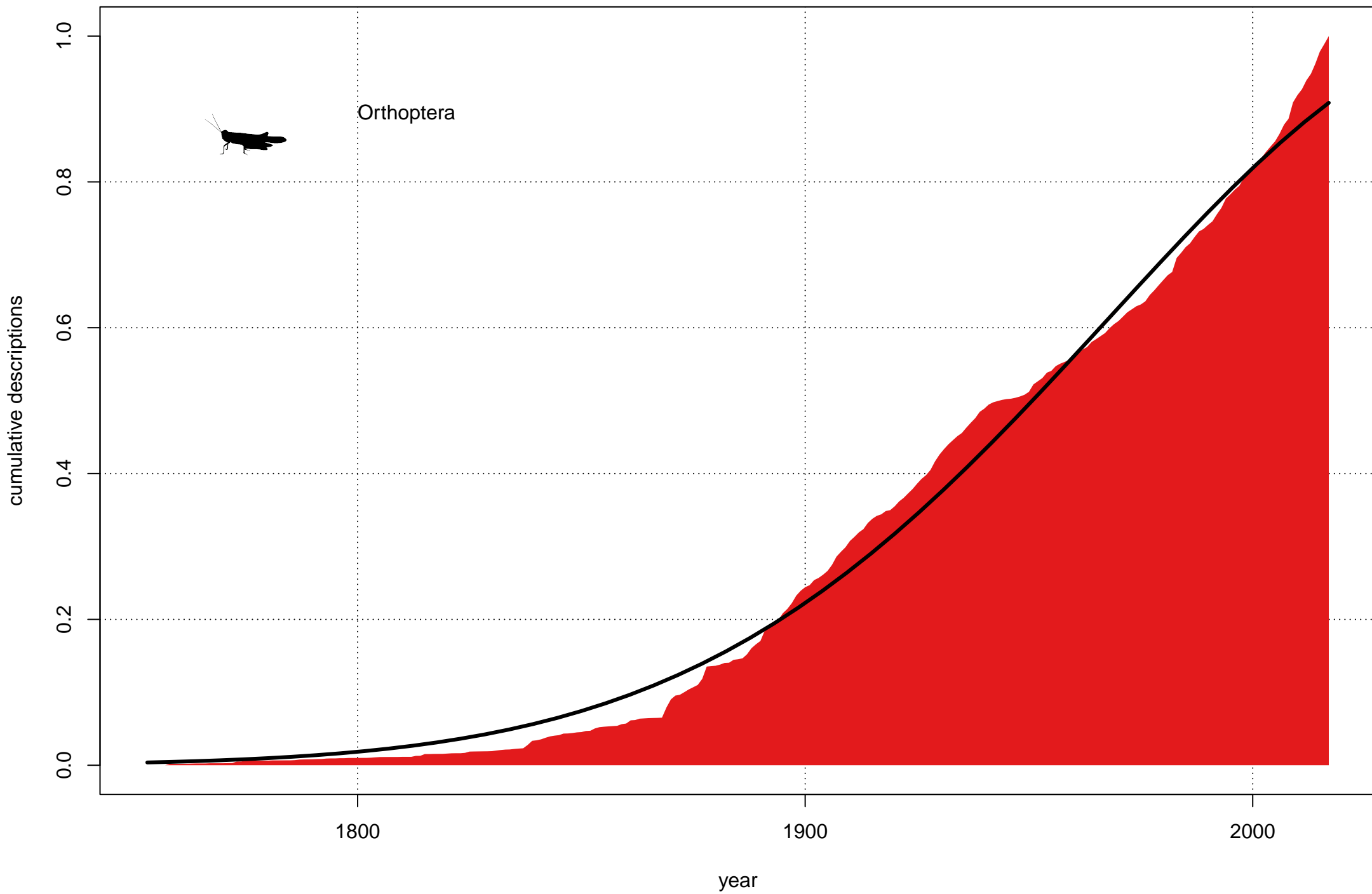

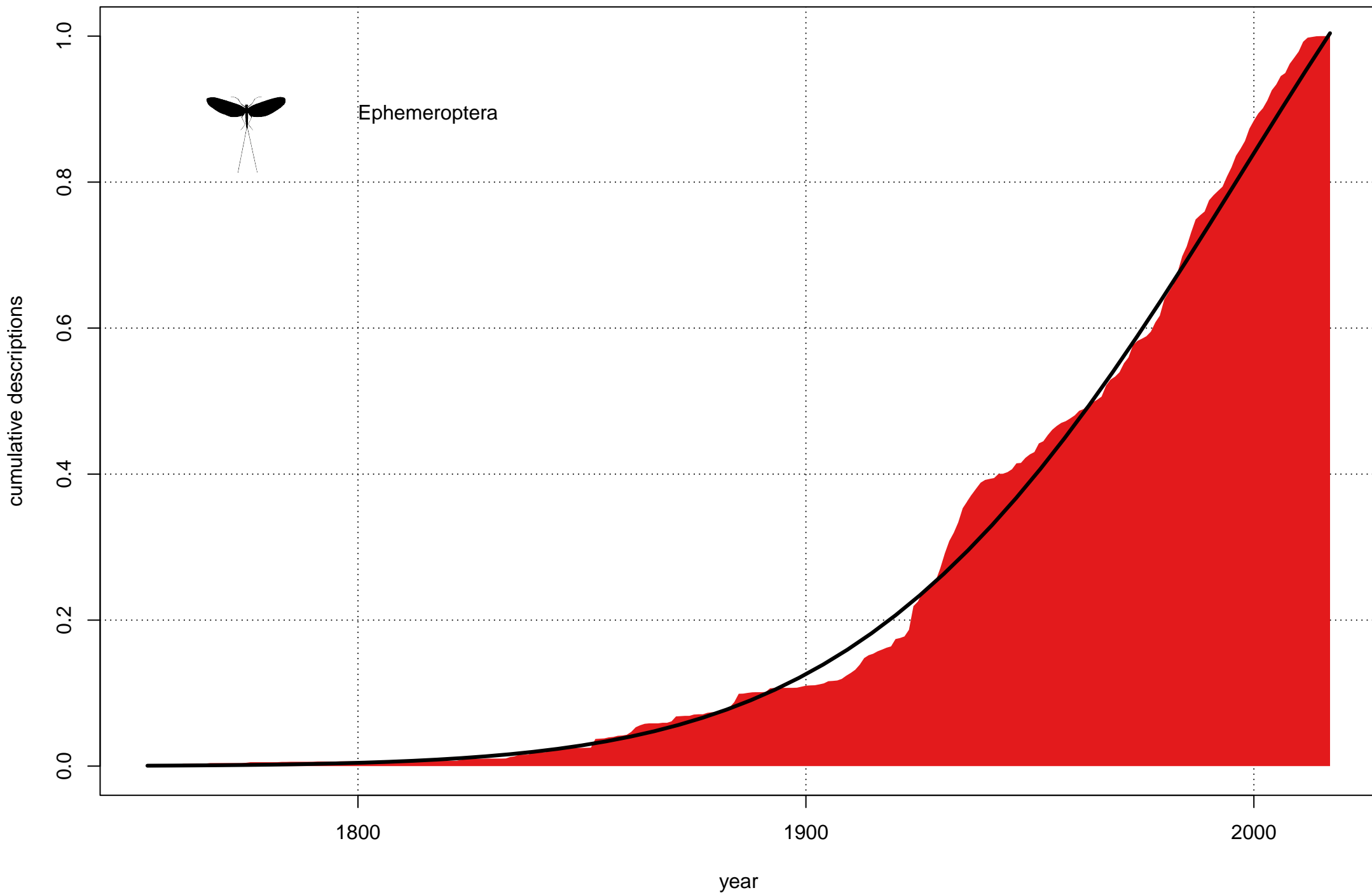

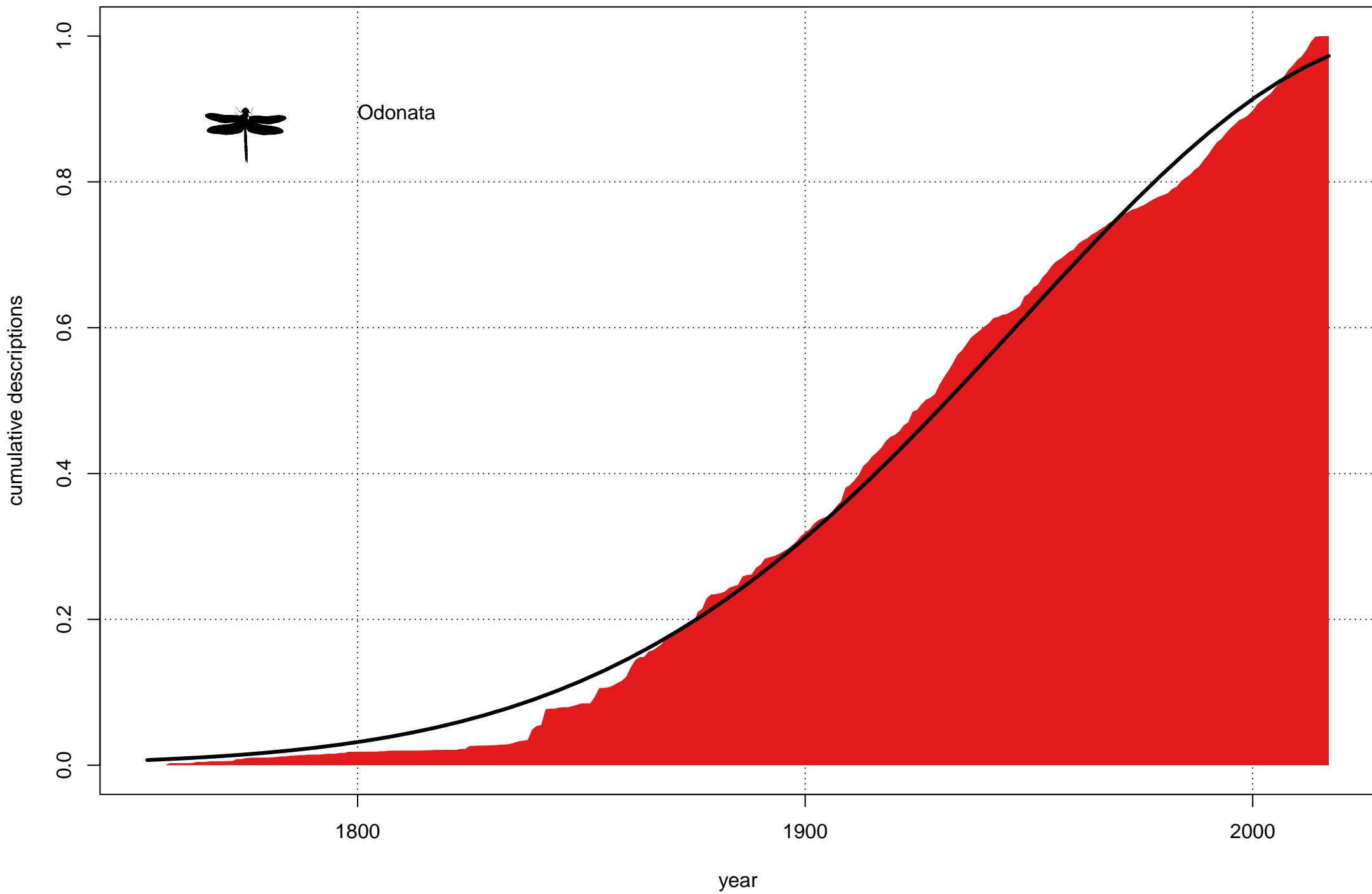

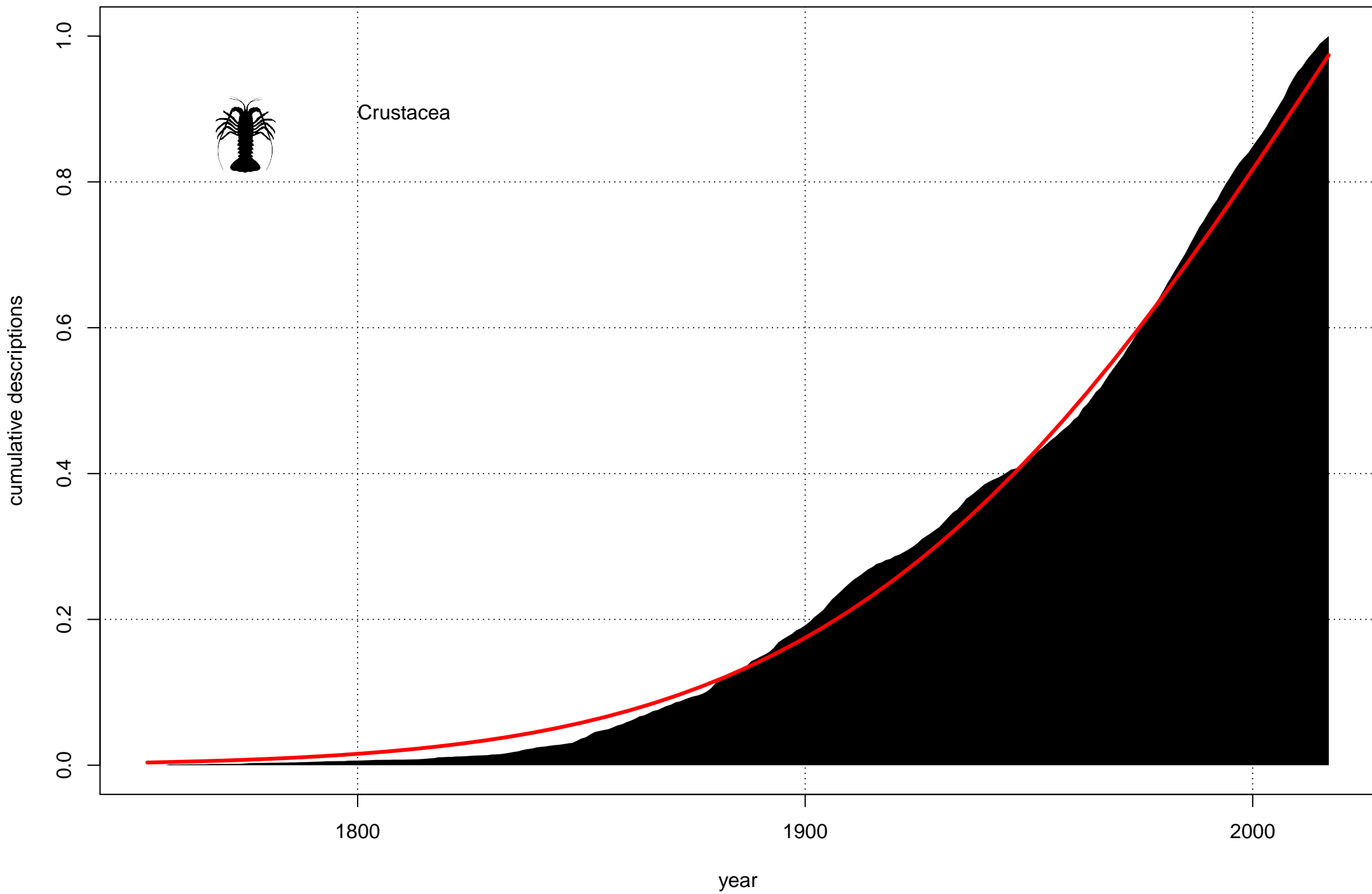

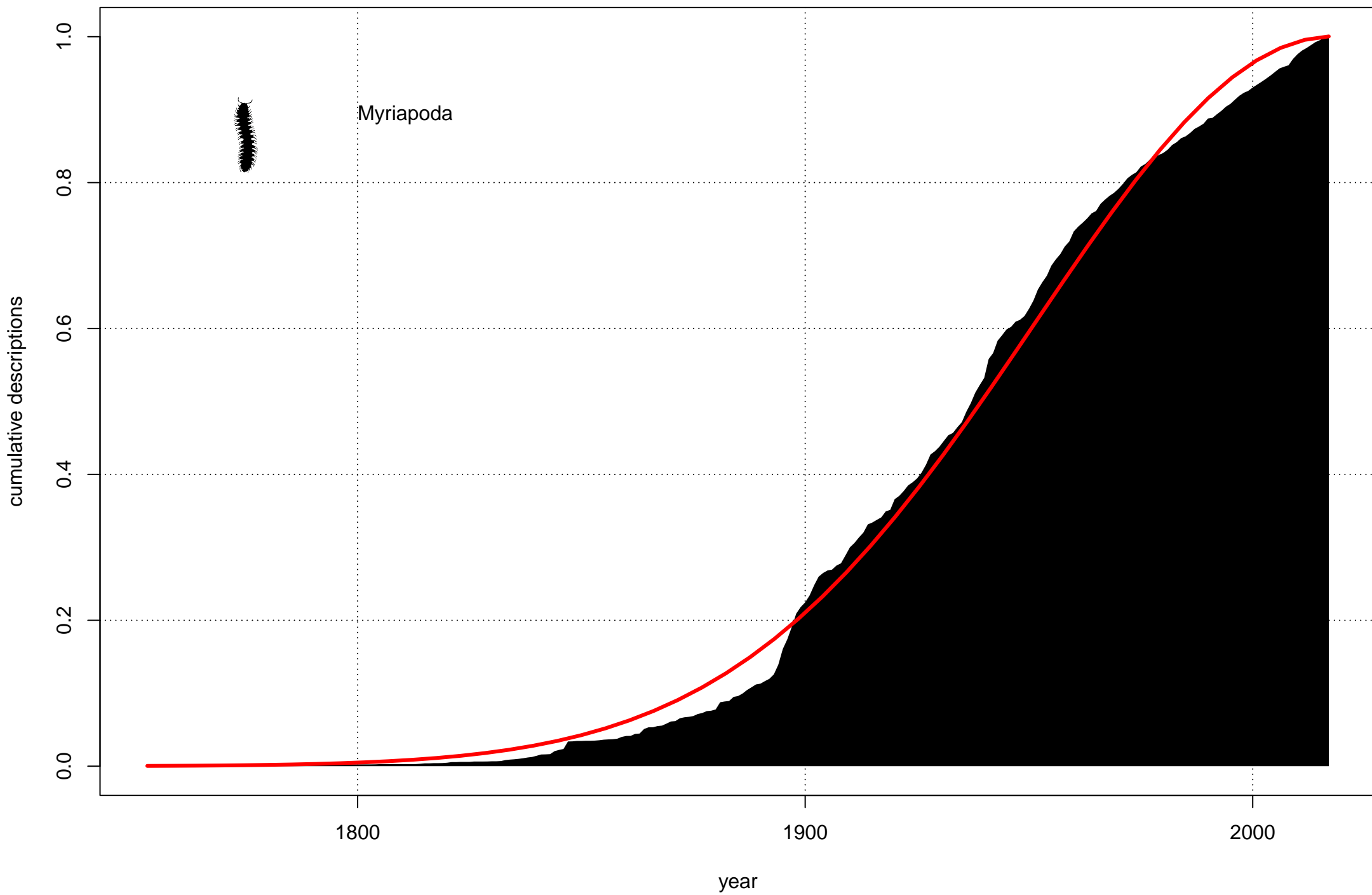

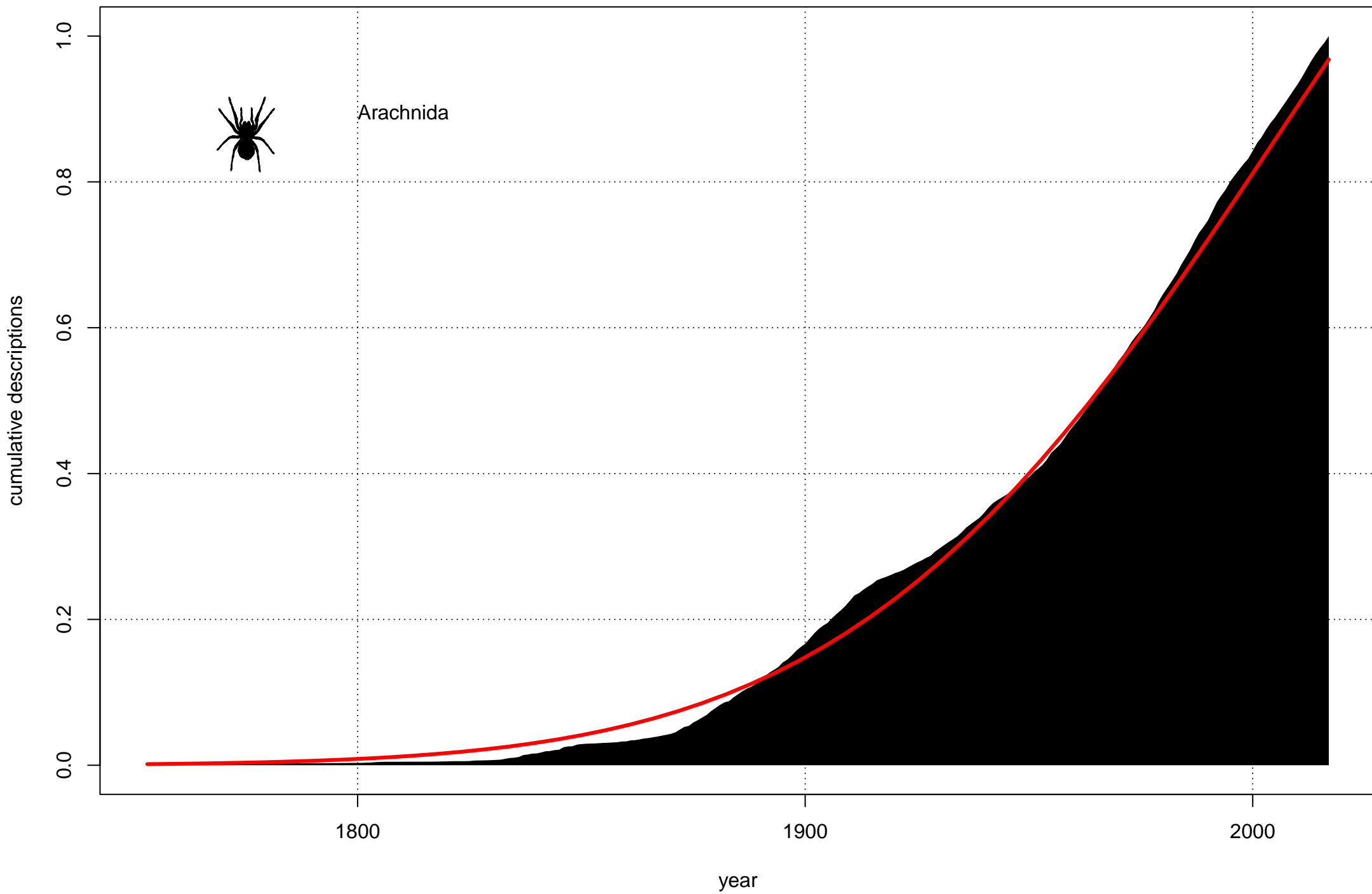

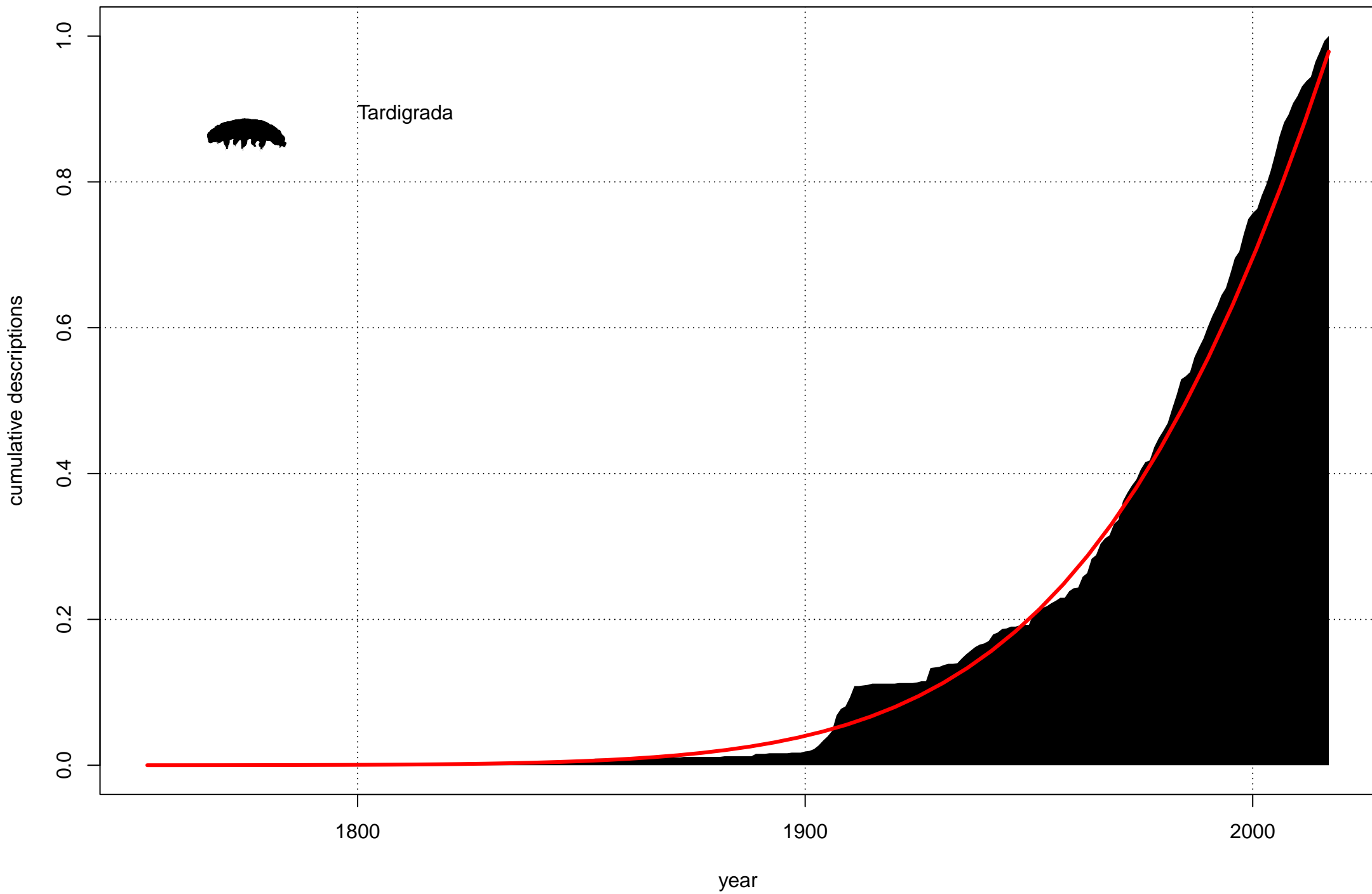

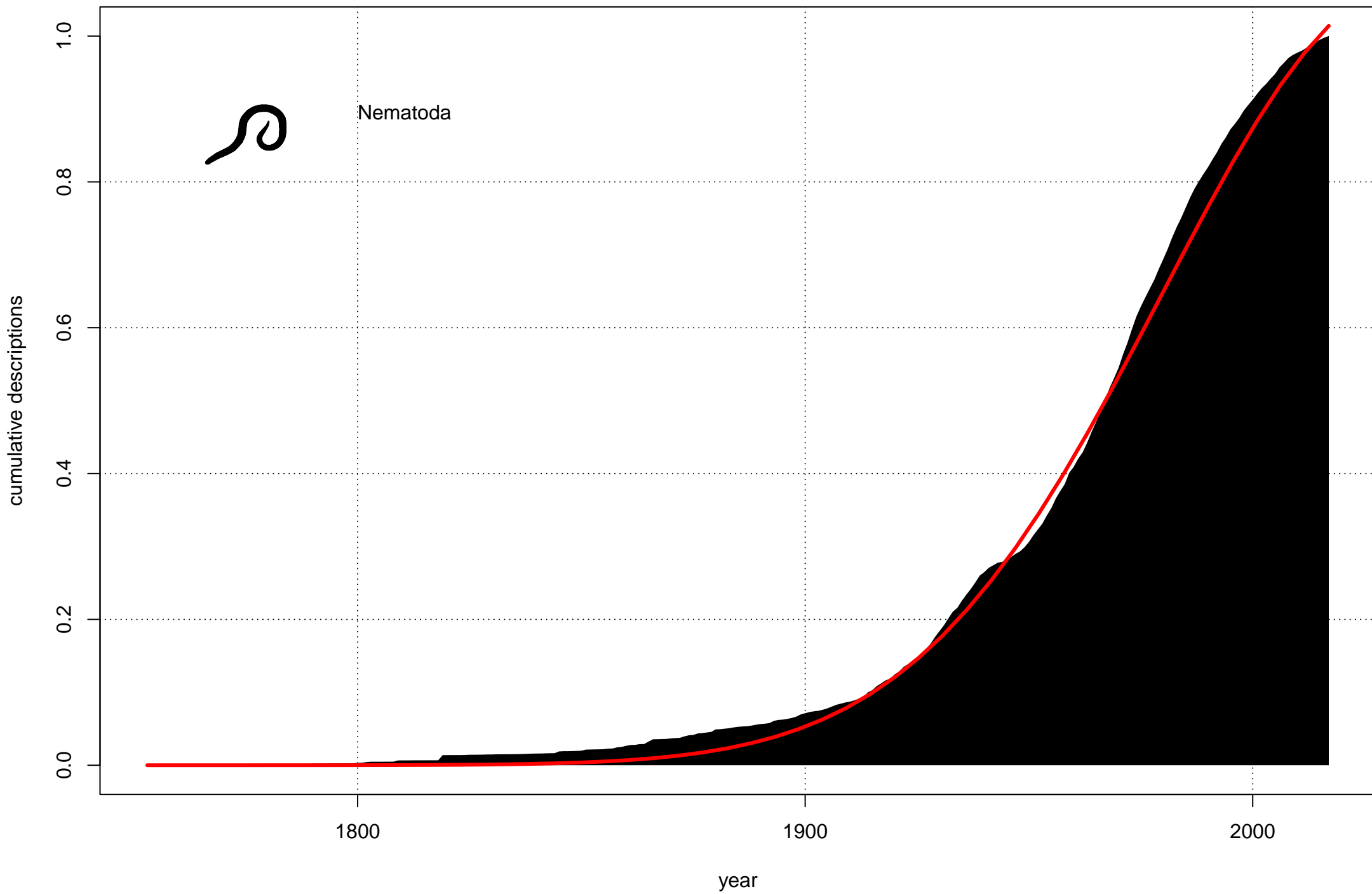

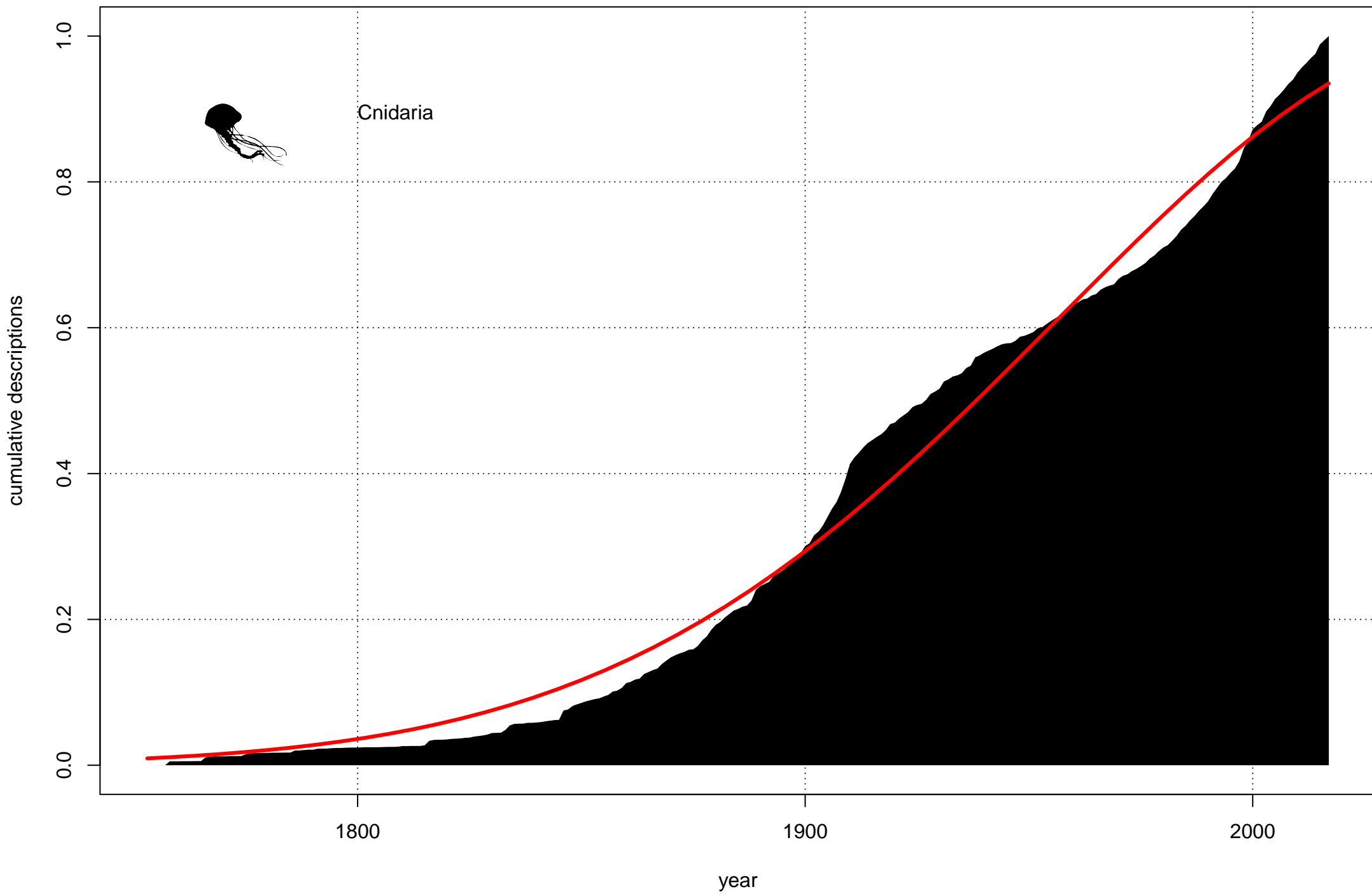

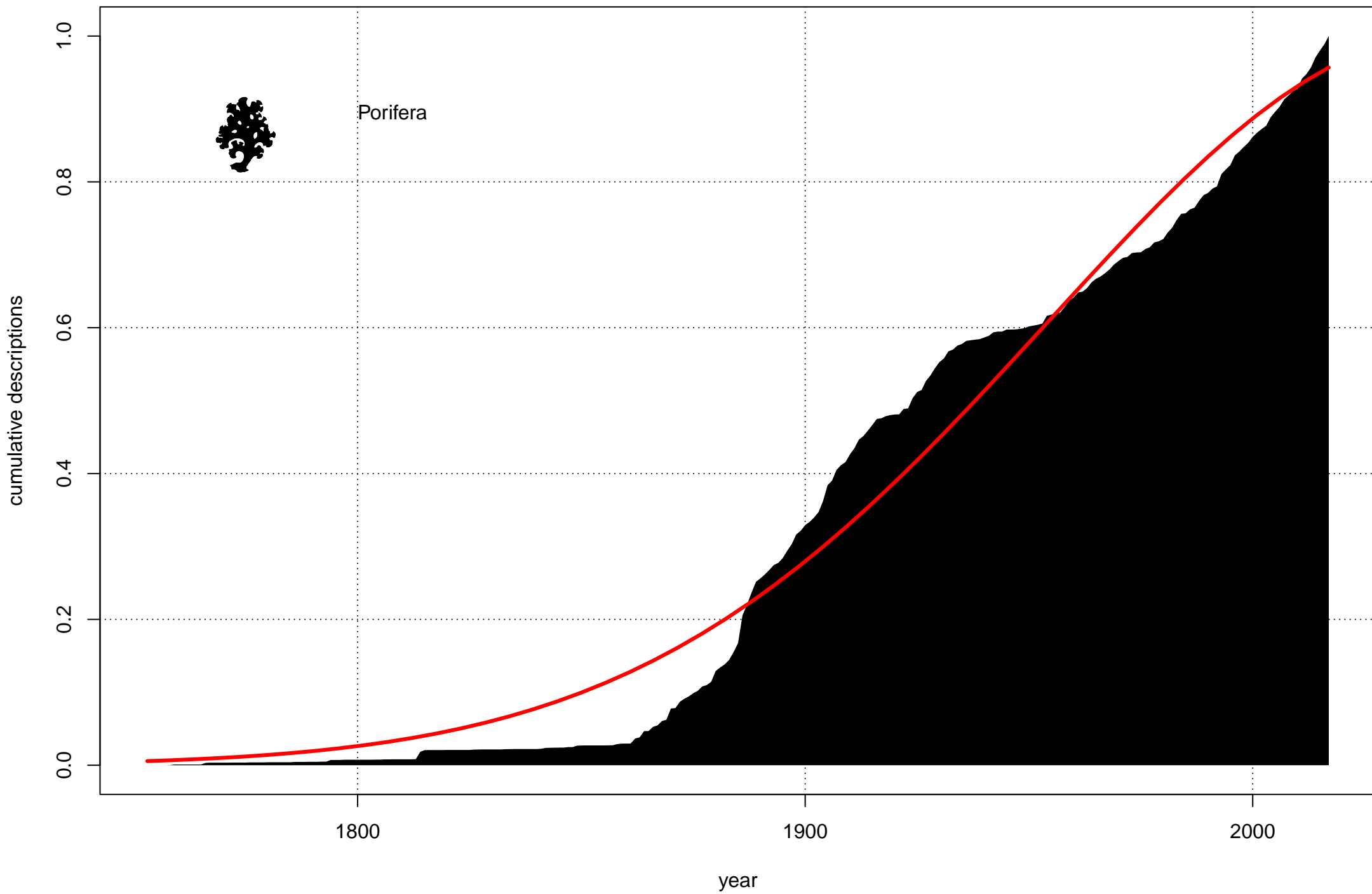

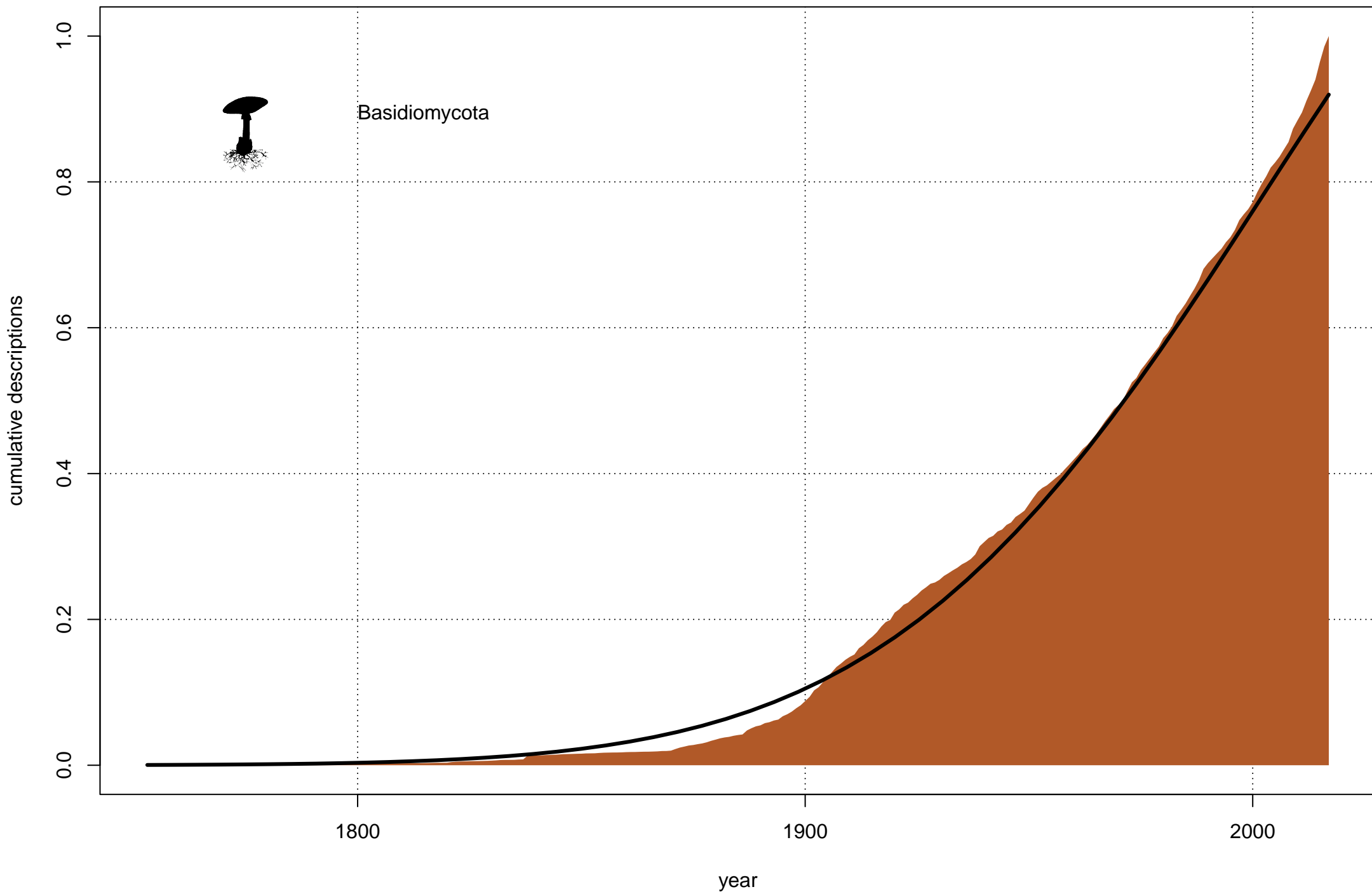

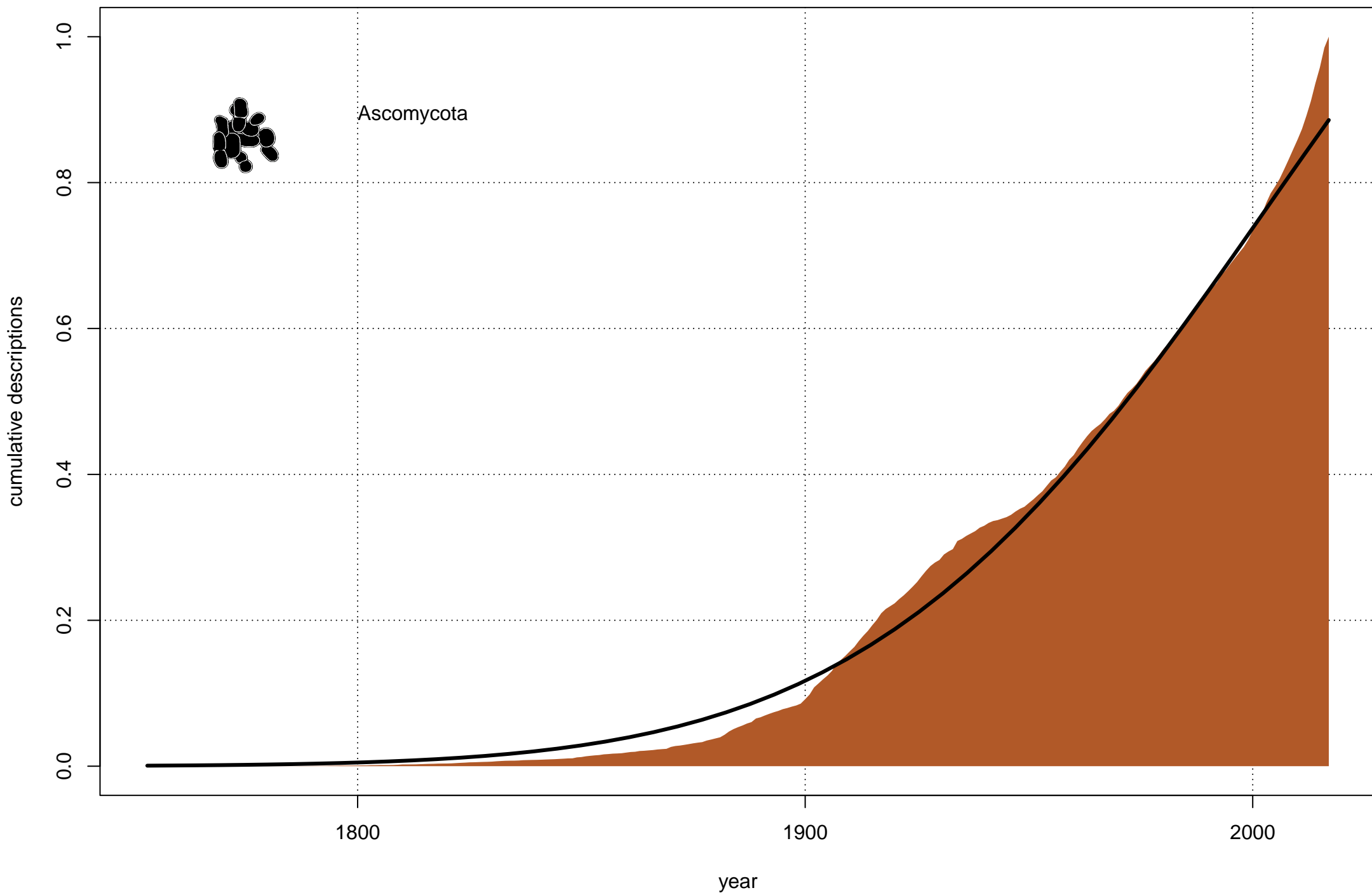

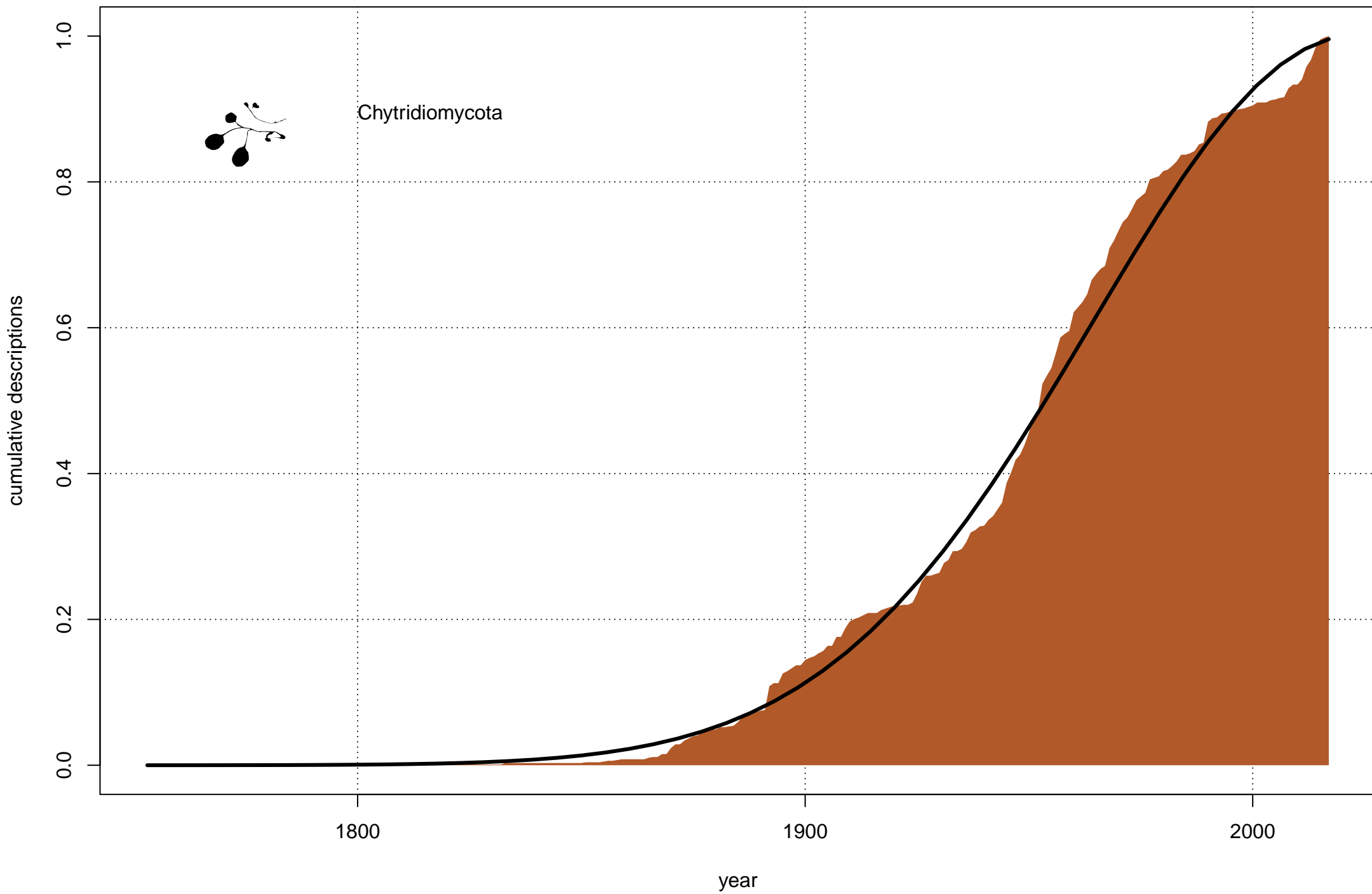

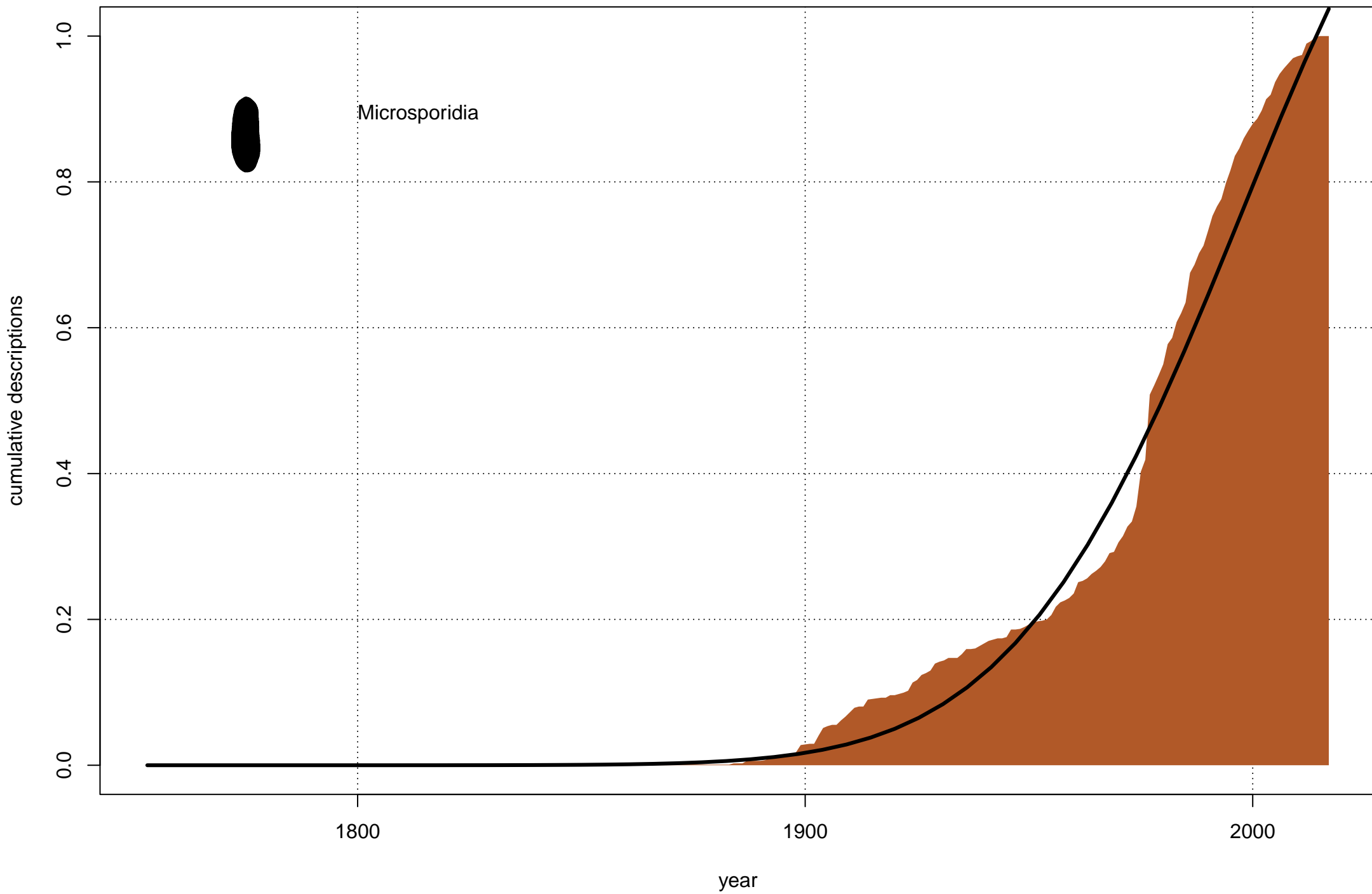

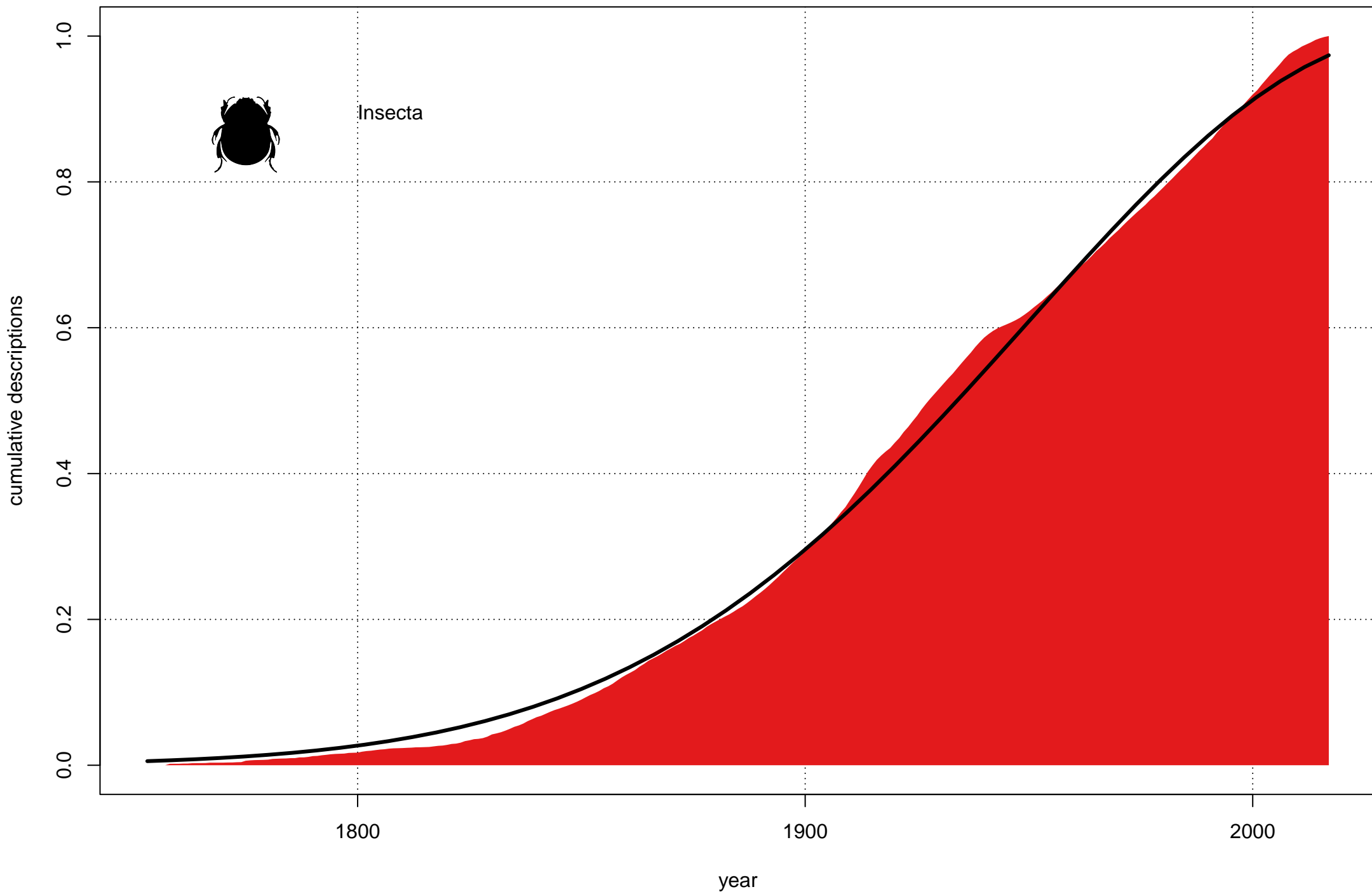

cumulative descriptions

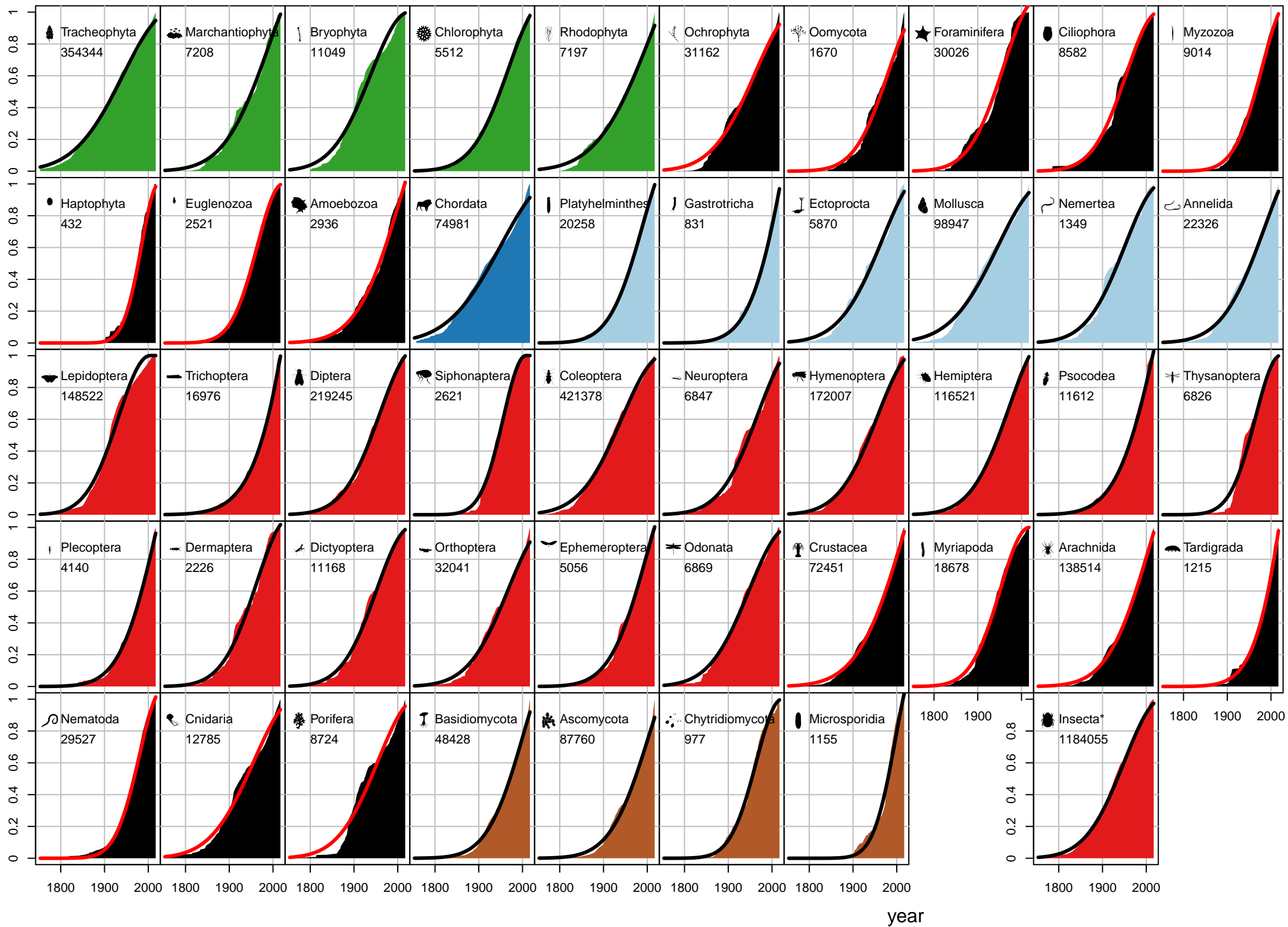

Supplement: Supplementary file 2 — Supplementary Material 2 [file 41598_2025_29845_MOESM2_ESM.pdf]
