# Supplementary material for: Taxonomic description curves of major lineages are influenced by biological and societal factors
Source: Sci Rep. 2025 Nov 24;15:41837. doi: 10.1038/s41598-025-29845-y (PMC12647670; doi:10.1038/s41598-025-29845-y)
Supplement: Supplementary file 3 — Supplementary Material 3 [file 41598_2025_29845_MOESM3_ESM.pdf]

Supplementary Information for "Taxonomic description curves of major lineages are influenced by biological and societal factors"

Supplementary Figure 1: Cumulative description fit example

Left-sided normal distributions were fitted to the data. If reaching a maximum before the end of the time period (2017), curves remained constant after the maximum. The intersect of the fitted curve with the lower horizontal line defines the year when 10% of current descriptions were made. The intersect with the upper horizontal line defines the year when all descriptions were made. The areas filled in red below and above the fitted curve represent the description curve residuals. Distances between empirical and fitted values were squared and summed up as a measure of variability in descriptions over time.

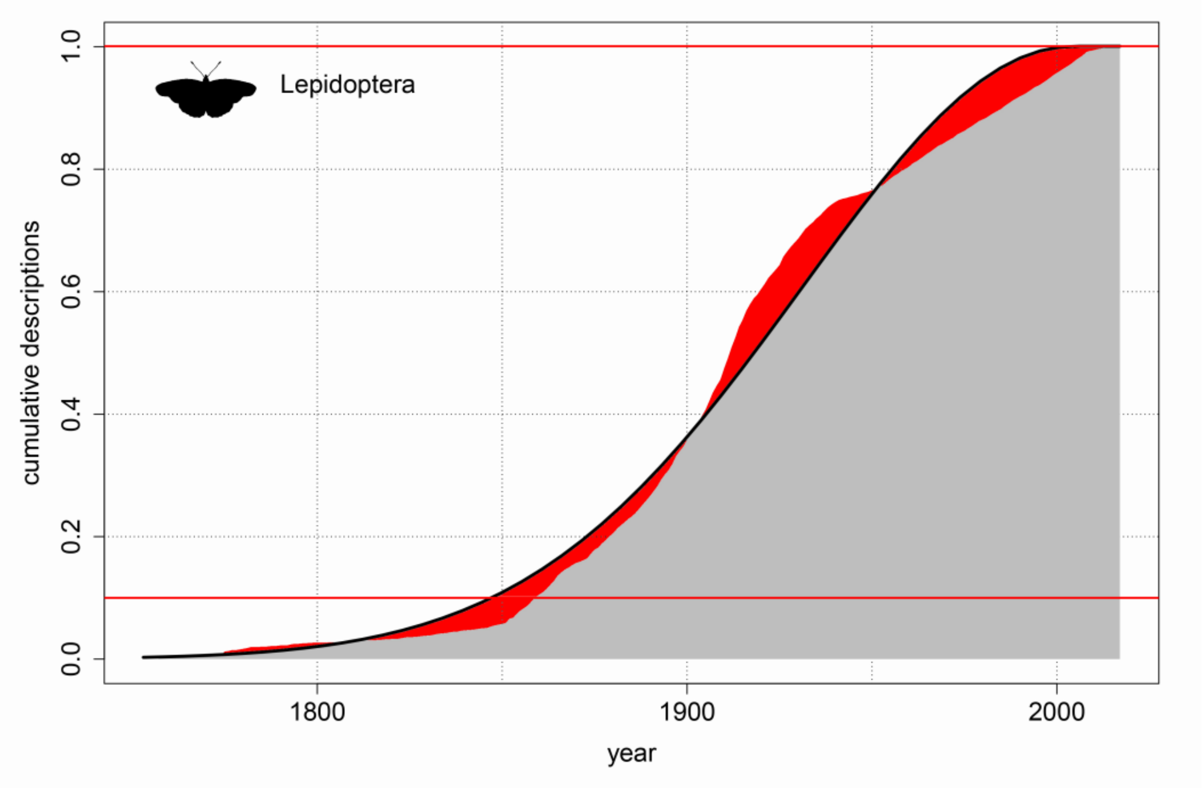

Supplementary Figure 2: Treemap of number of current descriptions, i.e., numbers of species

Area sizes correspond to numbers of species per group. Upper panels: Insects in separate orders, Lower panels: Insects joint in class Insecta. Left: All groups, right: enlargement of groups surrounded by the orange border in the left panel. See Tab. 1 for absolute numbers per group. The upper left and right panels comprise a total of 2,300,484 and 73,458 species, respectively. The lower left and right panels comprise a total of 2,300,484 and 361,883 species, respectively. The fill color of individual groups corresponds to Fig. 1.

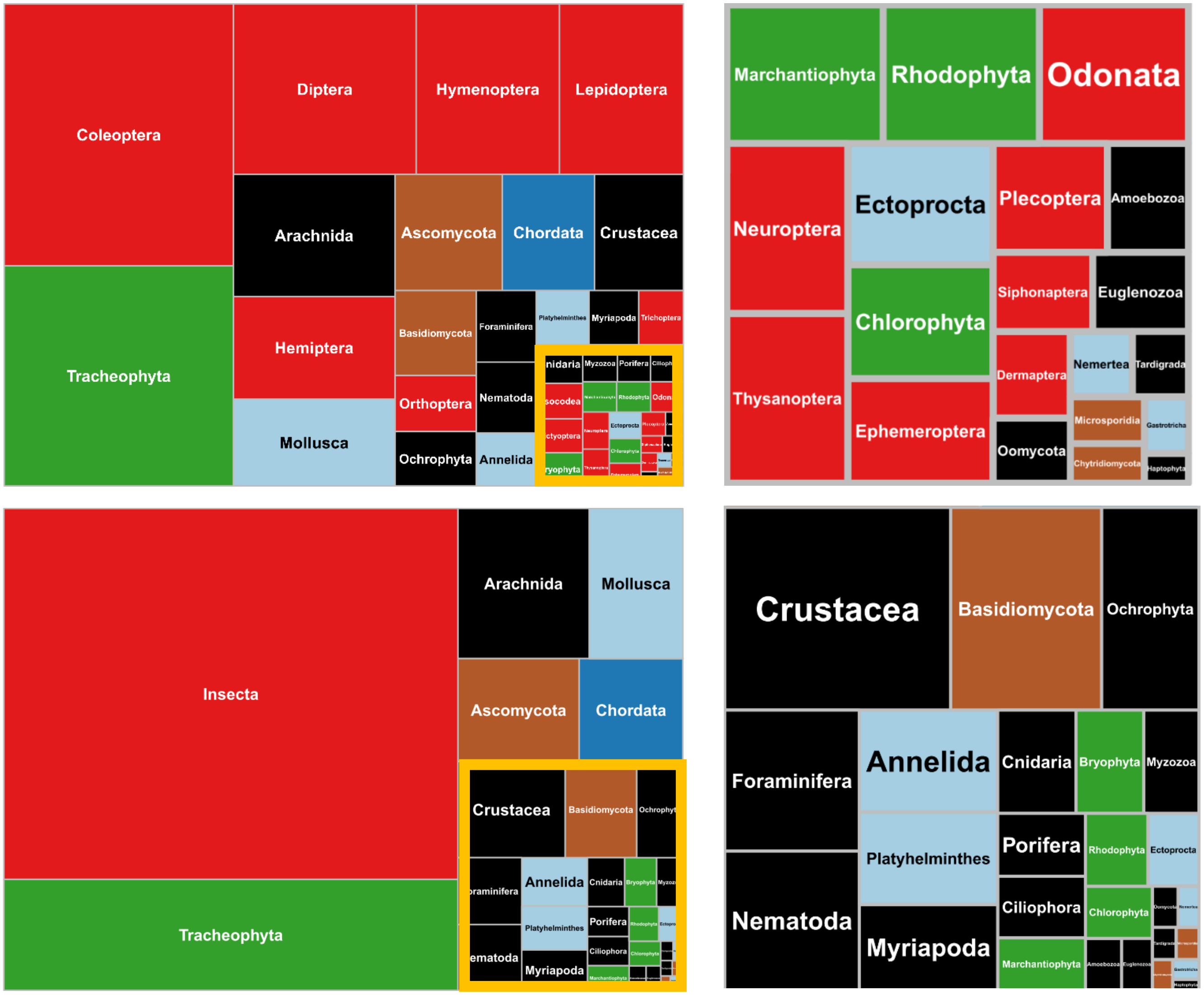

Supplementary Figure 3: Descriptions per year and cumulative descriptions

The time range for both panels is 1753 to 2017. For better comparison, insects have additionally been plotted as a class in the lower right.

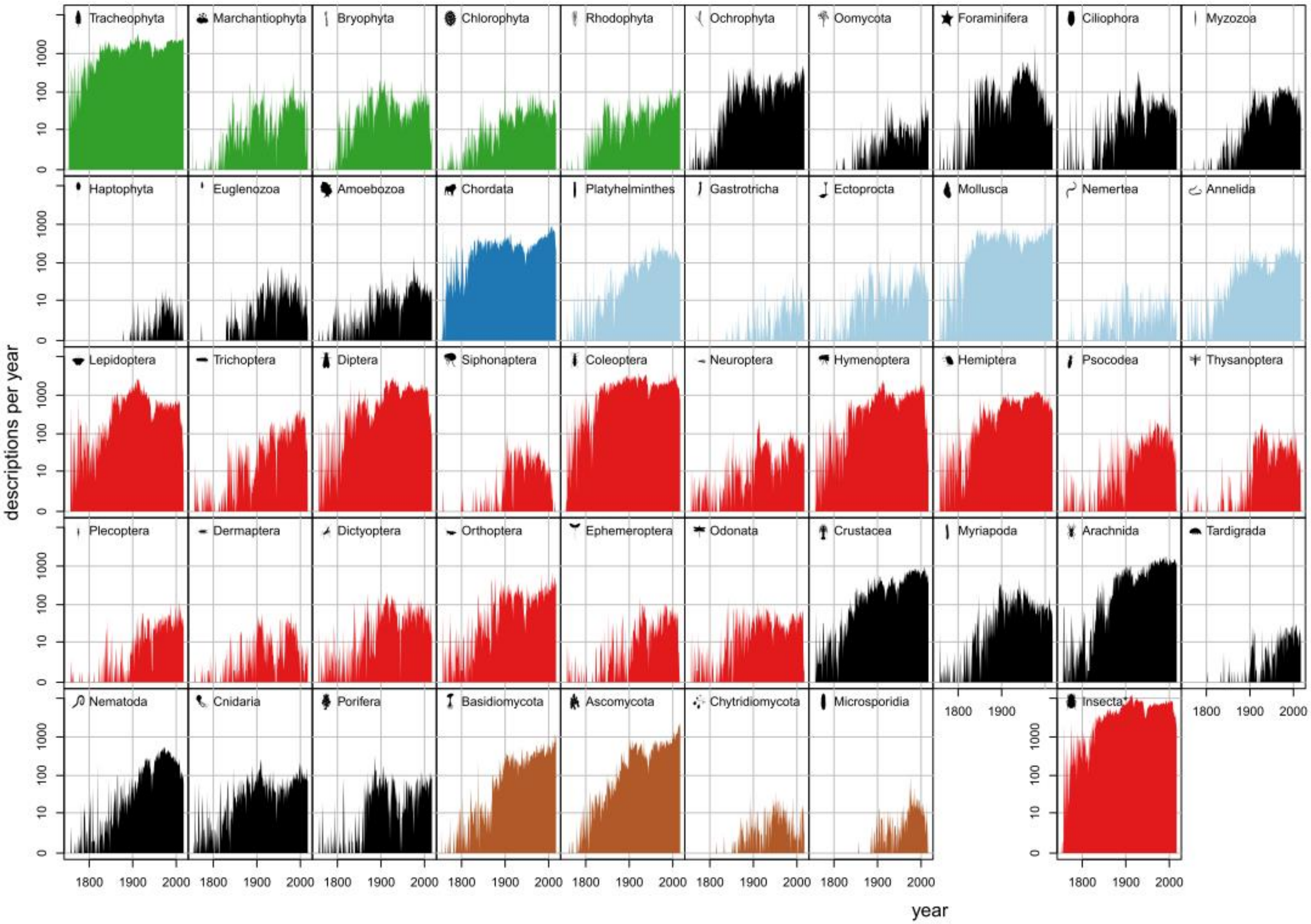

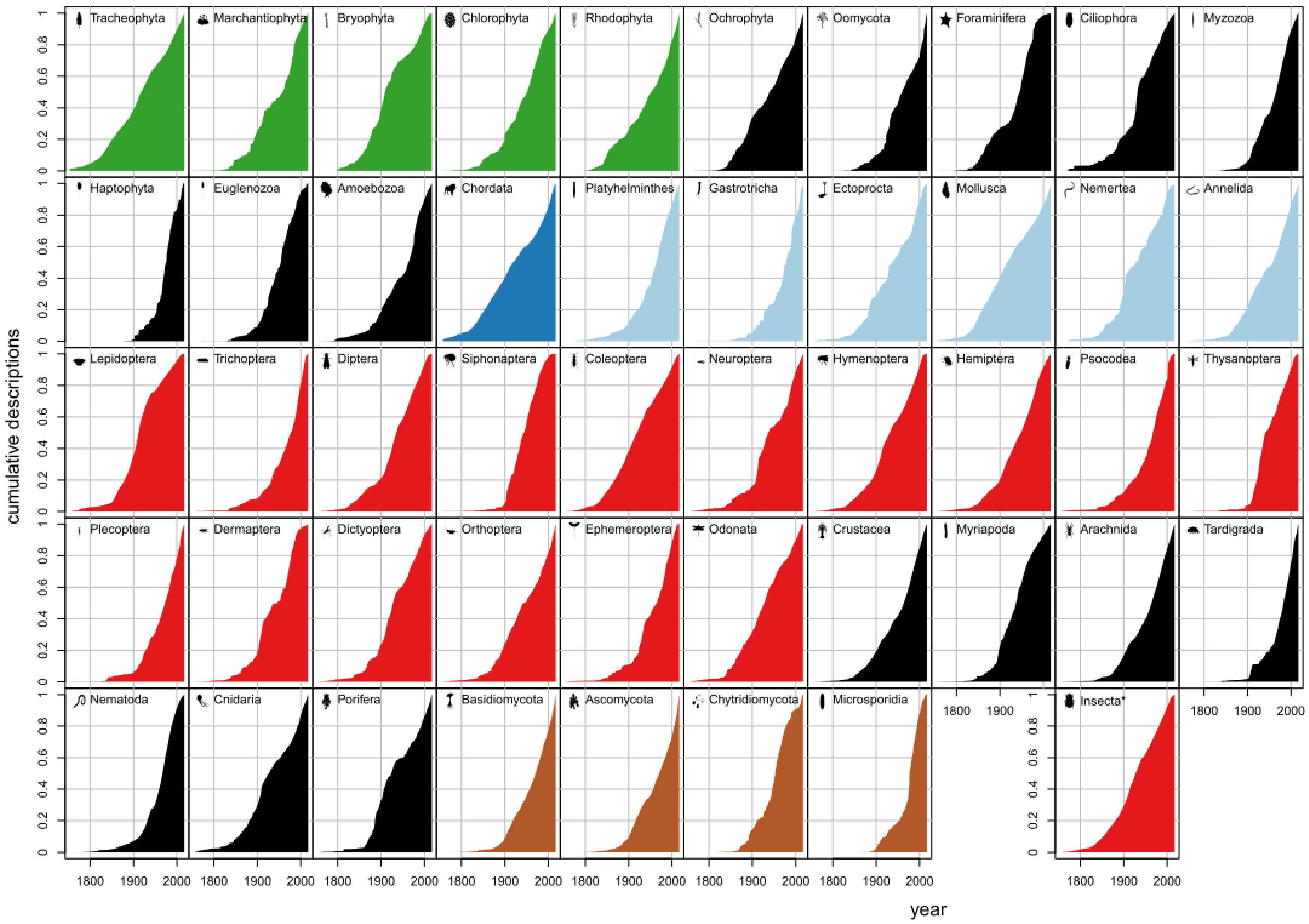
Tracheophyta
Marchantiophyta
Bryophyta
Chlorophyta
Rhodophyta
Ochrophyta
Oomycota
Foraminifera
Ciliophora
Myzozoa
Haptophyta
Euglenozoa
Amoebozoa
Chordata
Platyhelminthes
Gastrotricha
Ectoprocta
Mollusca
Nemertea
Annelida
Lepidoptera
Trichoptera
Diptera
Siphonaptera
Coleoptera
Neuroptera
Hymenoptera
Hemiptera
Psocodea
Thysanoptera
Plecoptera
Dermaptera
Dictyoptera
Orthoptera
Ephemeroptera
Odonata
Crustacea
Myriapoda
Arachnida
Tardigrada
Nematoda
Cnidaria
Porifera
Basidiomycota
Ascomycota
Chytridiomycota
Microsporidia
Insecta*
cumulative descriptions
year
0
0.2
0.4
0.6
0.8
1
1800
1900
2000

Supplementary Figure 4: Cumulative description curve fits

These figures are available as a separate pdf file, containing 49 pages. We fitted half-sided normal distribution functions to the data. When a plateau was reached, a constant function was added to the half-sided normal distribution function.

Supplementary Figure 5: SEM calculated with the class Insecta instead of individual orders. Predictors were calculated as weighted averages of the values of the different insect orders, weighted by species numbers. The corresponding results can be found in Supplementary Table 4.

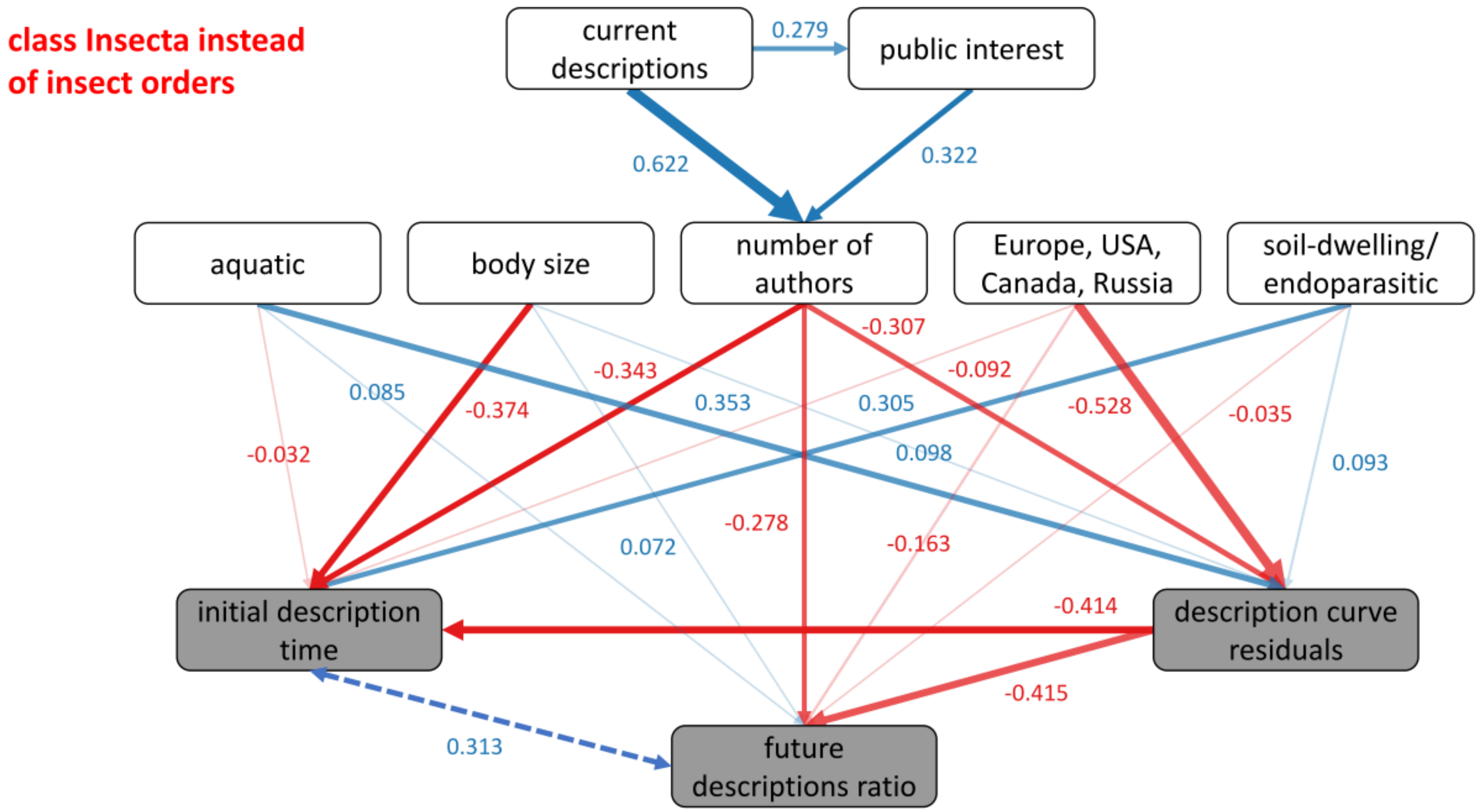

Supplementary Figure 6: Comparison between species numbers of taxonomic groups in Catalogue of Life (CoL), the Global Biodiversity Information Facility (GBIF), and LifeGate. For better comparison, insects have additionally been plotted as a class at the bottom of the graph.

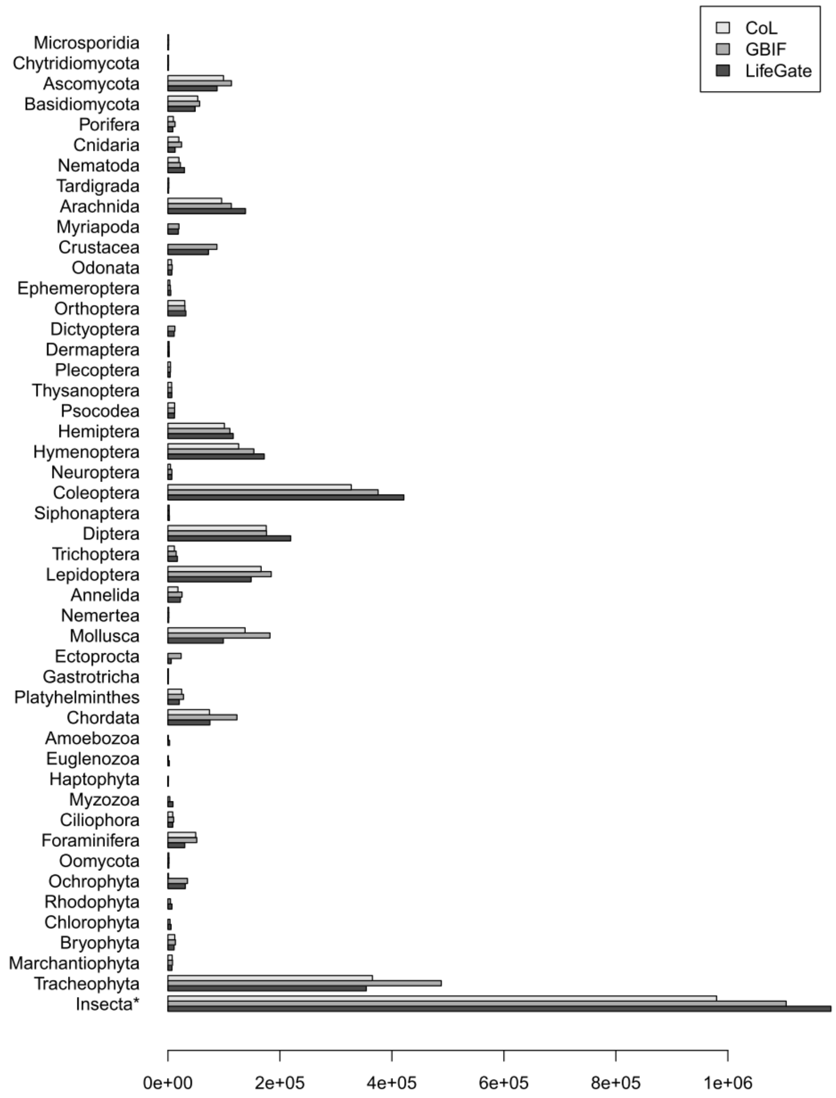

Supplementary Figure 7: Bivariate relationships between selected predictors and description curve parameters

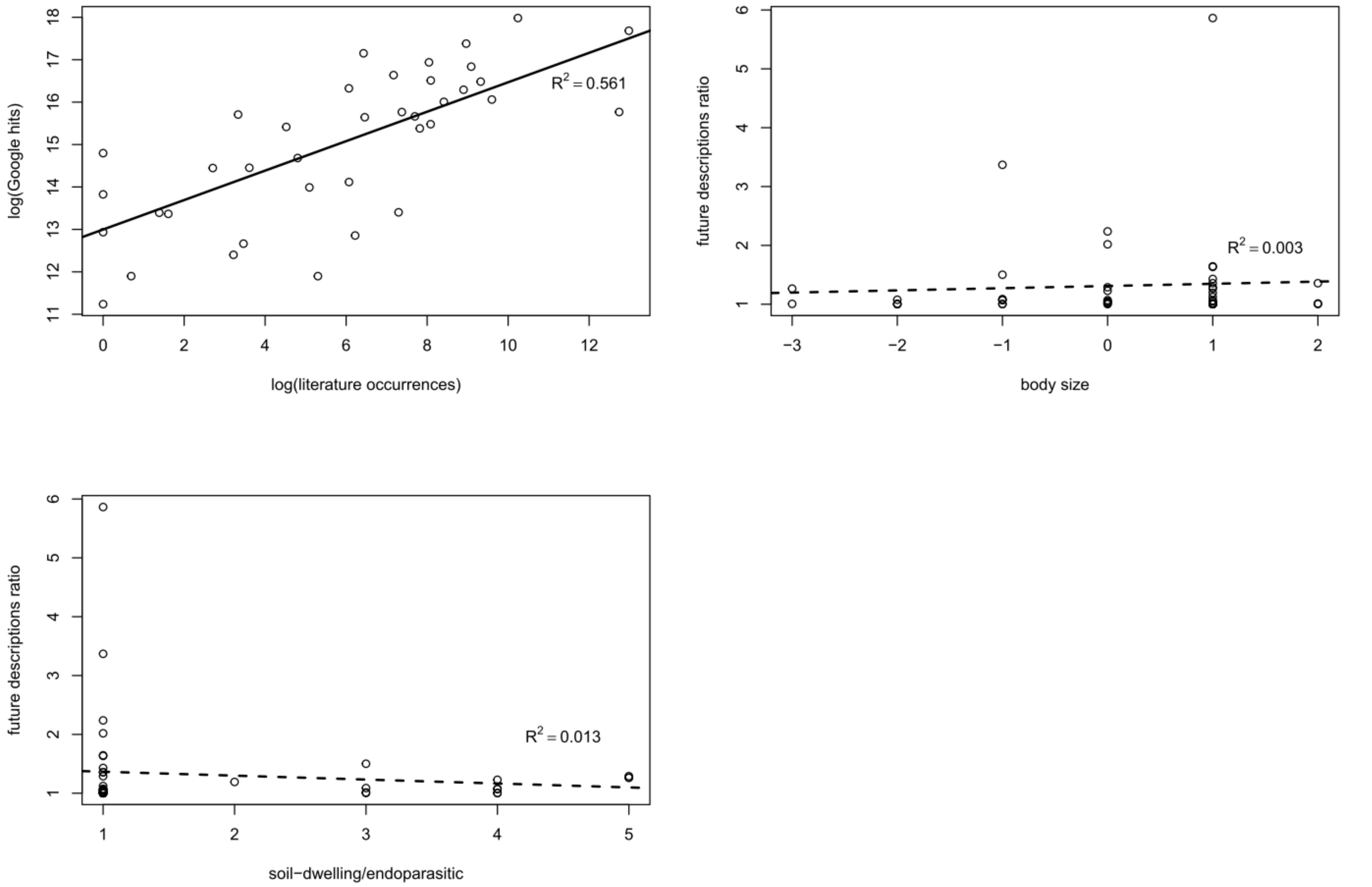

Supplementary Table 1: Predictors sources

This table is available as a separate Excel file, containing three sheets. The sheet “sources” details the websites used to retrieve the information detailed in the “groups” sheet.

Supplementary Table 2: GBIF species numbers

GBIF taxon keys are provided for all taxonomic groups investigated in this study, except Crustacea, Myriapoda and Dictyoptera. These three groups do not exist in GBIF, but we sampled and added occurrences for their respective subgroups. These were Malacostraca, Copepoda, Ostracoda, Branchiopoda, Maxillopoda, Cephalocarida, and Remipedia for Crustacea, Chilopoda, Symphyla, Pauropoda, and Diplopoda for Myriapoda, and Blattodea and Mantodea for Dictyoptera.

The column named “europe_usa_canada_russia” shows the number of species with occurrences in GBIF. The column named “world” shows the number for the whole world. The columns with appended “_coords” show the number of species within the respective regions and the world with occurrences for which coordinates are given. The column “world_coords_ land_and_sea” shows numbers of species with coordinates and occurrences inside and outside of country borders, i.e., in marine areas. The column “ratio_seaborne” is calculated as the ratio between species in world_coords_land_and_sea not present in “world_coords” and “world_coords” itself. A ratio of 0.5 therefore indicates that half as many species are found outside continental borders as there are species within continental borders from a specific taxonomic group. The column “ratio” is the ratio between the columns “europe_usa_canada_russia” and “world”, and the column “ratio_coords” is the ratio between the columns “europe_usa_canada_russia_coords” and “world_coords”.

GBIFTaxonKey	name	europe_usa_canada_russia	world	europe_usa_canada_russia_coords	world_coords	world_coords_land_and_sea	ratio_seaborne	ratio	ratio_coords
7707728	Tracheophyta	116320	379724	83203	331952	332033	0	0.31	0.25
9	Marchantiophyta	1689	6797	1306	6091	6091	0	0.25	0.21
35	Bryophyta	3891	11678	3001	9685	9688	0	0.33	0.31
36	Chlorophyta	2198	2650	1953	2397	2400	0	0.83	0.81
106	Rhodophyta	2790	4090	2534	3839	3843	0	0.68	0.66
98	Ochrophyta	10756	16527	7477	11047	11926	0.1	0.65	0.68
32	Oomycota	949	1208	706	878	878	0	0.79	0.8
8376456	Foraminifera	11687	18582	8645	13831	14137	0	0.63	0.63
7765738	Ciliophora	1632	2181	1533	1883	1923	0	0.75	0.81
8770992	Myzozoa	1340	1874	1229	1723	1755	0	0.72	0.71
70	Haptophyta	373	496	365	489	520	0.1	0.75	0.75
41	Euglenozoa	377	468	354	428	439	0	0.81	0.83
7509337	Amoebozoa	236	309	219	292	293	0	0.76	0.75
44	Chordata	34730	100199	29106	91749	92009	0	0.35	0.32
108	Platyhelminthes	6733	14770	5385	10870	10951	0	0.46	0.5
22	Gastrotricha	319	410	277	363	365	0	0.78	0.76
53	Ectoprocta	7449	12144	6009	10106	10172	0	0.61	0.59
52	Mollusca	62288	132250	51488	109758	110281	0	0.47	0.47
63	Nemertea	471	812	358	616	633	0	0.58	0.58
42	Annelida	8117	17597	7509	15393	15698	0	0.46	0.49
797	Lepidoptera	26322	119763	24884	98641	99036	0	0.22	0.25
1003	Trichoptera	3206	9456	3033	7893	7945	0	0.34	0.38
811	Diptera	36681	93410	32668	68423	71086	0	0.39	0.48
1366	Siphonaptera	449	1214	430	1058	1065	0	0.37	0.41
1470	Coleoptera	51699	154770	45631	119229	120761	0	0.33	0.38
1501	Neuroptera	1143	3852	1070	3303	3363	0	0.3	0.32
1457	Hymenoptera	34523	83453	30456	68488	69309	0	0.41	0.44
809	Hemiptera	21890	77735	16016	43626	45932	0.1	0.28	0.37
7612838	Psocodea	1683	5527	1241	2670	2985	0.1	0.3	0.46
1228	Thysanoptera	1434	4388	1022	2177	2190	0	0.33	0.47
787	Plecoptera	1411	2850	1368	2572	2606	0	0.5	0.53
1224	Dermaptera	174	938	148	730	771	0.1	0.19	0.2
	Dictyoptera	1407	7215	1299	5163	5248	0	0.2	0.25
1458	Orthoptera	3491	16437	3212	11970	12243	0	0.21	0.27
1225	Ephemeroptera	1086	2619	971	2036	2042	0	0.41	0.48
789	Odonata	1434	6442	1341	6113	6121	0	0.22	0.22
	Crustacea	23386	57340	20062	49215	50391	0	0.41	0.41
	Myriapoda	3706	10715	2926	7369	7440	0	0.35	0.4
367	Arachnida	17970	59104	15388	46231	46678	0	0.3	0.33
14	Tardigrada	403	733	322	605	612	0	0.55	0.53
5967481	Nematoda	4990	9646	3849	7397	7839	0.1	0.52	0.52
43	Cnidaria	8801	18178	7862	16480	16657	0	0.48	0.48
105	Porifera	4762	11557	4483	11274	11484	0	0.41	0.4
34	Basidiomycota	25729	45226	22417	35086	35096	0	0.57	0.64
95	Ascomycota	46522	80586	36065	58582	58627	0	0.58	0.62
94	Chytridiomycota	378	476	319	386	386	0	0.79	0.83
7501587	Microsporidia	230	292	70	104	104	0	0.79	0.67

Supplementary Table 3: SEM results

In addition to estimates, we provide standard deviations (Post.SD), upper and lower bounds of the prediction intervals (pi.lower, pi.upper), Rhat values, and priors. Letters correspond to theoretical (outlines) and empirical (posterior sampling) distributions of the coefficients shown in the figure below.

Response	Predictor	Estimate	Post.SD	pi.lower	pi.upper	Rhat	Prior	LETTER
public interest	current descriptions	0.381	0.144	0.099	0.667	1	normal(0,10)	a
number of authors	public interest	0.339	0.053	0.235	0.442	1	normal(0,10)	b
number of authors	current descriptions	0.764	0.054	0.656	0.867	1	normal(0,10)	c
description curve residuals	body size	-0.059	0.210	-0.471	0.356	1	normal(0,10)	d
description curve residuals	number of authors	-0.116	0.164	-0.441	0.207	1	normal(0,10)	e
description curve residuals	Europe, USA, Canada, Russia	-0.550	0.303	-1.150	0.037	1	normal(0,10)	f
description curve residuals	soil-dwelling/endoparasitic	0.128	0.193	-0.257	0.502	1	normal(0,10)	g
description curve residuals	aquatic	0.364	0.225	-0.082	0.794	1	normal(0,10)	h
initial description time	body size	-0.371	0.141	-0.644	-0.090	1	normal(0,10)	ii
initial description time	number of authors	-0.400	0.113	-0.632	-0.179	1	normal(0,10)	j
initial description time	Europe, USA, Canada, Russia	-0.169	0.216	-0.586	0.257	1	normal(0,10)	k
initial description time	soil-dwelling/endoparasitic	0.283	0.132	0.026	0.537	1	normal(0,10)	l
initial description time	aquatic	0.004	0.159	-0.310	0.309	1	normal(0,10)	m
initial description time	description curve residuals	-0.430	0.108	-0.645	-0.222	1	normal(0,10)	n
future descriptions ratio	body size	0.149	0.214	-0.273	0.575	1	normal(0,10)	o
future descriptions ratio	number of authors	-0.245	0.168	-0.587	0.075	1	normal(0,10)	p
future descriptions ratio	Europe, USA, Canada, Russia	0.033	0.327	-0.603	0.691	1	normal(0,10)	q
future descriptions ratio	soil-dwelling/endoparasitic	-0.102	0.199	-0.488	0.290	1	normal(0,10)	r
future descriptions ratio	aquatic	-0.079	0.239	-0.552	0.389	1	normal(0,10)	s
future descriptions ratio	description curve residuals	-0.329	0.160	-0.643	-0.013	1	normal(0,10)	t

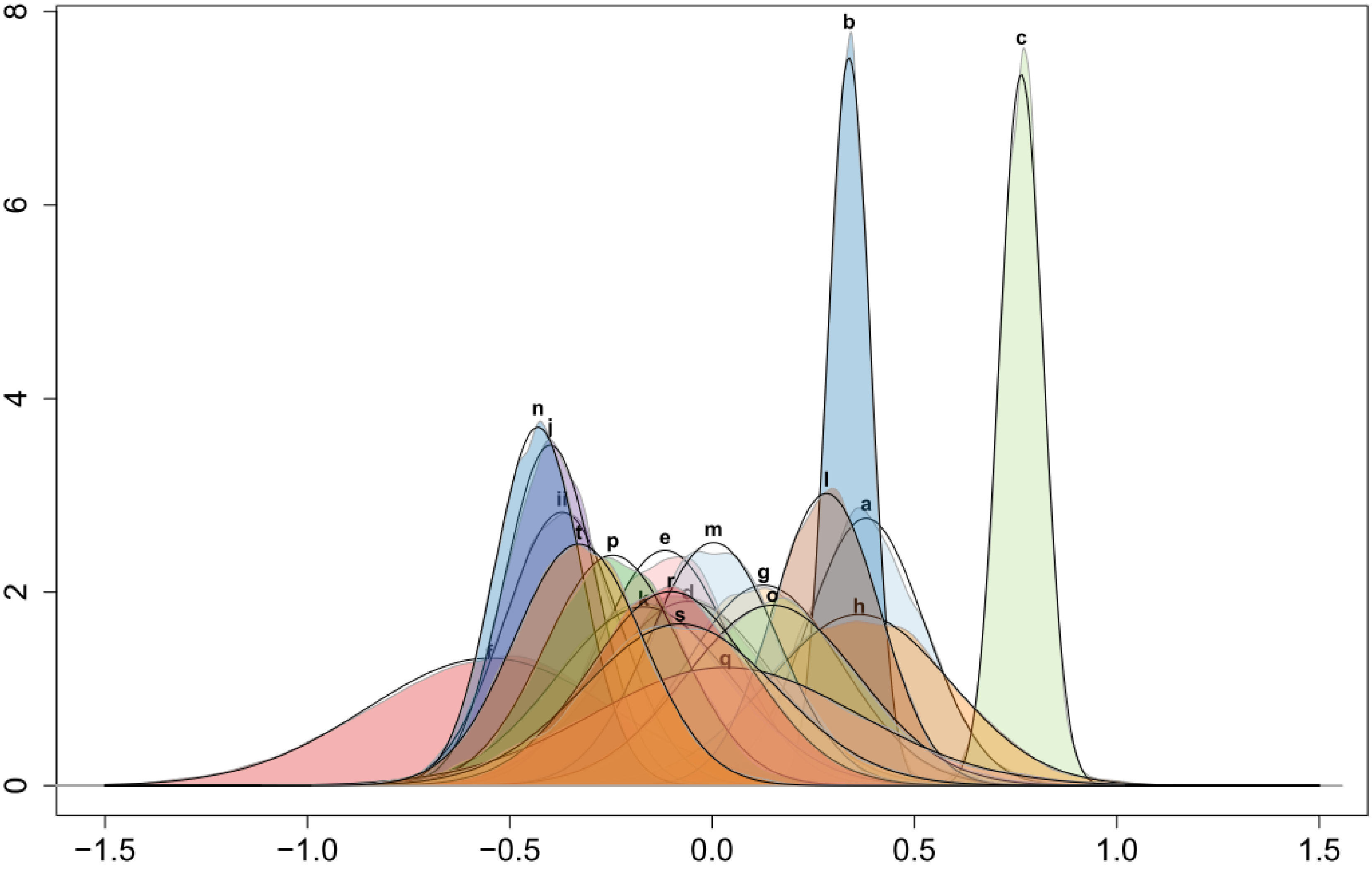

8
6
4
2
0
−1.5
−1.0
−0.5
0.0
0.5
1.0
1.5
b
c
n
j
t
p
e
m
l
a
g
o
h
r
d
s
q

Supplementary Table 4: SEM results using class Insecta instead of insect orders

In addition to estimates, we provide standard deviations (Post.SD), upper and lower bounds of the prediction intervals (pi.lower, pi.upper), Rhat values, and priors. Letters correspond to theoretical (outlines) and empirical (posterior sampling) distributions of the coefficients shown in the figure below.

Response	Predictor	Estimate	Post.SD	pi.lower	pi.upper	Rhat	Prior	LETTER
public interest	current descriptions	0.279	0.187	-0.087	0.648	1	normal(0,10)	a
number of authors	public interest	0.620	0.127	0.361	0.869	1	normal(0,10)	b
number of authors	current descriptions	0.322	0.128	0.073	0.571	1	normal(0,10)	c
description curve residuals	body size	0.098	0.251	-0.403	0.597	1	normal(0,10)	d
description curve residuals	number of authors	-0.307	0.222	-0.738	0.142	1	normal(0,10)	e
description curve residuals	Europe, USA, Canada, Russia	-0.528	0.318	-1.151	0.100	1	normal(0,10)	f
description curve residuals	soil-dwelling/endoparasitic	0.093	0.231	-0.361	0.545	1	normal(0,10)	g
description curve residuals	aquatic	0.353	0.258	-0.165	0.852	1	normal(0,10)	h
initial description time	body size	-0.374	0.170	-0.712	-0.037	1	normal(0,10)	i
initial description time	number of authors	-0.343	0.158	-0.656	-0.035	1	normal(0,10)	j
initial description time	Europe, USA, Canada, Russia	-0.092	0.221	-0.528	0.349	1	normal(0,10)	k
initial description time	soil-dwelling/endoparasitic	0.305	0.155	0.004	0.606	1	normal(0,10)	l
initial description time	aquatic	-0.032	0.179	-0.385	0.319	1	normal(0,10)	m
initial description time	description curve residuals	-0.414	0.142	-0.698	-0.136	1	normal(0,10)	n
future descriptions ratio	body size	0.072	0.270	-0.452	0.606	1	normal(0,10)	o
future descriptions ratio	number of authors	-0.278	0.248	-0.774	0.209	1	normal(0,10)	p
future descriptions ratio	Europe, USA, Canada, Russia	-0.163	0.348	-0.848	0.531	1	normal(0,10)	q
future descriptions ratio	soil-dwelling/endoparasitic	-0.035	0.244	-0.527	0.442	1	normal(0,10)	r
future descriptions ratio	aquatic	0.085	0.281	-0.472	0.650	1	normal(0,10)	s
future descriptions ratio	description curve residuals	-0.415	0.221	-0.852	0.021	1	normal(0,10)	t

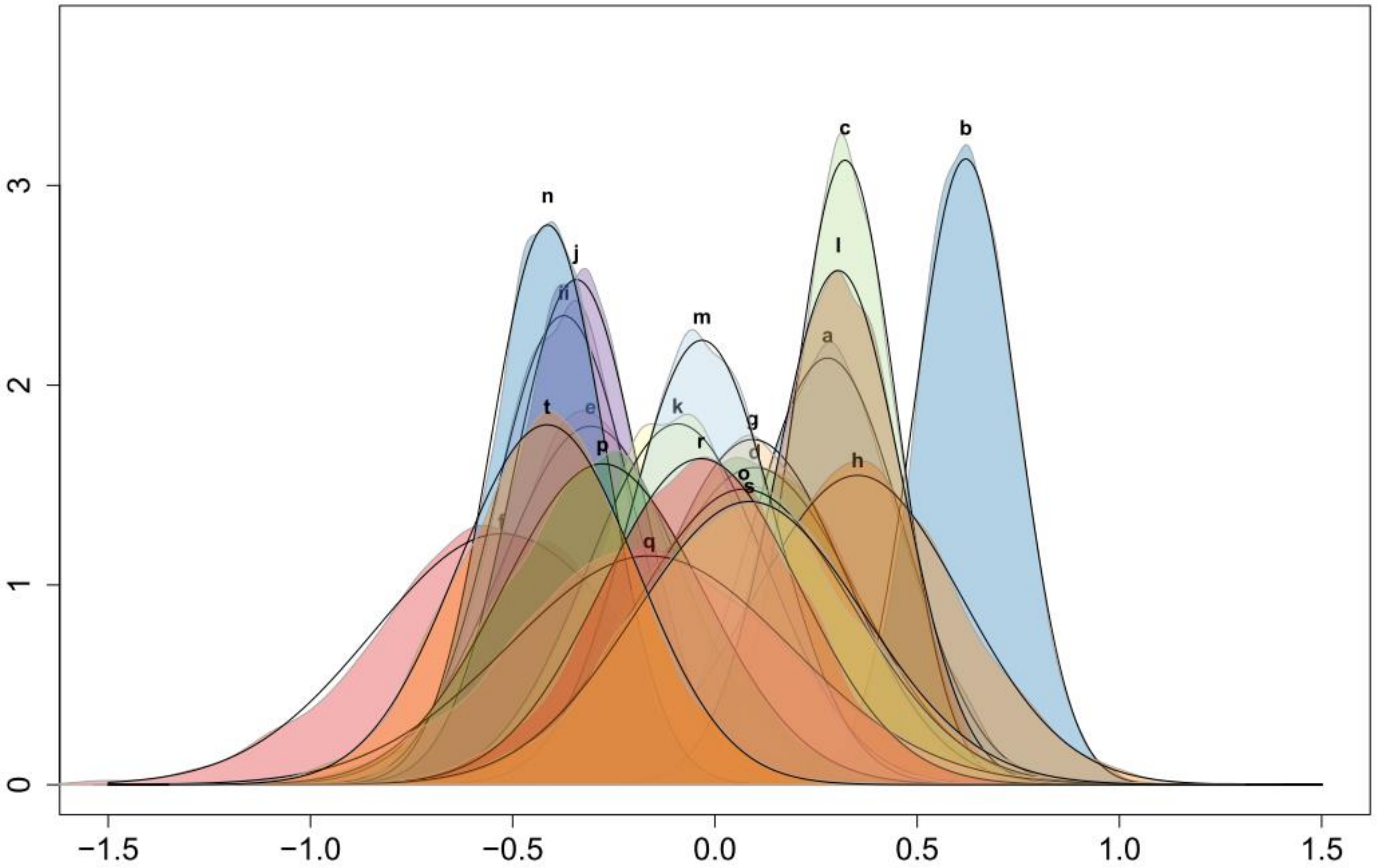
c
b
n
l
j
i
m
a
k
t
e
g
p
r
d
o
s
h
f
q
0
1
2
3
−1.5
−1.0
−0.5
0.0
0.5
1.0
1.5
